# Exploring the Predictors of Nurses’ Turnover Intentions Through Neural Network Modeling: A National Cross-Sectional Study in Lithuania

**DOI:** 10.3390/healthcare14070831

**Published:** 2026-03-24

**Authors:** Arūnas Žiedelis, Jurgita Lazauskaitė-Zabielskė, Natalja Istomina, Rita Urbanavičė, Jelena Stanislavovienė

**Affiliations:** 1 Institute of Psychology, Vilnius University, 01513 Vilnius, Lithuania; jurgita.lazauskaite@fsf.vu.lt; 2 Institute of Health Sciences, Vilnius University, 01513 Vilnius, Lithuania; natalja.istomina@mf.vu.lt (N.I.); rita.urbanavice@mf.vu.lt (R.U.); jelena.stanislavoviene@mf.vu.lt (J.S.)

**Keywords:** turnover intentions, nurse retention, neural network modeling

## Abstract

**Highlights:**

**What are the main findings?**

- Seven key predictors identified using ANN explained 49.8% of variance in nurses’ turnover intentions, with age, burnout, and job satisfaction emerging as the strongest factors.
- Managerial support, interpersonal conflict, development opportunities, and role conflict showed complex and partly nonlinear effects on turnover intentions.

**What are the implications of the main findings?**

- Retention strategies should focus on reducing burnout and improving job satisfaction, especially among younger nurses, through organizational-level interventions.
- Investing in supportive leadership, clear role definitions, and at least minimal professional development opportunities can significantly reduce nurses’ intentions to leave.

**Abstract:**

**Background/Objectives**: Nurses’ turnover intentions are strong predictors of actual turnover, which increases costs, reduces care quality, and destabilies the workforce. This study aimed to identify the key predictors of nurses’ turnover intentions using advanced machine learning methods and to explore how demographic, well-being, and work environment factors contribute to these intentions. **Methods**: Cross-sectional data were collected from 2459 nurses employed across various healthcare institutions. We used multichannel invitation and snowball sampling. An artificial neural network regression model was applied, combined with iterative feature selection and SHAP analysis, to identify the most important predictors of turnover intentions and to examine nonlinear and context-dependent relationships among variables. **Results**: Seven predictors explained 49.8% of the variance in turnover intentions, outperforming traditional linear models. Age was the strongest predictor, with younger nurses demonstrating a substantially higher likelihood of intending to leave; this association was nonlinear, with intentions decreasing more sharply at older ages. Job satisfaction and burnout were also strong predictors, particularly among younger nurses. Four work environment factors further contributed to turnover intentions: managerial support functioned as a protective factor, interpersonal conflict increased intentions to leave, limited professional development opportunities were strongly associated with higher turnover intentions, and role conflict showed heterogeneous effects. **Conclusions**: Machine learning approaches enhance understanding of complex workforce dynamics and enable more precise identification of high-risk groups. The findings support age-sensitive retention strategies, proactive monitoring of nurse well-being, and organizational interventions to strengthen managerial support and professional development, ensuring workforce stability and sustainable healthcare service delivery.

## 1. Introduction

Health systems rely on the capacity and effectiveness of their workforce, with nurses representing the largest segment of healthcare professionals. According to projections by the World Health Organization [1], the global shortage of health workers is expected to reach approximately 11 million by 2030, disproportionately affecting low- and lower-middle-income countries. Within this global context, the concept of turnover intentions—defined as an individual’s conscious and deliberate intention to leave their current job or organization—has gained considerable scholarly attention in nursing research [2]. Turnover intentions are regarded as a proximal predictor of actual turnover behavior and thus serve as an important early indicator of workforce instability [3]. Empirical investigations into turnover intentions have sought to reveal the psychological, organizational, and environmental determinants that shape nurses’ plans to change jobs [4]. By understanding and addressing the factors that influence nurses’ turnover intentions, healthcare organizations can foster healthier work environments that reduce attrition and thus enhance the quality of patient care [5].

Although the factors influencing nurses’ turnover intentions have been widely studied, most analyses typically rely on classical linear regression models. This approach has several limitations. Linear regression assumes linear relationships, potentially overlooking meaningful non-linear associations between predictors and the outcome [6]. It also requires specifying interactions a priori, which is often unrealistic in complex social phenomena, whereas machine learning algorithms can detect such interactions directly from the data [7]. Additionally, linear models are sensitive to multicollinearity and heteroskedasticity, while machine learning models are generally more robust to these issues [8]. Owing to their ability to model complex patterns, machine learning methods often achieve higher predictive accuracy than traditional regression approaches [7].

Despite the growing application of machine learning in health research, its use in examining predictors of nurses’ turnover intentions remains limited. By applying ML models to this context, our study addresses this gap. It provides a more comprehensive, data-driven understanding of the factors associated with nurses’ intentions to leave their jobs.

### 1.1. Nurses’ Turnover Intentions

Turnover intention refers to employees’ willingness to leave their workplaces voluntarily [2]. This intention is often triggered by negative psychological states resulting from a perception of an unfavorable internal or external environment. Over time, these negative emotions can lead to cognitive and behavioral patterns of withdrawal, ultimately resulting in resignation [2]. While there is a notable gap between turnover intentions and actual turnover rates [9], intentions remain the most immediate indicator of actual turnover behavior [3].

When nurses leave their jobs, the healthcare sector incurs both direct (i.e., new staff recruitment and training) and indirect costs (e.g., lower productivity and service quality). High nurse turnover is linked to poorer care quality, an increased risk of adverse patient outcomes (including higher mortality rates, more patient falls, and medication errors), and decreased patient satisfaction [5]. Recent research by Bae et al. [10] showed a clear negative correlation between staff turnover and quality of care, even after accounting for other demographic and work-related factors. High turnover also burdens remaining staff, creating a cycle of stress that prompts even more exits [11]. Therefore, healthcare organizations must address nurse turnover through effective retention strategies.

In research, turnover intentions are sometimes assessed dichotomously, categorizing individuals as either intending to change jobs or not [12,13]. Although this approach simplifies interpretation, it has notable theoretical and methodological limitations. Theoretically, intention is a psychological–cognitive construct that varies in strength [3], making it artificial to group all employees considering leaving into a single category. Methodologically, dichotomizing a continuous variable leads to a loss of information [14] and may artificially inflate the predictive power of regression models in which turnover intentions serve as the dependent variable. Therefore, in this study, turnover intentions are treated as a continuous construct varying in strength.

### 1.2. Predictors of Nurses’ Turnover Intentions

Research consistently shows that a variety of individual, organizational, and sociodemographic factors influence nurses’ intention to leave their jobs [4]. Among the individual factors, burnout and job dissatisfaction have received significant attention. Numerous studies indicate that nurses are more likely to quit their positions when they experience burnout or are dissatisfied with their work [15,16,17]. Additionally, more distant factors related to turnover intentions, such as workload stressors and interpersonal relationships, have been extensively studied. Research consistently shows that nurses are more likely to consider leaving their jobs when they face a high workload [15,16], encounter staff shortages [17], or feel pressured to perform tasks not directly related to nursing [17]. Workplace conflicts and bullying have also been identified as significant factors contributing to thoughts of changing jobs [18,19]. Conversely, studies highlight the protective role of job resources, particularly social support [20]. Finally, research indicates differences in sociodemographic characteristics between nurses who intend to leave their jobs and those who do not. Some studies show that younger nurses [20], unmarried nurses [18], and male nurses [19] are more likely to express intentions to leave. However, this trend may be significantly influenced by work environment factors that vary across different sociodemographic groups of nurses [19,21].

Taken together, these findings can be conceptually integrated using the Job Demands–Resources (JD-R) framework [22], which provides a widely used theoretical model for explaining employee well-being and turnover-related outcomes. According to the JD-R model, job characteristics can broadly be categorized into job demands (i.e., aspects that require sustained effort) and job resources (i.e., aspects that help employees to cope with demands). Within this framework, employee well-being indicators—such as burnout, job satisfaction, perceived health, and work ability—can be understood as proximal outcomes of the balance between demands and resources and as key mechanisms linking work environment characteristics to turnover intentions. At the same time, sociodemographic characteristics may influence how nurses experience and respond to workplace conditions [22]. Guided by the JD-R perspective, the present study incorporates variables representing job demands, job resources, well-being, and sociodemographic characteristics to capture the complex, potentially nonlinear relationships underlying nurses’ turnover intentions.

Given that various individual and environmental factors influence nurses’ turnover intentions [4,11], identifying the most important factors is crucial in practice, even though it poses serious methodological challenges. Traditionally, studies have utilized linear or logistic regression models to analyze data from nurses and other employee groups. While these methods are commonly used in social research and offer valuable insights, there has been a growing interest in employing machine learning (ML) algorithms for data analysis across various scientific disciplines [23,24,25]. Initially, ML algorithms were primarily applied to tasks such as speech recognition and image classification [26]. However, they are increasingly being used to address classification and prediction tasks with tabular data [25]. A range of ML algorithms, including random forests, gradient boosting, neural networks, and support vector regression, often outperform linear regression on complex or nonlinear datasets [7]. ML algorithms can model intricate relationships and interactions that linear regression may not adequately capture [6]. These algorithms excel in situations involving nonlinear, heteroscedastic, or high-dimensional data where the assumptions of linear regression—such as linearity, homoscedasticity, and independence—are not met. Furthermore, they can automatically learn complex patterns and interactions without explicit feature engineering [7,8]. As a result, ML algorithms are better suited to analyzing complex social realities, where phenomena are interconnected and often nonlinear.

### 1.3. Applying Machine Learning to Predict Nurses’ Turnover Intentions

To our knowledge, only three studies have examined factors influencing nurses’ turnover intentions using machine learning. Kim et al. [12] developed a random forest model that accurately predicted nurses’ turnover intentions, identifying salary as the most significant factor. Xu et al. [27] created a SMOTE-enhanced random forest model, highlighting age as the most important factor, followed by working hours, electronic medical record usability, income, and region. Finally, Park et al. [13] developed a model based on extreme gradient boosting, finding that job security, job satisfaction, and person-job fit were the most important predictors.

It is important to note that the studies discussed focused on samples of nurses from Korea [12,13] and the United States [27], which limits their applicability to the European context due to differences in cultural and economic circumstances. For instance, nursing roles and professional structures vary significantly between the United States and Europe, particularly in terms of autonomy, regulation, and professional development. In the United States, Advanced Practice Nurses (APNs) often enjoy a high degree of autonomy, which includes prescribing authority and, in many states, the ability to practice independently [28]. This expanded role is partly a response to gaps in primary care and historically high rates of uninsured patients, leading to an increased demand for accessible healthcare providers. Conversely, many European APNs face role ambiguity, limited legal recognition, and underutilization of their skills compared to their North American counterparts [29]. The regulatory frameworks differ as well: U.S. nursing regulations are state-based and pluralistic, resulting in varied scopes of practice and multiple educational pathways. In contrast, European nursing education is influenced by EU directives that standardize basic Registered Nurse preparation but leave advanced roles and specializations less uniformly regulated [29]. Additionally, several European countries—especially in Eastern and some Southern regions—are grappling with nursing shortages, lower salaries, fewer career progression opportunities, and slower professionalization [30]. These factors further contribute to the differences in the status and development of the nursing profession between the two regions. Therefore, when examining nurses’ working conditions and retention, it is essential to consider the local context.

In this study, we employed an artificial neural network (ANN) regression model. It is a type of machine learning model designed to predict continuous numerical values by simulating the information processing of the human brain [31,32]. The model consists of layers of interconnected nodes, or “neurons”, where each connection has a weight that adjusts as the model “learns” from the data. During training, the network identifies complex patterns and relationships between input variables and the target output through backpropagation, which minimizes prediction errors [33].

Although ANNs and machine learning models, in general, have long faced criticism for being “black boxes”—meaning the limited ability to interpret how decisions are made—this issue is addressed by SHAP (i.e., SHapley Additive exPlanations) analysis. SHAP analysis is a method for explaining how machine learning models, including ANNs, make predictions [34]. It is based on Shapley values from cooperative game theory, which measure each feature’s contribution to the final prediction. In the context of ANN models, SHAP helps interpret complex, nonlinear relationships by quantifying the impact of each input variable on the output. This makes the model’s behavior more transparent and trustworthy, allowing researchers and practitioners to understand the reasons behind specific predictions [35]. This is crucial in our case, where the objective is not merely to create a model that predicts nurses’ turnover intentions but to identify the prognostic factors most relevant to decision-making about job changes.

Our research aimed to identify the most important predictors of nurses’ turnover intentions by leveraging advanced machine learning capabilities, specifically artificial neural networks [6].

## 2. Materials and Methods

### 2.1. Design

This study was conducted as a cross-sectional, non-interventional survey. An online survey was conducted from July 2025 to September 2025 using multichannel invitation and snowball sampling. Personal healthcare institutions (primary healthcare centers, outpatient clinics, mental health centers, polyclinics, hospitals) that have contracts with the Territorial Health Insurance Funds were sent an electronic invitation to participate in the survey, with a request for cooperation in distributing the questionnaires. In collaboration with chief nursing administrators, paper questionnaires were distributed in sealed envelopes at healthcare facilities to nurses who requested the survey in paper form. Invitations to participate in the survey were also distributed on Facebook. Before starting the study, permission was obtained from the psychological research ethics committee at Vilnius University (permit no. (1.13 E) 250000-KT-176). At the beginning of the questionnaire, an informed consent form was included, outlining the purpose of the study, how the collected data would be used, and the option for participants to decline involvement in the study.

### 2.2. Participants

All nurses working in Lithuania were invited to the survey. Inclusion criteria were having a nursing license and working in nursing at any healthcare facility in Lithuania. Under the exclusion criteria fell nurses not engaged in nursing practice and nursing students. The registered nurse population in Lithuania was 22,000 (excluding nursing assistants). The Paniotto formula was used to calculate the sample size of nurses, with a statistical significance level of 0.01. The sample size was determined to be at least 647 nurses based on this value.

2459 nurses participated in the study and met the inclusion criteria. 98.8% of study participants were female and represented various levels of healthcare institutions. Of the respondents, 31.1% worked in primary healthcare (596 in a doctor’s team and 167 in palliative care), 33.4% in secondary healthcare institutions (172 in outpatient care and 648 in inpatient care), and 33.2% in tertiary healthcare (107 in outpatient care and 707 in inpatient care). Sixty-two nurses did not indicate the level of their institution or selected the option “other.” The majority (71.3%) of respondents worked in one of the country’s major cities. In terms of specialization, most study participants were general practice nurses (43.5%), followed by anesthesia and intensive care nurses (19%), and community nurses (16.4%). The average age of the study participants was 48.4 years (SD = 12.2), and the average work experience was 24.6 years (SD = 14.0).

### 2.3. Measures

Turnover intentions were measured with a 3-item Likert-type scale developed by Irving et al. [36]. Sample items and internal consistency estimates for this and the other scales presented below are provided in Supplementary Tables S1 and S2. We used principal component analysis, which confirmed the scale’s single-factor structure (the common factor explains 80.3% of the variance, and all items have loadings > 0.8).

Given the explanatory research design, we included a wide range of factors in the research questionnaire that may be relevant to nurses’ turnover intentions. Based on previous research (e.g., [4]), we measured six demographic factors (age, work experience, education, institution level, sector, and factual workload), as well as four work-related well-being factors (subjective health, workability, burnout and job satisfaction), eight job stressors (emotional job demands, physical job demands, cognitive job demands, workload, bureaucracy, bullying, interpersonal conflicts, role conflict), and 16 job resources (skill use, participation, availability of tools, satisfaction with the physical environment, reciprocity, pay, extra benefits, recognition, task variety, opportunities for development, support from the manager, support from colleagues, job control, adequacy of staffing, nursing administration skills, nurse-physician relationships). The instruments for these factors are detailed in Supplementary Table S1.

We used the 12-item Burnout Assessment Tool (BAT; [37]) to measure burnout. Respondents were asked to rate statements about their exhaustion, disengagement, cognitive and emotional difficulties on a 5-point Likert-type scale (1 = never, 5 = almost always). The reliability and validity of BAT in a Lithuanian sample have been confirmed in previous studies (e.g., [38]).

Several subscales of the Job Demands-Resources (JD-R) questionnaire [39] were used to assess work environment stressors and resources. Specifically, we assessed work stressors such as workload, emotional demands, physical demands, cognitive demands, red tape bureaucracy, role conflict, and interpersonal conflicts. We also assessed work resources, including skill use, task variety, participation, opportunities for development, managerial support, support from colleagues, recognition, work control, availability of tools, reciprocity, and pay. All of the items in these subscales were rated on a 5-point Likert-type scale from 1 (never/strongly disagree) to 5 (almost always/strongly agree). Workability was assessed by a single question from this scale, which was rated on a 10-point Likert-type scale. The reliability and validity of these measures in the Lithuanian sample were confirmed in previous studies (e.g., [40]).

We used three subscales from The Practice Environment Scale of the Nursing Work Index [41]. This instrument assessed Staffing and Resource Adequacy, Nurse Manager Ability, and Collegial Nurse–Physician Relations. Items were rated on a 4-point Likert-type scale ranging from 1 (strongly disagree) to 4 (strongly agree). The confirmatory factor analysis confirmed a three-factor structure of this scale (CFI = 0.990, TLI = 0.986, SRMR = 0.025, RMSEA = 0.041).

Subjective health was assessed by asking a single question (“How would you rate your overall physical health?”), which was rated on a 5-point scale (1—very poor, 5—very good). Job satisfaction was assessed by a single statement (“How satisfied are you overall with your job?”), asking to rate it on a scale from 0 (completely dissatisfied) to 5 (completely satisfied). Both of these questions are widely used in international surveys (e.g., [42,43]). Finally, based on focus group research conducted as part of the broader project, we developed single items to measure bullying, satisfaction with the physical environment, and the extra benefits provided by the employer.

The use of single-item measures is supported in the methodological literature, particularly when constructs are clearly defined and conceptually narrow. Evidence from large-scale validation studies shows that many organizational and psychological constructs can be reliably and validly assessed with single-item indicators. For example, Matthews et al. [44] demonstrated that the majority of single-item measures across a wide range of constructs showed adequate content validity and usability, and moderate to high test–retest reliability, with most demonstrating strong overall validity evidence. In addition, single-item measures can reduce respondent burden, survey length, and redundancy among items, which is particularly advantageous in large surveys or studies that include many constructs [44].

### 2.4. Data Analysis

Data analysis was conducted using an ANN-based regression model, which was developed in the Jupyter Lab programming environment with the Python 3.13 TensorFlow and Shap packages [32]. Following best practices in machine learning analysis [6,45], we split the dataset into 80% for training and 20% for testing. 30% of the training data were set aside as a validation subset during model fitting and were used to monitor model performance across epochs and guide training decisions via callbacks. As neural network architectures require complete input matrices for matrix multiplication and backpropagation, we employed list-wise deletion to handle missing data. Additionally, we used early stopping and learning rate reduction from the TensorFlow package, as well as L2 regularization on the weight matrix. We allowed the model to train up to 10,000 epochs. However, due to the early stopping callback (which stopped training the model if the validation MSE did not decrease for 10 consecutive epochs), the model was not trained for the full number of epochs. The regression weights of the model were optimized using gradient descent with the Adam (i.e., Adaptive Moment Estimation) optimizer [46].

Data analysis was conducted in three stages. First, we aimed to identify the most significant predictive factors for turnover intentions. To achieve this, a relatively basic ANN model was utilized. This model included one input and normalization layer, sixty-four neurons in the hidden layer with a ReLU activation function and L2 regularization (λ = 0.01), and a single neuron in the output layer without an activation function. We included all available predictors (k = 34) in the initial ANN regression model. We then applied both SHAP analysis and permutation feature importance to estimate the relative contributions of each predictor. Prognostic features were iteratively eliminated based on a combined importance criterion. At each iteration, variables were removed if they demonstrated both low mean absolute SHAP values and non-positive or near-zero permutation importance scores. In the initial stages, features with SHAP values ≤ 0.03–0.04 together with PIS ≤ 0 were excluded. In subsequent iterations, the criterion became more restrictive, and variables with SHAP values below approximately 0.04–0.05 and PIS < 0.01–0.02 were eliminated. This recursive feature elimination process continued until only variables with SHAP ≥ ~0.05 and PIS ≥ ~0.02 remained in the final model. Model performance improvement across iterations, as reflected by increasing R^2^ values, supported the adequacy of the applied thresholds. This iterative procedure allowed us to determine the optimal subset of predictors that maximized model performance.

In the second stage of the analysis, we optimized the ANN model’s hyperparameters. These hyperparameters included the number of hidden-layer neurons, the learning rate, the weight-matrix regularization coefficient (λ), and the batch size used to recalculate regression weights. We adjusted each hyperparameter individually, focusing on the coefficient of determination (R^2^) of the resulting model (in the test sample). We also evaluated other measures of the model’s predictive ability, specifically the mean absolute error (MAE, which indicates the average deviation of the true values from the forecast) and the root mean square error (RMSE, a more difficult-to-interpret measure that emphasizes large errors). To evaluate the predictive performance of the final ANN model in context, we created a linear regression model analogous to the ANN model using scikit-learn. To achieve model analogy, we split the data into training (80%) and test (20%) sets for the linear model and evaluated the model’s predictive accuracy (R^2^, RMSEA, MAE) on the test data.

In the third stage of the analysis, the optimized ANN model underwent SHAP analysis [34] using a testing sample. This analysis aimed to assess the importance of the predictor factors and the nature of their relationships with turnover intentions. We calculated the absolute SHAP values and examined the SHAP summary plot and dependence plots for each predictor variable. A positive SHAP value indicates that the factor positively predicts intentions to quit, while a negative SHAP value suggests a negative prediction. The absolute average SHAP value reflects the overall importance of each predictor variable in the model. To identify moderators that explain data variability, we evaluated interaction indices using the integrated function of the Python SHAP package, highlighting the values of the most likely moderator in the dependence plots.

## 3. Results

The (final) data analysis Python codes and outputs are provided in the Supplementary Materials (Codes S1 and S2). Means, standard deviations, and linear relationships (Pearson correlations) for all study variables are presented in Supplementary Table S2. Initially, we included all of the measured features in the basic ANN model. Based on low SHAP values and permutation importance scores, features were eliminated to retain the optimal number of predictors (see Table 1). After the first iteration, we eliminated the following: sector (private vs. public), recognition, red tape, workload, job control, nurse-physician relationships, tool availability, colleague support, physical demands, emotional demands, and satisfaction with the physical environment. Although the permutation importance scores for education, pay, and task variety were low (and negative), we decided to keep them in this stage, given their relatively high SHAP values. After the second iteration, we excluded task variety, work experience, skill use, factual workload, nurse manager ability, pay, workability, physical health, extra benefits, and participation. After the third iteration, we excluded the organization level and exposure to inappropriate workplace behavior. Finally, based on the results of the fourth iteration, we eliminated education level, cognitive demands, reciprocity, and staff adequacy. Therefore, the final model included seven prognostic features: age, job satisfaction, burnout, role conflict, interpersonal conflicts, development opportunities, and manager support.

In the second step of data analysis, we aimed to find the optimal hyperparameters of the model. To this end, we adjusted the number of neurons in the inner layer of the model (32, 64, 128, 256 and 512), the l2 regularization lambda (λ) coefficient (in the range from 0.005 to 0.1), the batch size (8, 16, 32, 64, 128, 256 and 512), and the learning rate (in the range from 0.01 to 0.00001). By adjusting one parameter at a time and recording the change in R2, we iteratively found that the model with 64 neurons in the inner layer predicted turnover intentions best when λ = 0.08, batch size = 64, and learning rate = 0.0001. The R^2^ of this model (in the testing sample) was 0.498 (MAE = 0.577, RMSE = 0.719).

To contextualize the predictive performance of the final ANN model, we also performed a linear regression analysis using the same predictive features. The results (output provided in Supplementary Material Code S2) showed that the predictive performance of the linear model was worse (R^2^ = 0.411, MAE = 0.624, RMSE = 0.770). Thus, the use of the ANN model to predict nurses’ turnover intentions seems justified.

Finally, we performed SHAP analysis of the optimized model to understand better the nature of the relationship between the model’s predictor variables and turnover intentions. To this end, we inspected the SHAP summary dot plot and dependence plots for each feature.

**Figure 1:**
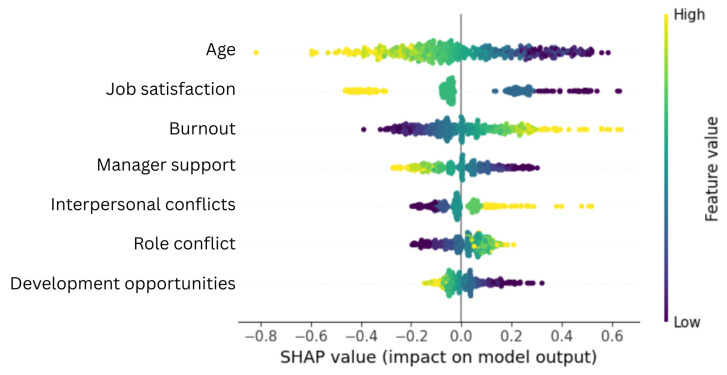
SHAP values for features in the final model.

As can be seen from the results (Figure 1), age was the most important predictor of turnover intentions in this model (mean absolute SHAP value = 0.210), and younger nurses were more likely to consider changing their jobs.

**Figure 2:**
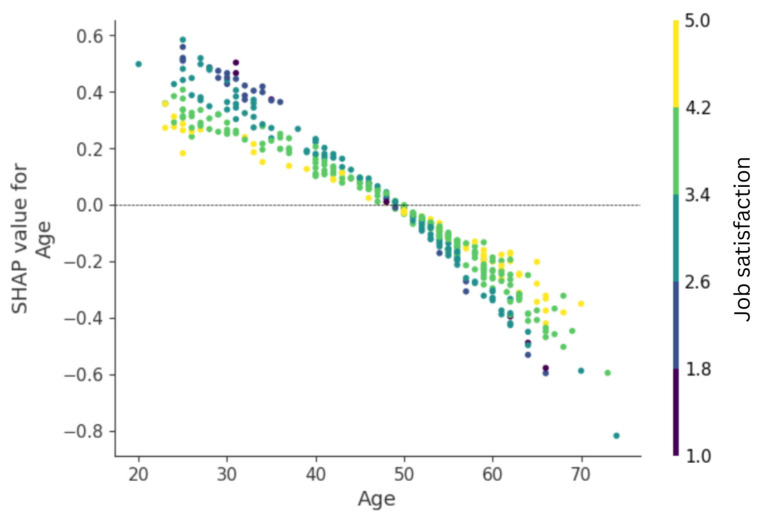
SHAP dependence plot for age.

The dependence plot for this feature (Figure 2) shows the SHAP values for each case in the test sample. In the SHAP dependence plots, each dot represents an individual study participant. The x-axis shows the actual value of a given predictor. In contrast, the y-axis shows the corresponding SHAP value, which reflects how much that predictor contributes to increasing or decreasing the predicted outcome (i.e., turnover intentions) relative to the model’s average prediction. Positive SHAP values indicate that the variable positively predicts turnover intentions, whereas negative values indicate a decreasing effect. The color gradient represents the value of a second interacting feature, allowing visualization of potential interaction effects. These plots therefore illustrate not only the direction and strength of association between a variable and the model prediction, but also potential non-linear relationships and interactions captured by the artificial neural network model.

There was considerable dispersion between SHAP values (especially among the youngest and the oldest nurses), which was most closely related to differences in job satisfaction. Higher job satisfaction was associated with lower turnover intentions among younger nurses. Paradoxically, with higher job satisfaction, older age was a weaker protective risk factor for turnover intentions.

**Figure 3:**
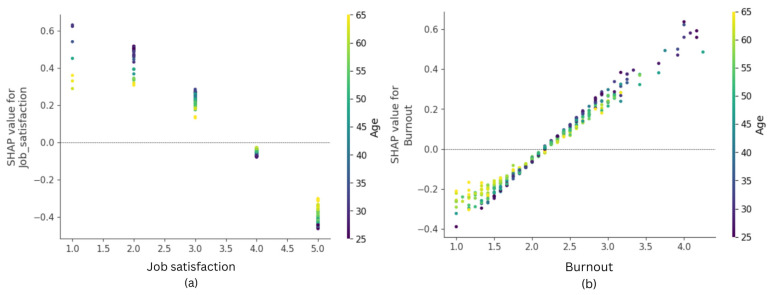
SHAP dependence plot for (**a**) job satisfaction and (**b**) burnout.

Two factors of work-related well-being—job satisfaction (mean absolute SHAP value = 0.188) and burnout (mean absolute SHAP value = 0.144)—were the second and third most important predictors in the model. As best seen in Figure 3, job satisfaction negatively and burnout positively predicted turnover intentions. Based on the interaction indices estimated by the Shap package, the dispersion of the SHAP scores for both factors was most strongly moderated by subjects’ age. Specifically, the predictive effect of high and low job satisfaction and burnout on turnover intentions was greater among younger nurses.

**Figure 4:**
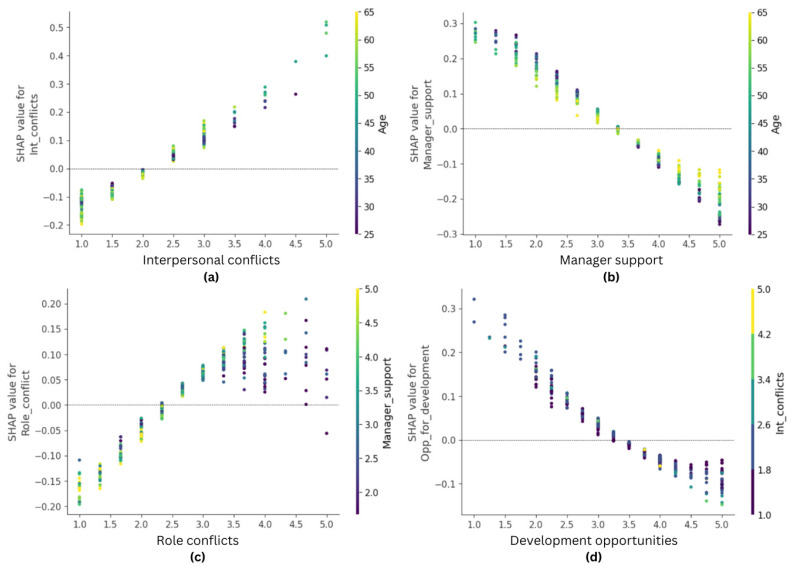
SHAP dependence plot for work environment features: (**a**) interpersonal conflicts, (**b**) Manager support, (**c**) role conflicts, (**d**) development opportunities.

Finally, four work environment factors emerged as important predictors of intention to quit, i.e., manager support (mean absolute SHAP value = 0.111), interpersonal conflicts (mean absolute SHAP value = 0.077), role conflict (mean absolute SHAP value = 0.070), and development opportunities (mean absolute SHAP value = 0.063). Although one might expect that job stressors would predict turnover intentions positively and resources negatively, the SHAP dependency plots (Figure 4) presented a more nuanced picture. Managerial support was clearly a protective factor; SHAP values were positive when nurses felt a lack of managerial support and negative when they felt supported by their managers. Interestingly, this effect was somewhat related to the nurses’ age and was more pronounced among younger nurses. Similarly, interpersonal conflict appears to be a clear risk factor, gaining positive SHAP values on the right-hand side of the graph. Although age best explains the distribution of interpersonal conflict SHAP values according to the interaction indices, the colored part of the graph does not seem informative.

The SHAP dependence plot for role conflict indicates a predominantly monotonic increase in predicted turnover intentions as role conflict intensifies. SHAP values shift from clearly negative at low levels (1–2) to increasingly positive values at higher levels (4–5), crossing the zero line around the midpoint of the scale. Importantly, the magnitude of positive SHAP values at high role conflict is greater than that of negative values at low role conflict, suggesting an asymmetric effect: high role conflict contributes more strongly to increasing turnover intentions than low role conflict does to reducing them. Although the overall pattern appears close to linear, some deviation from strict linearity is evident at the upper end of the scale, where the slope steepens, and the dispersion of SHAP values increases. This wider spread at high levels of role conflict likely reflects interaction effects captured by the ANN model, indicating that the impact of role conflict depends on contextual factors (e.g., manager support).

The dependence plot for development opportunities demonstrates a clear negative association with turnover intentions, with SHAP values decreasing as development opportunities increase. At low levels (1–2), SHAP values are distinctly positive, indicating that insufficient opportunities substantially elevate predicted turnover intentions. Around the mid-range (≈3), the effect approaches neutrality, whereas higher levels (4–5) are associated with negative SHAP values, reflecting a protective effect. The relationship is not perfectly linear: the decline in SHAP values appears steeper between low and moderate levels. It flattens somewhat at the highest levels, suggesting diminishing marginal returns of development opportunities once a certain threshold is reached. Additionally, some dispersion of SHAP values across the scale indicates that the strength of this protective effect varies between individuals, likely due to interactions with other work environment characteristics (e.g., interpersonal conflict). Overall, the plot suggests that the absence of development opportunities is more consequential than their incremental improvement beyond moderate levels, underscoring a threshold-like dynamic rather than a strictly linear relationship.

## 4. Discussion

This study uniquely applies artificial neural networks to identify demographic, well-being, and work-environment predictors of nurses’ turnover intentions, using a comprehensive set of influencing factors. The inclusion of a broad range of factors offers clear practical benefits by enabling a thorough assessment of the factors that contribute to turnover. However, the large number of factors poses a methodological challenge for machine learning models, commonly known as the “curse of dimensionality” [47]. As the number of predictive factors increases, the reliability of machine learning models may decrease due to the exponential growth in sparsity between data points. To address this issue, we performed dimensionality reduction via feature selection, retaining only the most significant predictive features in our ANN model.

Using SHAP and permutation importance analysis, we reduced the number of predictive factors in our model to the seven most significant ones. Among these, the age of the research participants had the highest predictive value. Specifically, older nurses were less likely than their younger counterparts to consider changing jobs. This finding aligns with previous studies indicating that younger nurses are more inclined to consider changing their workplaces [20,27]. Our results also indicated that the relationship between age and turnover intentions is somewhat nonlinear and heteroskedastic. In general, the likelihood of a nurse considering leaving their job decreases across the entire age range; however, this decline accelerates with increasing age. Furthermore, SHAP values were more widely dispersed at both ends of the age spectrum and less concentrated in the middle. Conceptually, this suggests that the predictive effect of age may vary between the youngest and oldest nurses and could be influenced by specific circumstances.

Two well-being indicators—job satisfaction and burnout—were among the most significant predictors of turnover intentions. As expected, nurses were more likely to consider changing jobs when they experienced higher levels of burnout and lower job satisfaction. These findings align with previous research indicating a connection between employee well-being and turnover intentions [15,16,17]. Specifically, nurses tend to leave organizations that do not prioritize their work-related well-being. Additionally, our results suggest a potential interaction between these well-being indicators and age. Specifically, low job satisfaction and burnout had higher SHAP values (i.e., they were stronger predictors of turnover intentions) among younger nurses. This might imply that younger nurses were more likely to consider their work-related well-being when deciding whether to change jobs than their older counterparts. We will return to this insight when discussing the study’s practical implications.

In the final artificial neural network (ANN) model, four work environment characteristics emerged as significant factors influencing turnover intentions. The overall predictive effects aligned with expectations: stressors positively predicted turnover intentions, while resources negatively predicted turnover intentions [16,17,48]. However, the chosen data analysis strategy revealed interesting and practically relevant patterns for some of these factors. Manager support emerged as a crucial protective factor, with a relatively linear, homoscedastic predictive relationship. This finding aligns with previous research indicating that high levels of managerial support buffer against various work stressors, whereas low social support from managers tends to encourage job changes [20]. Similarly, interpersonal conflicts were identified as a clear risk factor. Nurses who reported experiencing higher levels of conflicts were more likely to consider changing jobs. This supports earlier research suggesting that unresolved interpersonal problems can lead nurses to seek employment elsewhere [18,19]. Our study further reinforces these findings.

A more theoretically and practically interesting pattern emerged when examining the predictive effect of development opportunities. Previous research consistently shows that access to career advancement, training, and professional growth opportunities is associated with lower turnover intentions both in the general employee population [49] and among nurses [50]. However, the present results suggest an asymmetric relationship. When development opportunities are high, SHAP values remain only slightly negative and relatively dispersed, indicating a limited protective effect. In contrast, when opportunities are scarce, the predicted contribution to turnover intentions becomes strongly positive. This pattern implies that development opportunities function less as a strong motivational driver and more as a basic organizational resource whose absence becomes particularly consequential.

From a theoretical perspective, this finding aligns with the Job Demands–Resources (JD-R; [22]) framework, which posits that the absence of resources may act as a strain-inducing condition rather than the presence of resources acting as a strong motivator. Once a minimum acceptable level of professional development is available, additional opportunities may yield diminishing returns in terms of reducing turnover intentions. In the nursing context, insufficient opportunities for professional growth may signal career stagnation and limited long-term prospects, thereby increasing the likelihood that employees consider alternative employment. Consequently, the findings suggest that ensuring at least a baseline level of development opportunities may be more critical for retention than continuously expanding such opportunities beyond moderate levels.

Intriguing patterns also emerged regarding the predictive effect of role conflict. Consistent with prior research, role conflict generally acted as a job demand associated with stronger turnover intentions [17,51]. The SHAP dependence plot indicates that low levels of role conflict consistently reduce predicted turnover intentions, suggesting that clearly structured and compatible role expectations function as a protective factor. As role conflict increases, its contribution becomes increasingly positive, indicating that incompatible demands and expectations may strengthen withdrawal cognitions. However, the effect becomes substantially more variable at higher levels of role conflict, as reflected in the wider dispersion of SHAP values. This heterogeneity suggests that the impact of role conflict is likely contingent on contextual resources, consistent with JD-R theory, which emphasizes the interaction between job demands and job resources [22]. In the present model, role conflict showed the strongest interaction with manager support, suggesting that managerial guidance may partially buffer the negative consequences of role conflict. Although the dependence plot does not allow a precise interpretation of the interaction direction, the pattern suggests that supportive leadership may help employees manage incompatible demands. In contrast, unresolved role conflict in low-support environments may more strongly contribute to turnover intentions.

The seven-factor artificial neural network (ANN) model explained nearly half (49.8%) of the variance in turnover intentions. While this is not perfect, it outperforms previous studies that utilized linear regression models [52]. Similarly, in this study, the linear regression model explained only 41.1% of the variance in nurses’ turnover intentions. This suggests that the ANN’s ability to reveal nonlinear and heteroscedastic relationships, along with its lower sensitivity to multicollinearity, yields better predictions of nurses’ career decisions.

In this study, turnover intentions were conceptualized as a continuous rather than a dichotomous variable. This approach sets our study apart from others that have used machine learning methods to categorize nurses into those with and without intentions to leave their jobs [12,13,27]. Binary clustering makes results easier to interpret and may be beneficial for organizations assessing potential employee turnover, but treating intentions as a continuum retains valuable information about their strength. This is crucial for understanding the factors that drive workplace change and for implementing more targeted preventive measures.

### 4.1. Limitations and Future Research

When interpreting the results, several important limitations of the study should be considered.

First, although the sample included a relatively large number of nurses from different healthcare sectors, it used non-probabilistic convenience sampling, which increases the risk of self-selection bias. Participation was voluntary, and nurses who were more professionally engaged or motivated may have been overrepresented. In contrast, those experiencing extreme workload, burnout, or strong intentions to leave may have been underrepresented. Because no probability-based sampling was used, the representativeness of the sample relative to the national population of nurses in Lithuania cannot be fully established, and the distribution of demographic and occupational characteristics may not accurately reflect national workforce statistics. Although the achieved sample size was sufficient for training and validating the artificial neural network model and can be considered adequate for predictive modeling within the Lithuanian context, external validity is somewhat limited by the sampling strategy.

In addition, the study sample was geographically limited to a single country. Historically, Lithuanian nursing was shaped by Soviet-style vocational training with limited academic grounding until reforms began around 1990, in contrast to many Western European countries, where nursing education has been university-based for longer [53]. Overall, Lithuania’s nursing profession is rapidly evolving but still faces challenges related to modernizing education, developing cultural competence, and managing stress compared to other European nations [54]. On the other hand, research shows that, like their European counterparts, Lithuanian nurses face role ambiguity, limited recognition, and underused competencies [29]. Therefore, generalizing the results to the European context is possible but should be done with caution.

Second, the study relied exclusively on self-report measures to assess all key variables. While self-reports are widely used and appropriate for capturing subjective experiences, they may be affected by response bias. Future research would benefit from incorporating objective or observational data, such as assessments of work conditions or comparisons between reported turnover intentions and actual turnover behavior.

Third, despite the large sample size and broad range of assessed variables, the data were collected using a cross-sectional design. This limits conclusions about causality and the development of turnover intentions over time. Longitudinal designs would be particularly valuable for future machine learning research, allowing for richer data across individuals, variables, and time points.

Fourth, to optimize model performance, feature selection was applied, which may have reduced the visibility of some practically important factors. Certain variables may have been overshadowed by strong correlations or mediating effects, such as the role of nursing administrators’ abilities or the mediating influence of job satisfaction and burnout.

Fifth, in this study, we focused solely on factors related to the current job. However, it is important to recognize that when considering a job change, the conditions of the current workplace are not the only factors at play; the perceived opportunities for better conditions in other workplaces also matter. Therefore, future research should analyze not only the push and pull factors of the current job but also the perceived opportunities available in the labor market.

### 4.2. Practical Implications for Healthcare Management

Burnout and low job satisfaction are among the strongest indicators that nurses are considering leaving their jobs, highlighting the need for organizations to monitor these factors and implement preventive measures actively. Promoting employee well-being and ensuring sustainable staff retention should therefore be viewed as interconnected goals essential to delivering high-quality healthcare [17]. Research consistently shows that threats to employee well-being are often rooted in structural and organizational conditions rather than individual shortcomings [55]. As a result, healthcare organizations should prioritize improving the work environment by reducing job-related stressors and ensuring adequate resources, instead of focusing solely on individual-level interventions.

Nurses’ turnover intentions are also strongly influenced by the quality of managerial support and the organization’s ability to prevent and resolve workplace conflicts. Investing in the development of managerial competencies—particularly in leadership, communication, and conflict management—can substantially reduce nurses’ intentions to leave [17]. It is therefore important to critically review healthcare career pathways to ensure that individuals in managerial roles possess strong interpersonal and supervisory skills. Poor communication, frequent conflicts, unclear work expectations, and insufficient managerial support increase stress and job dissatisfaction among nurses [18,19,20], whereas supportive leadership contributes to a healthier organizational climate and improved care quality [55].

Finally, clear role definitions and opportunities for professional development play a key role in preventing turnover intentions. Nurses who feel valued, have well-defined responsibilities, and see realistic prospects for growth are less likely to seek employment elsewhere. Creating transparent career pathways and protecting nurses from role ambiguity can therefore strengthen retention.

## 5. Conclusions

In conclusion, this study demonstrates that applying artificial neural networks enables more accurate prediction of nurses’ turnover intentions and reveals complex, nonlinear relationships among demographic, well-being, and work environment factors. By identifying seven key predictors, the findings highlight the central role of age, burnout, job satisfaction, and managerial support, while also showing that certain factors—such as development opportunities and role conflict—become particularly influential when they are lacking or when their effects depend on other workplace conditions. Moreover, conceptualizing turnover intentions as a continuous rather than a dichotomous outcome provided a more nuanced understanding of the strength of nurses’ intentions to leave, which is often lost in binary classifications.

These findings have important implications for nurse retention policies and organizational strategies within healthcare systems. The results indicate that retention efforts should focus on modifiable organizational factors. Specifically, it is essential to strengthen managerial support, reduce burnout, and enhance job satisfaction by improving workload management, clarifying roles, and increasing access to professional development. Instead of relying solely on individual-level interventions, healthcare organizations and policymakers should adopt systemic approaches that emphasize effective leadership practices, supportive work environments, and sustainable workforce planning. Investing in these areas may not only reduce turnover intentions but also lessen the likelihood of high-intensity intentions to leave among vulnerable staff groups.

## Figures and Tables

**Table 1:** Prognostic features’ SHAP values and permutation importance scores.

Prognostic Feature	Iteration									
	I		II		III		IV		V	
	SHAP	PIS	SHAP	PIS	SHAP	PIS	SHAP	PIS	SHAP	PIS
Level	0.058	0.019	0.046	0.015	*0.054*	*−0.014*				
Sector	*0.012*	*−0.001*								
Age	0.097	0.047	0.168	0.086	0.141	0.088	0.207	0.123	0.216	0.179
Education	0.059	−0.009	0.052	0.009	0.058	0.028	*0.070*	*0.011*		
Experience	0.072	0.014	*0.025*	*−0.009*						
Factual workload	0.002	0.003	*0.001*	*0.003*						
Physical health	0.039	0.008	*0.045*	*0.007*						
Workability	0.033	0.004	*0.042*	*0.006*						
Job satisfaction	0.183	0.102	0.166	0.084	0.185	0.120	0.204	0.149	0.199	0.177
Burnout	0.138	0.061	0.164	0.108	0.138	0.079	0.134	0.077	0.150	0.068
Emotional demands	*0.036*	*−0.016*								
Physical demands	*0.036*	*−0.015*								
Cognitive demands	0.039	0.006	0.038	0.012	0.035	0.020	*0.038*	*0.009*		
IWB	0.059	0.005	0.064	0.014	*0.051*	*0.008*				
Workload	*0.029*	*−0.002*								
Red tape	*0.026*	*−0.001*								
Role conflict	0.081	0.013	0.049	0.032	0.048	0.012	0.069	0.031	0.086	0.033
Interpersonal conflicts	0.041	0.015	0.046	0.010	0.070	0.007	0.059	0.019	0.078	0.033
Skill use	0.032	0.012	*0.033*	*0.002*						
Participation	0.033	0.019	*0.032*	*0.011*						
Tool availability	*0.038*	*−0.012*								
SPE	*0.035*	*−0.020*								
Reciprocity	0.046	0.021	0.072	0.033	0.065	0.019	*0.054*	*0.006*		
Pay	0.064	−0.001	*0.037*	*0.005*						
Extra benefits	0.041	0.003	*0.032*	*0.009*						
recognition	*0.033*	*−0.001*								
Task variety	0.050	−0.019	*0.030*	*−0.012*						
Development opportunities	0.089	0.018	0.053	0.013	0.057	0.020	0.067	0.038	0.063	0.018
Manager support	0.032	0.005	0.112	0.045	0.104	0.053	0.100	0.021	0.116	0.030
Colleague support	*0.029*	*−0.012*								
Job control	*0.034*	*−0.008*								
Staff adequacy	0.048	0.004	0.040	0.013	0.040	0.013	*0.025*	*0.007*		
Nursing manager ability	0.055	0.014	*0.034*	*0.005*						
NPR	*0.033*	*−0.010*								
R^2^	0.258		0.340		0.454		0.420		0.481	

SHAP—mean absolute SHAP value, PIS—permutation importance score, IWB—inappropriate workplace behavior, SPE—Satisfaction with physical environment, NPR—nurse-physician relationships. Values for least important features are presented in italics.

## Data Availability

The dataset used and analyzed during the current study is available from the corresponding author on reasonable request. The data are not publicly available due to privacy restrictions.

## Supplementary Materials

The following supporting information can be downloaded at https://www.mdpi.com/article/10.3390/healthcare14070831/s1: Table S1: Study measures and sample items; Table S2: Descriptive statistics and correlations between study variables; Code S1: Python code and output for the final ANN data analysis; Code S2: Python code and output for the linear regression model; TRIPOD Checklist: Prediction Model Development and Validation.

## Abbreviations

The following abbreviations are used in this manuscript:

ANN	Artificial Neural Network
ML	Machine Learning
PIS	Permutation Importance Score
SHAP	SHapley Additive exPlanations
