# Supplementary material for: Exploring the Predictors of Nurses’ Turnover Intentions Through Neural Network Modeling: A National Cross-Sectional Study in Lithuania
Source: Healthcare (Basel). 2026 Mar 24;14(7):831. doi: 10.3390/healthcare14070831 (PMC13073792; doi:10.3390/healthcare14070831)
Supplement: Supplementary file 1 [file healthcare-14-00831-s001.zip › Code S1.html]

ML\_regresija


### Code S1.

*Python code and output for final data analysis.*

In [1]:

```
#importing

import os
seed = 1
os.environ['PYTHONHASHSEED'] = str(seed)
os.environ['TF_DETERMINISTIC_OPS'] = '1'        # force TF deterministic ops where possible
os.environ['TF_CUDNN_DETERMINISTIC'] = '1'     # force deterministic cuDNN algorithm selection
# (rekomenduojama - bet nebūtina) os.environ['TF_CPP_MIN_LOG_LEVEL'] = '2'

import random
import matplotlib.pyplot as plt
import numpy as np
import pandas as pd
import seaborn as sns
from sklearn.metrics import r2_score

# Make NumPy printouts easier to read.
np.set_printoptions(precision=3, suppress=True)

import shap
import tensorflow as tf
from tensorflow import keras
from tensorflow.keras import layers
from tensorflow.keras import regularizers
from tensorflow.keras.callbacks import EarlyStopping, ReduceLROnPlateau, ModelCheckpoint
from tensorflow.keras.layers import Dense, Dropout, BatchNormalization

random.seed(seed)
np.random.seed(seed)
tf.random.set_seed(seed)
keras.utils.set_random_seed(seed)

tf.config.threading.set_intra_op_parallelism_threads(1)
tf.config.threading.set_inter_op_parallelism_threads(1)
tf.keras.backend.clear_session()
tf.config.experimental.enable_op_determinism() #turėtų panaikinti modelių skirtumus

print("tf versija:", tf.__version__)
```

```
C:\Users\azied\AppData\Local\Programs\Python\Python313\Lib\site-packages\tqdm\auto.py:21: TqdmWarning: IProgress not found. Please update jupyter and ipywidgets. See https://ipywidgets.readthedocs.io/en/stable/user_install.html
  from .autonotebook import tqdm as notebook_tqdm
```

```
WARNING:tensorflow:From C:\Users\azied\AppData\Local\Programs\Python\Python313\Lib\site-packages\keras\src\backend\common\global_state.py:82: The name tf.reset_default_graph is deprecated. Please use tf.compat.v1.reset_default_graph instead.

tf versija: 2.20.0
```

In [2]:

```
#datafile and variables

DataFile = "C:\\Users\\azied\\Desktop\\Slaugytojų_2025_duomenys\\Nurse_ML(recode).csv"
IndVars = ["amzius",
           "Pdarbu", "Burnout",
           "Vkonflikt", "Konfliktai",
           "Tobul","Vparama"]
DepVar = "Turnover"

TotVars = IndVars
TotVars.append(DepVar)

raw_dataset = pd.read_csv(DataFile)
for col in raw_dataset.columns:
    raw_dataset[col] = pd.to_numeric(raw_dataset[col], errors='coerce')  #konvertuoja į skaičius (kitkas - NaN)
dataset = raw_dataset[TotVars].copy()
dataset = dataset.sample(frac=1).reset_index(drop=True)
dataset.tail()
```

Out[2]:

|  | amzius | Pdarbu | Burnout | Vkonflikt | Konfliktai | Tobul | Vparama | Turnover |
| --- | --- | --- | --- | --- | --- | --- | --- | --- |
| 3773 | 28.0 | 4.0 | 2.916667 | 3.000000 | 2.5 | 3.75 | NaN | 2.666667 |
| 3774 | 60.0 | 3.0 | 2.666667 | 3.333333 | 2.0 | 3.50 | 2.0 | 2.666667 |
| 3775 | NaN | NaN | NaN | 2.666667 | NaN | 3.50 | NaN | NaN |
| 3776 | NaN | NaN | NaN | NaN | NaN | NaN | NaN | NaN |
| 3777 | NaN | NaN | NaN | NaN | NaN | NaN | NaN | NaN |

In [3]:

```
#droping missing values
dataset = dataset.dropna()
print("Pašalintos praleistos reikšmės")
print("Likusi bendra imtis:", len(dataset))
```

```
Pašalintos praleistos reikšmės
Likusi bendra imtis: 2129
```

In [4]:

```
#separating training and test datasets

train_dataset = dataset.sample(frac=0.8, random_state=0)
test_dataset = dataset.drop(train_dataset.index)

train_features = train_dataset.copy()
test_features = test_dataset.copy()

train_labels = train_features.pop(DepVar)
test_labels = test_features.pop(DepVar)
```

In [5]:

```
#Building normalizer

normalizer = tf.keras.layers.Normalization(axis=-1)
normalizer.adapt(np.array(train_features))
```

In [6]:

```
# Callbacks
callbacks = [
    EarlyStopping(
        monitor='val_loss',
        patience=10,
        restore_best_weights=True,
        start_from_epoch=1
    ),
    
    # Mažina learning rate, jei modelis stringa
    ReduceLROnPlateau(
        monitor='val_loss',
        factor=0.5,           # sumažina learning rate per pusę
        patience=10,           # kiek epochų laukti prieš sumažinant
        min_lr=1e-8           # minimalus learning rate
    ),
]
```

In [7]:

```
# Model creation
model = tf.keras.Sequential([
    tf.keras.Input(shape=(train_features.shape[1],)),
    normalizer,                      # 1. Normalizacija
    layers.Dense(64, 
                 kernel_regularizer=regularizers.l2(0.08), 
                 activation='relu'),  # 2. Pirmas paslėptas sluoksnis
    #layers.Dropout(0.1),
    #BatchNormalization(),
    layers.Dense(1)                       # 4. Išvesties sluoksnis (vienas neuroninis, be aktyvacijos)
])
```

In [8]:

```
#model compiling
model.compile(loss='mean_squared_error',
                optimizer=tf.keras.optimizers.Adam(0.0001))

model.summary()
```

```
Model: "sequential"
```

```
┏━━━━━━━━━━━━━━━━━━━━━━━━━━━━━━━━━━━━━━┳━━━━━━━━━━━━━━━━━━━━━━━━━━━━━┳━━━━━━━━━━━━━━━━━┓
┃ Layer (type)                         ┃ Output Shape                ┃         Param # ┃
┡━━━━━━━━━━━━━━━━━━━━━━━━━━━━━━━━━━━━━━╇━━━━━━━━━━━━━━━━━━━━━━━━━━━━━╇━━━━━━━━━━━━━━━━━┩
│ normalization (Normalization)        │ (None, 7)                   │              15 │
├──────────────────────────────────────┼─────────────────────────────┼─────────────────┤
│ dense (Dense)                        │ (None, 64)                  │             512 │
├──────────────────────────────────────┼─────────────────────────────┼─────────────────┤
│ dense_1 (Dense)                      │ (None, 1)                   │              65 │
└──────────────────────────────────────┴─────────────────────────────┴─────────────────┘
```

```
 Total params: 592 (2.32 KB)
```

```
 Trainable params: 577 (2.25 KB)
```

```
 Non-trainable params: 15 (64.00 B)
```

In [9]:

```
#loss plot function
def plot_loss(history):
  plt.plot(history.history['loss'], label='loss')
  plt.plot(history.history['val_loss'], label='val_loss')
  plt.ylim([0, 10])
  plt.xlabel('Epoch')
  plt.ylabel('Error')
  plt.legend()
  plt.grid(True)
```

In [10]:

```
#modelio training function.
#returns r-sq and training history
def model_fit(model):
    history = model.fit(
        train_features,
        train_labels,
        validation_split=0.3,
        verbose=0, epochs=10000,
        batch_size=64,
        callbacks=callbacks,
        shuffle=False)
    X_input = tf.convert_to_tensor(test_features.to_numpy(), dtype=tf.float32)
    y_pred = model(X_input, training=False).numpy()
    r2 = r2_score(test_labels, y_pred)
    return(r2, history)
```

In [11]:

```
#printing r-sq and training (loss) function.
r2, history = model_fit(model)
print("Rsq =", r2)
plot_loss(history)
```

```
Rsq = 0.49792356143733996
```

In [13]:

```
#SHAP explainer
background = train_features.to_numpy()
explainer = shap.Explainer(model.predict, background)
print(explainer)
```

```
<shap.explainers._exact.ExactExplainer object at 0x0000018E34964D70>
```

In [14]:

```
#calculating SHAP values
shap_values = explainer(test_features)
```

```
363/363 ━━━━━━━━━━━━━━━━━━━━ 0s 630us/step
352/352 ━━━━━━━━━━━━━━━━━━━━ 0s 537us/step
25/25 ━━━━━━━━━━━━━━━━━━━━ 0s 926us/step
338/338 ━━━━━━━━━━━━━━━━━━━━ 0s 444us/step
25/25 ━━━━━━━━━━━━━━━━━━━━ 0s 909us/step
354/354 ━━━━━━━━━━━━━━━━━━━━ 0s 464us/step
25/25 ━━━━━━━━━━━━━━━━━━━━ 0s 948us/step
357/357 ━━━━━━━━━━━━━━━━━━━━ 0s 448us/step
25/25 ━━━━━━━━━━━━━━━━━━━━ 0s 1ms/step 
367/367 ━━━━━━━━━━━━━━━━━━━━ 0s 482us/step
25/25 ━━━━━━━━━━━━━━━━━━━━ 0s 1ms/step 
365/365 ━━━━━━━━━━━━━━━━━━━━ 0s 481us/step
25/25 ━━━━━━━━━━━━━━━━━━━━ 0s 1ms/step 
350/350 ━━━━━━━━━━━━━━━━━━━━ 0s 547us/step
25/25 ━━━━━━━━━━━━━━━━━━━━ 0s 865us/step
377/377 ━━━━━━━━━━━━━━━━━━━━ 0s 470us/step
25/25 ━━━━━━━━━━━━━━━━━━━━ 0s 1ms/step
```

```
ExactExplainer explainer:   2%|█▎                                                              | 9/426 [00:00<?, ?it/s]
```

```
371/371 ━━━━━━━━━━━━━━━━━━━━ 0s 469us/step
25/25 ━━━━━━━━━━━━━━━━━━━━ 0s 938us/step
```

```
ExactExplainer explainer:   3%|█▍                                                     | 11/426 [00:10<01:21,  5.10it/s]
```

```
358/358 ━━━━━━━━━━━━━━━━━━━━ 0s 493us/step
25/25 ━━━━━━━━━━━━━━━━━━━━ 0s 986us/step
```

```
ExactExplainer explainer:   3%|█▌                                                     | 12/426 [00:10<01:50,  3.76it/s]
```

```
361/361 ━━━━━━━━━━━━━━━━━━━━ 0s 451us/step
25/25 ━━━━━━━━━━━━━━━━━━━━ 0s 2ms/step
```

```
ExactExplainer explainer:   3%|█▋                                                     | 13/426 [00:11<02:11,  3.15it/s]
```

```
380/380 ━━━━━━━━━━━━━━━━━━━━ 0s 610us/step
25/25 ━━━━━━━━━━━━━━━━━━━━ 0s 1ms/step
```

```
ExactExplainer explainer:   3%|█▊                                                     | 14/426 [00:11<02:36,  2.63it/s]
```

```
376/376 ━━━━━━━━━━━━━━━━━━━━ 0s 476us/step
25/25 ━━━━━━━━━━━━━━━━━━━━ 0s 1ms/step
```

```
ExactExplainer explainer:   4%|█▉                                                     | 15/426 [00:12<02:43,  2.52it/s]
```

```
338/338 ━━━━━━━━━━━━━━━━━━━━ 0s 493us/step
25/25 ━━━━━━━━━━━━━━━━━━━━ 0s 1ms/step
```

```
ExactExplainer explainer:   4%|██                                                     | 16/426 [00:12<02:57,  2.31it/s]
```

```
342/342 ━━━━━━━━━━━━━━━━━━━━ 0s 485us/step
25/25 ━━━━━━━━━━━━━━━━━━━━ 0s 976us/step
```

```
ExactExplainer explainer:   4%|██▏                                                    | 17/426 [00:13<02:55,  2.33it/s]
```

```
370/370 ━━━━━━━━━━━━━━━━━━━━ 0s 447us/step
25/25 ━━━━━━━━━━━━━━━━━━━━ 0s 898us/step
```

```
ExactExplainer explainer:   4%|██▎                                                    | 18/426 [00:13<02:47,  2.43it/s]
```

```
349/349 ━━━━━━━━━━━━━━━━━━━━ 0s 455us/step
25/25 ━━━━━━━━━━━━━━━━━━━━ 0s 1ms/step
```

```
ExactExplainer explainer:   4%|██▍                                                    | 19/426 [00:13<02:42,  2.50it/s]
```

```
342/342 ━━━━━━━━━━━━━━━━━━━━ 0s 448us/step
25/25 ━━━━━━━━━━━━━━━━━━━━ 0s 892us/step
```

```
ExactExplainer explainer:   5%|██▌                                                    | 20/426 [00:14<02:51,  2.36it/s]
```

```
366/366 ━━━━━━━━━━━━━━━━━━━━ 0s 495us/step
25/25 ━━━━━━━━━━━━━━━━━━━━ 0s 1ms/step
```

```
ExactExplainer explainer:   5%|██▋                                                    | 21/426 [00:14<03:00,  2.24it/s]
```

```
357/357 ━━━━━━━━━━━━━━━━━━━━ 0s 439us/step
25/25 ━━━━━━━━━━━━━━━━━━━━ 0s 1ms/step
```

```
ExactExplainer explainer:   5%|██▊                                                    | 22/426 [00:15<02:53,  2.33it/s]
```

```
348/348 ━━━━━━━━━━━━━━━━━━━━ 0s 553us/step
25/25 ━━━━━━━━━━━━━━━━━━━━ 0s 1ms/step
```

```
ExactExplainer explainer:   5%|██▉                                                    | 23/426 [00:15<02:55,  2.29it/s]
```

```
357/357 ━━━━━━━━━━━━━━━━━━━━ 0s 509us/step
25/25 ━━━━━━━━━━━━━━━━━━━━ 0s 1ms/step
```

```
ExactExplainer explainer:   6%|███                                                    | 24/426 [00:16<02:53,  2.32it/s]
```

```
364/364 ━━━━━━━━━━━━━━━━━━━━ 0s 480us/step
25/25 ━━━━━━━━━━━━━━━━━━━━ 0s 1ms/step
```

```
ExactExplainer explainer:   6%|███▏                                                   | 25/426 [00:16<02:54,  2.30it/s]
```

```
359/359 ━━━━━━━━━━━━━━━━━━━━ 0s 441us/step
25/25 ━━━━━━━━━━━━━━━━━━━━ 0s 1ms/step
```

```
ExactExplainer explainer:   6%|███▎                                                   | 26/426 [00:16<02:50,  2.35it/s]
```

```
352/352 ━━━━━━━━━━━━━━━━━━━━ 0s 451us/step
25/25 ━━━━━━━━━━━━━━━━━━━━ 0s 1ms/step
```

```
ExactExplainer explainer:   6%|███▍                                                   | 27/426 [00:17<02:37,  2.54it/s]
```

```
345/345 ━━━━━━━━━━━━━━━━━━━━ 0s 551us/step
25/25 ━━━━━━━━━━━━━━━━━━━━ 0s 1ms/step
```

```
ExactExplainer explainer:   7%|███▌                                                   | 28/426 [00:17<02:35,  2.57it/s]
```

```
342/342 ━━━━━━━━━━━━━━━━━━━━ 0s 464us/step
25/25 ━━━━━━━━━━━━━━━━━━━━ 0s 1ms/step
```

```
ExactExplainer explainer:   7%|███▋                                                   | 29/426 [00:18<02:33,  2.58it/s]
```

```
360/360 ━━━━━━━━━━━━━━━━━━━━ 0s 448us/step
25/25 ━━━━━━━━━━━━━━━━━━━━ 0s 1ms/step
```

```
ExactExplainer explainer:   7%|███▊                                                   | 30/426 [00:18<02:47,  2.36it/s]
```

```
364/364 ━━━━━━━━━━━━━━━━━━━━ 0s 499us/step
25/25 ━━━━━━━━━━━━━━━━━━━━ 0s 1ms/step
```

```
ExactExplainer explainer:   7%|████                                                   | 31/426 [00:18<02:42,  2.43it/s]
```

```
354/354 ━━━━━━━━━━━━━━━━━━━━ 0s 477us/step
25/25 ━━━━━━━━━━━━━━━━━━━━ 0s 955us/step
```

```
ExactExplainer explainer:   8%|████▏                                                  | 32/426 [00:19<02:39,  2.47it/s]
```

```
363/363 ━━━━━━━━━━━━━━━━━━━━ 0s 457us/step
25/25 ━━━━━━━━━━━━━━━━━━━━ 0s 2ms/step
```

```
ExactExplainer explainer:   8%|████▎                                                  | 33/426 [00:19<02:35,  2.53it/s]
```

```
347/347 ━━━━━━━━━━━━━━━━━━━━ 0s 540us/step
25/25 ━━━━━━━━━━━━━━━━━━━━ 0s 934us/step
```

```
ExactExplainer explainer:   8%|████▍                                                  | 34/426 [00:20<02:36,  2.50it/s]
```

```
374/374 ━━━━━━━━━━━━━━━━━━━━ 0s 453us/step
25/25 ━━━━━━━━━━━━━━━━━━━━ 0s 1ms/step
```

```
ExactExplainer explainer:   8%|████▌                                                  | 35/426 [00:20<02:38,  2.47it/s]
```

```
364/364 ━━━━━━━━━━━━━━━━━━━━ 0s 549us/step
25/25 ━━━━━━━━━━━━━━━━━━━━ 0s 1ms/step
```

```
ExactExplainer explainer:   8%|████▋                                                  | 36/426 [00:20<02:39,  2.44it/s]
```

```
342/342 ━━━━━━━━━━━━━━━━━━━━ 0s 449us/step
25/25 ━━━━━━━━━━━━━━━━━━━━ 0s 1ms/step
```

```
ExactExplainer explainer:   9%|████▊                                                  | 37/426 [00:21<02:39,  2.44it/s]
```

```
341/341 ━━━━━━━━━━━━━━━━━━━━ 0s 501us/step
25/25 ━━━━━━━━━━━━━━━━━━━━ 0s 1ms/step
```

```
ExactExplainer explainer:   9%|████▉                                                  | 38/426 [00:21<02:36,  2.47it/s]
```

```
349/349 ━━━━━━━━━━━━━━━━━━━━ 0s 474us/step
25/25 ━━━━━━━━━━━━━━━━━━━━ 0s 1ms/step
```

```
ExactExplainer explainer:   9%|█████                                                  | 39/426 [00:22<02:34,  2.50it/s]
```

```
357/357 ━━━━━━━━━━━━━━━━━━━━ 0s 458us/step
25/25 ━━━━━━━━━━━━━━━━━━━━ 0s 1ms/step
```

```
ExactExplainer explainer:   9%|█████▏                                                 | 40/426 [00:22<02:32,  2.53it/s]
```

```
365/365 ━━━━━━━━━━━━━━━━━━━━ 0s 610us/step
25/25 ━━━━━━━━━━━━━━━━━━━━ 0s 1ms/step
```

```
ExactExplainer explainer:  10%|█████▎                                                 | 41/426 [00:22<02:33,  2.51it/s]
```

```
357/357 ━━━━━━━━━━━━━━━━━━━━ 0s 481us/step
25/25 ━━━━━━━━━━━━━━━━━━━━ 0s 1ms/step
```

```
ExactExplainer explainer:  10%|█████▍                                                 | 42/426 [00:23<02:28,  2.58it/s]
```

```
351/351 ━━━━━━━━━━━━━━━━━━━━ 0s 546us/step
25/25 ━━━━━━━━━━━━━━━━━━━━ 0s 883us/step
```

```
ExactExplainer explainer:  10%|█████▌                                                 | 43/426 [00:23<02:25,  2.63it/s]
```

```
348/348 ━━━━━━━━━━━━━━━━━━━━ 0s 431us/step
25/25 ━━━━━━━━━━━━━━━━━━━━ 0s 2ms/step
```

```
ExactExplainer explainer:  10%|█████▋                                                 | 44/426 [00:24<02:25,  2.63it/s]
```

```
344/344 ━━━━━━━━━━━━━━━━━━━━ 0s 445us/step
25/25 ━━━━━━━━━━━━━━━━━━━━ 0s 987us/step
```

```
ExactExplainer explainer:  11%|█████▊                                                 | 45/426 [00:24<02:37,  2.42it/s]
```

```
371/371 ━━━━━━━━━━━━━━━━━━━━ 0s 440us/step
25/25 ━━━━━━━━━━━━━━━━━━━━ 0s 836us/step
```

```
ExactExplainer explainer:  11%|█████▉                                                 | 46/426 [00:24<02:28,  2.56it/s]
```

```
389/389 ━━━━━━━━━━━━━━━━━━━━ 0s 434us/step
25/25 ━━━━━━━━━━━━━━━━━━━━ 0s 1ms/step
```

```
ExactExplainer explainer:  11%|██████                                                 | 47/426 [00:25<02:27,  2.56it/s]
```

```
364/364 ━━━━━━━━━━━━━━━━━━━━ 0s 485us/step
25/25 ━━━━━━━━━━━━━━━━━━━━ 0s 1ms/step
```

```
ExactExplainer explainer:  11%|██████▏                                                | 48/426 [00:25<02:38,  2.39it/s]
```

```
367/367 ━━━━━━━━━━━━━━━━━━━━ 0s 438us/step
25/25 ━━━━━━━━━━━━━━━━━━━━ 0s 1ms/step
```

```
ExactExplainer explainer:  12%|██████▎                                                | 49/426 [00:26<02:44,  2.30it/s]
```

```
338/338 ━━━━━━━━━━━━━━━━━━━━ 0s 484us/step
25/25 ━━━━━━━━━━━━━━━━━━━━ 0s 915us/step
```

```
ExactExplainer explainer:  12%|██████▍                                                | 50/426 [00:26<02:38,  2.38it/s]
```

```
358/358 ━━━━━━━━━━━━━━━━━━━━ 0s 490us/step
25/25 ━━━━━━━━━━━━━━━━━━━━ 0s 1ms/step
```

```
ExactExplainer explainer:  12%|██████▌                                                | 51/426 [00:27<02:38,  2.36it/s]
```

```
380/380 ━━━━━━━━━━━━━━━━━━━━ 0s 455us/step
25/25 ━━━━━━━━━━━━━━━━━━━━ 0s 1ms/step
```

```
ExactExplainer explainer:  12%|██████▋                                                | 52/426 [00:27<02:34,  2.43it/s]
```

```
341/341 ━━━━━━━━━━━━━━━━━━━━ 0s 447us/step
25/25 ━━━━━━━━━━━━━━━━━━━━ 0s 1ms/step
```

```
ExactExplainer explainer:  12%|██████▊                                                | 53/426 [00:27<02:44,  2.27it/s]
```

```
371/371 ━━━━━━━━━━━━━━━━━━━━ 0s 453us/step
25/25 ━━━━━━━━━━━━━━━━━━━━ 0s 1ms/step
```

```
ExactExplainer explainer:  13%|██████▉                                                | 54/426 [00:28<02:37,  2.36it/s]
```

```
367/367 ━━━━━━━━━━━━━━━━━━━━ 0s 451us/step
25/25 ━━━━━━━━━━━━━━━━━━━━ 0s 929us/step
```

```
ExactExplainer explainer:  13%|███████                                                | 55/426 [00:28<02:26,  2.54it/s]
```

```
372/372 ━━━━━━━━━━━━━━━━━━━━ 0s 431us/step
25/25 ━━━━━━━━━━━━━━━━━━━━ 0s 1ms/step
```

```
ExactExplainer explainer:  13%|███████▏                                               | 56/426 [00:28<02:21,  2.61it/s]
```

```
373/373 ━━━━━━━━━━━━━━━━━━━━ 0s 465us/step
25/25 ━━━━━━━━━━━━━━━━━━━━ 0s 2ms/step
```

```
ExactExplainer explainer:  13%|███████▎                                               | 57/426 [00:29<02:33,  2.40it/s]
```

```
360/360 ━━━━━━━━━━━━━━━━━━━━ 0s 465us/step
25/25 ━━━━━━━━━━━━━━━━━━━━ 0s 902us/step
```

```
ExactExplainer explainer:  14%|███████▍                                               | 58/426 [00:29<02:27,  2.49it/s]
```

```
373/373 ━━━━━━━━━━━━━━━━━━━━ 0s 508us/step
25/25 ━━━━━━━━━━━━━━━━━━━━ 0s 850us/step
```

```
ExactExplainer explainer:  14%|███████▌                                               | 59/426 [00:30<02:25,  2.53it/s]
```

```
381/381 ━━━━━━━━━━━━━━━━━━━━ 0s 446us/step
25/25 ━━━━━━━━━━━━━━━━━━━━ 0s 1ms/step
```

```
ExactExplainer explainer:  14%|███████▋                                               | 60/426 [00:30<02:21,  2.59it/s]
```

```
358/358 ━━━━━━━━━━━━━━━━━━━━ 0s 463us/step
25/25 ━━━━━━━━━━━━━━━━━━━━ 0s 2ms/step
```

```
ExactExplainer explainer:  14%|███████▉                                               | 61/426 [00:30<02:20,  2.59it/s]
```

```
345/345 ━━━━━━━━━━━━━━━━━━━━ 0s 445us/step
25/25 ━━━━━━━━━━━━━━━━━━━━ 0s 1ms/step
```

```
ExactExplainer explainer:  15%|████████                                               | 62/426 [00:31<02:15,  2.68it/s]
```

```
357/357 ━━━━━━━━━━━━━━━━━━━━ 0s 469us/step
25/25 ━━━━━━━━━━━━━━━━━━━━ 0s 1ms/step
```

```
ExactExplainer explainer:  15%|████████▏                                              | 63/426 [00:31<02:29,  2.43it/s]
```

```
354/354 ━━━━━━━━━━━━━━━━━━━━ 0s 464us/step
25/25 ━━━━━━━━━━━━━━━━━━━━ 0s 971us/step
```

```
ExactExplainer explainer:  15%|████████▎                                              | 64/426 [00:32<02:22,  2.54it/s]
```

```
364/364 ━━━━━━━━━━━━━━━━━━━━ 0s 441us/step
25/25 ━━━━━━━━━━━━━━━━━━━━ 0s 1ms/step
```

```
ExactExplainer explainer:  15%|████████▍                                              | 65/426 [00:32<02:16,  2.64it/s]
```

```
342/342 ━━━━━━━━━━━━━━━━━━━━ 0s 451us/step
25/25 ━━━━━━━━━━━━━━━━━━━━ 0s 1ms/step
```

```
ExactExplainer explainer:  15%|████████▌                                              | 66/426 [00:32<02:27,  2.43it/s]
```

```
382/382 ━━━━━━━━━━━━━━━━━━━━ 0s 449us/step
25/25 ━━━━━━━━━━━━━━━━━━━━ 0s 1ms/step
```

```
ExactExplainer explainer:  16%|████████▋                                              | 67/426 [00:33<02:35,  2.31it/s]
```

```
379/379 ━━━━━━━━━━━━━━━━━━━━ 0s 447us/step
25/25 ━━━━━━━━━━━━━━━━━━━━ 0s 1ms/step
```

```
ExactExplainer explainer:  16%|████████▊                                              | 68/426 [00:33<02:43,  2.19it/s]
```

```
364/364 ━━━━━━━━━━━━━━━━━━━━ 0s 413us/step
25/25 ━━━━━━━━━━━━━━━━━━━━ 0s 1ms/step
```

```
ExactExplainer explainer:  16%|████████▉                                              | 69/426 [00:34<02:34,  2.31it/s]
```

```
351/351 ━━━━━━━━━━━━━━━━━━━━ 0s 457us/step
25/25 ━━━━━━━━━━━━━━━━━━━━ 0s 1ms/step
```

```
ExactExplainer explainer:  16%|█████████                                              | 70/426 [00:34<02:40,  2.22it/s]
```

```
370/370 ━━━━━━━━━━━━━━━━━━━━ 0s 459us/step
25/25 ━━━━━━━━━━━━━━━━━━━━ 0s 1ms/step
```

```
ExactExplainer explainer:  17%|█████████▏                                             | 71/426 [00:35<02:42,  2.19it/s]
```

```
345/345 ━━━━━━━━━━━━━━━━━━━━ 0s 544us/step
25/25 ━━━━━━━━━━━━━━━━━━━━ 0s 1ms/step
```

```
ExactExplainer explainer:  17%|█████████▎                                             | 72/426 [00:35<02:44,  2.15it/s]
```

```
363/363 ━━━━━━━━━━━━━━━━━━━━ 0s 686us/step
25/25 ━━━━━━━━━━━━━━━━━━━━ 0s 921us/step
```

```
ExactExplainer explainer:  17%|█████████▍                                             | 73/426 [00:36<02:42,  2.17it/s]
```

```
375/375 ━━━━━━━━━━━━━━━━━━━━ 0s 454us/step
25/25 ━━━━━━━━━━━━━━━━━━━━ 0s 1ms/step
```

```
ExactExplainer explainer:  17%|█████████▌                                             | 74/426 [00:36<02:32,  2.31it/s]
```

```
336/336 ━━━━━━━━━━━━━━━━━━━━ 0s 458us/step
25/25 ━━━━━━━━━━━━━━━━━━━━ 0s 1ms/step
```

```
ExactExplainer explainer:  18%|█████████▋                                             | 75/426 [00:37<02:38,  2.22it/s]
```

```
367/367 ━━━━━━━━━━━━━━━━━━━━ 0s 455us/step
25/25 ━━━━━━━━━━━━━━━━━━━━ 0s 1ms/step
```

```
ExactExplainer explainer:  18%|█████████▊                                             | 76/426 [00:37<02:29,  2.34it/s]
```

```
366/366 ━━━━━━━━━━━━━━━━━━━━ 0s 465us/step
25/25 ━━━━━━━━━━━━━━━━━━━━ 0s 1ms/step
```

```
ExactExplainer explainer:  18%|█████████▉                                             | 77/426 [00:37<02:25,  2.40it/s]
```

```
369/369 ━━━━━━━━━━━━━━━━━━━━ 0s 450us/step
25/25 ━━━━━━━━━━━━━━━━━━━━ 0s 976us/step
```

```
ExactExplainer explainer:  18%|██████████                                             | 78/426 [00:38<02:19,  2.50it/s]
```

```
350/350 ━━━━━━━━━━━━━━━━━━━━ 0s 480us/step
25/25 ━━━━━━━━━━━━━━━━━━━━ 0s 912us/step
```

```
ExactExplainer explainer:  19%|██████████▏                                            | 79/426 [00:38<02:19,  2.49it/s]
```

```
342/342 ━━━━━━━━━━━━━━━━━━━━ 0s 522us/step
25/25 ━━━━━━━━━━━━━━━━━━━━ 0s 982us/step
```

```
ExactExplainer explainer:  19%|██████████▎                                            | 80/426 [00:39<02:26,  2.36it/s]
```

```
349/349 ━━━━━━━━━━━━━━━━━━━━ 0s 472us/step
25/25 ━━━━━━━━━━━━━━━━━━━━ 0s 968us/step
```

```
ExactExplainer explainer:  19%|██████████▍                                            | 81/426 [00:39<02:18,  2.49it/s]
```

```
353/353 ━━━━━━━━━━━━━━━━━━━━ 0s 467us/step
25/25 ━━━━━━━━━━━━━━━━━━━━ 0s 2ms/step
```

```
ExactExplainer explainer:  19%|██████████▌                                            | 82/426 [00:39<02:17,  2.50it/s]
```

```
351/351 ━━━━━━━━━━━━━━━━━━━━ 0s 511us/step
25/25 ━━━━━━━━━━━━━━━━━━━━ 0s 973us/step
```

```
ExactExplainer explainer:  19%|██████████▋                                            | 83/426 [00:40<02:16,  2.52it/s]
```

```
357/357 ━━━━━━━━━━━━━━━━━━━━ 0s 445us/step
25/25 ━━━━━━━━━━━━━━━━━━━━ 0s 921us/step
```

```
ExactExplainer explainer:  20%|██████████▊                                            | 84/426 [00:40<02:23,  2.38it/s]
```

```
357/357 ━━━━━━━━━━━━━━━━━━━━ 0s 516us/step
25/25 ━━━━━━━━━━━━━━━━━━━━ 0s 1ms/step
```

```
ExactExplainer explainer:  20%|██████████▉                                            | 85/426 [00:41<02:31,  2.26it/s]
```

```
378/378 ━━━━━━━━━━━━━━━━━━━━ 0s 397us/step
25/25 ━━━━━━━━━━━━━━━━━━━━ 0s 1ms/step
```

```
ExactExplainer explainer:  20%|███████████                                            | 86/426 [00:41<02:25,  2.34it/s]
```

```
369/369 ━━━━━━━━━━━━━━━━━━━━ 0s 487us/step
25/25 ━━━━━━━━━━━━━━━━━━━━ 0s 915us/step
```

```
ExactExplainer explainer:  20%|███████████▏                                           | 87/426 [00:41<02:19,  2.42it/s]
```

```
348/348 ━━━━━━━━━━━━━━━━━━━━ 0s 575us/step
25/25 ━━━━━━━━━━━━━━━━━━━━ 0s 1ms/step
```

```
ExactExplainer explainer:  21%|███████████▎                                           | 88/426 [00:42<02:20,  2.41it/s]
```

```
361/361 ━━━━━━━━━━━━━━━━━━━━ 0s 486us/step
25/25 ━━━━━━━━━━━━━━━━━━━━ 0s 2ms/step
```

```
ExactExplainer explainer:  21%|███████████▍                                           | 89/426 [00:42<02:19,  2.42it/s]
```

```
341/341 ━━━━━━━━━━━━━━━━━━━━ 0s 521us/step
25/25 ━━━━━━━━━━━━━━━━━━━━ 0s 1ms/step
```

```
ExactExplainer explainer:  21%|███████████▌                                           | 90/426 [00:43<02:17,  2.45it/s]
```

```
347/347 ━━━━━━━━━━━━━━━━━━━━ 0s 537us/step
25/25 ━━━━━━━━━━━━━━━━━━━━ 0s 1ms/step
```

```
ExactExplainer explainer:  21%|███████████▋                                           | 91/426 [00:43<02:26,  2.29it/s]
```

```
341/341 ━━━━━━━━━━━━━━━━━━━━ 0s 462us/step
25/25 ━━━━━━━━━━━━━━━━━━━━ 0s 921us/step
```

```
ExactExplainer explainer:  22%|███████████▉                                           | 92/426 [00:44<02:17,  2.42it/s]
```

```
371/371 ━━━━━━━━━━━━━━━━━━━━ 0s 433us/step
25/25 ━━━━━━━━━━━━━━━━━━━━ 0s 855us/step
```

```
ExactExplainer explainer:  22%|████████████                                           | 93/426 [00:44<02:08,  2.59it/s]
```

```
363/363 ━━━━━━━━━━━━━━━━━━━━ 0s 522us/step
25/25 ━━━━━━━━━━━━━━━━━━━━ 0s 993us/step
```

```
ExactExplainer explainer:  22%|████████████▏                                          | 94/426 [00:44<02:05,  2.64it/s]
```

```
357/357 ━━━━━━━━━━━━━━━━━━━━ 0s 465us/step
25/25 ━━━━━━━━━━━━━━━━━━━━ 0s 896us/step
```

```
ExactExplainer explainer:  22%|████████████▎                                          | 95/426 [00:45<02:01,  2.72it/s]
```

```
349/349 ━━━━━━━━━━━━━━━━━━━━ 0s 504us/step
25/25 ━━━━━━━━━━━━━━━━━━━━ 0s 1ms/step
```

```
ExactExplainer explainer:  23%|████████████▍                                          | 96/426 [00:45<02:06,  2.61it/s]
```

```
359/359 ━━━━━━━━━━━━━━━━━━━━ 0s 479us/step
25/25 ━━━━━━━━━━━━━━━━━━━━ 0s 1ms/step
```

```
ExactExplainer explainer:  23%|████████████▌                                          | 97/426 [00:46<02:16,  2.41it/s]
```

```
350/350 ━━━━━━━━━━━━━━━━━━━━ 0s 457us/step
25/25 ━━━━━━━━━━━━━━━━━━━━ 0s 1ms/step
```

```
ExactExplainer explainer:  23%|████████████▋                                          | 98/426 [00:46<02:13,  2.46it/s]
```

```
342/342 ━━━━━━━━━━━━━━━━━━━━ 0s 445us/step
25/25 ━━━━━━━━━━━━━━━━━━━━ 0s 988us/step
```

```
ExactExplainer explainer:  23%|████████████▊                                          | 99/426 [00:46<02:05,  2.60it/s]
```

```
349/349 ━━━━━━━━━━━━━━━━━━━━ 0s 462us/step
25/25 ━━━━━━━━━━━━━━━━━━━━ 0s 1ms/step
```

```
ExactExplainer explainer:  23%|████████████▋                                         | 100/426 [00:47<02:14,  2.42it/s]
```

```
347/347 ━━━━━━━━━━━━━━━━━━━━ 0s 447us/step
25/25 ━━━━━━━━━━━━━━━━━━━━ 0s 1ms/step
```

```
ExactExplainer explainer:  24%|████████████▊                                         | 101/426 [00:47<02:09,  2.51it/s]
```

```
353/353 ━━━━━━━━━━━━━━━━━━━━ 0s 557us/step
25/25 ━━━━━━━━━━━━━━━━━━━━ 0s 1ms/step
```

```
ExactExplainer explainer:  24%|████████████▉                                         | 102/426 [00:48<02:13,  2.43it/s]
```

```
374/374 ━━━━━━━━━━━━━━━━━━━━ 0s 430us/step
25/25 ━━━━━━━━━━━━━━━━━━━━ 0s 1ms/step
```

```
ExactExplainer explainer:  24%|█████████████                                         | 103/426 [00:48<02:11,  2.45it/s]
```

```
361/361 ━━━━━━━━━━━━━━━━━━━━ 0s 491us/step
25/25 ━━━━━━━━━━━━━━━━━━━━ 0s 1ms/step
```

```
ExactExplainer explainer:  24%|█████████████▏                                        | 104/426 [00:48<02:06,  2.55it/s]
```

```
376/376 ━━━━━━━━━━━━━━━━━━━━ 0s 732us/step
25/25 ━━━━━━━━━━━━━━━━━━━━ 0s 2ms/step
```

```
ExactExplainer explainer:  25%|█████████████▎                                        | 105/426 [00:49<02:18,  2.31it/s]
```

```
343/343 ━━━━━━━━━━━━━━━━━━━━ 0s 375us/step
25/25 ━━━━━━━━━━━━━━━━━━━━ 0s 1ms/step
```

```
ExactExplainer explainer:  25%|█████████████▍                                        | 106/426 [00:49<02:08,  2.49it/s]
```

```
374/374 ━━━━━━━━━━━━━━━━━━━━ 0s 636us/step
25/25 ━━━━━━━━━━━━━━━━━━━━ 0s 1ms/step
```

```
ExactExplainer explainer:  25%|█████████████▌                                        | 107/426 [00:50<02:10,  2.44it/s]
```

```
337/337 ━━━━━━━━━━━━━━━━━━━━ 0s 818us/step
25/25 ━━━━━━━━━━━━━━━━━━━━ 0s 1ms/step
```

```
ExactExplainer explainer:  25%|█████████████▋                                        | 108/426 [00:50<02:18,  2.29it/s]
```

```
344/344 ━━━━━━━━━━━━━━━━━━━━ 0s 578us/step
25/25 ━━━━━━━━━━━━━━━━━━━━ 0s 995us/step
```

```
ExactExplainer explainer:  26%|█████████████▊                                        | 109/426 [00:51<02:25,  2.18it/s]
```

```
344/344 ━━━━━━━━━━━━━━━━━━━━ 0s 489us/step
25/25 ━━━━━━━━━━━━━━━━━━━━ 0s 926us/step
```

```
ExactExplainer explainer:  26%|█████████████▉                                        | 110/426 [00:51<02:12,  2.38it/s]
```

```
363/363 ━━━━━━━━━━━━━━━━━━━━ 0s 477us/step
25/25 ━━━━━━━━━━━━━━━━━━━━ 0s 1ms/step
```

```
ExactExplainer explainer:  26%|██████████████                                        | 111/426 [00:51<02:07,  2.46it/s]
```

```
350/350 ━━━━━━━━━━━━━━━━━━━━ 0s 472us/step
25/25 ━━━━━━━━━━━━━━━━━━━━ 0s 1ms/step
```

```
ExactExplainer explainer:  26%|██████████████▏                                       | 112/426 [00:52<02:00,  2.62it/s]
```

```
353/353 ━━━━━━━━━━━━━━━━━━━━ 0s 424us/step
25/25 ━━━━━━━━━━━━━━━━━━━━ 0s 1ms/step
```

```
ExactExplainer explainer:  27%|██████████████▎                                       | 113/426 [00:52<01:55,  2.72it/s]
```

```
376/376 ━━━━━━━━━━━━━━━━━━━━ 0s 496us/step
25/25 ━━━━━━━━━━━━━━━━━━━━ 0s 2ms/step
```

```
ExactExplainer explainer:  27%|██████████████▍                                       | 114/426 [00:52<02:00,  2.59it/s]
```

```
346/346 ━━━━━━━━━━━━━━━━━━━━ 0s 481us/step
25/25 ━━━━━━━━━━━━━━━━━━━━ 0s 966us/step
```

```
ExactExplainer explainer:  27%|██████████████▌                                       | 115/426 [00:53<02:04,  2.51it/s]
```

```
364/364 ━━━━━━━━━━━━━━━━━━━━ 0s 442us/step
25/25 ━━━━━━━━━━━━━━━━━━━━ 0s 2ms/step
```

```
ExactExplainer explainer:  27%|██████████████▋                                       | 116/426 [00:53<02:01,  2.54it/s]
```

```
341/341 ━━━━━━━━━━━━━━━━━━━━ 0s 594us/step
25/25 ━━━━━━━━━━━━━━━━━━━━ 0s 941us/step
```

```
ExactExplainer explainer:  27%|██████████████▊                                       | 117/426 [00:54<02:00,  2.57it/s]
```

```
356/356 ━━━━━━━━━━━━━━━━━━━━ 0s 480us/step
25/25 ━━━━━━━━━━━━━━━━━━━━ 0s 977us/step
```

```
ExactExplainer explainer:  28%|██████████████▉                                       | 118/426 [00:54<02:00,  2.56it/s]
```

```
378/378 ━━━━━━━━━━━━━━━━━━━━ 0s 474us/step
25/25 ━━━━━━━━━━━━━━━━━━━━ 0s 1ms/step
```

```
ExactExplainer explainer:  28%|███████████████                                       | 119/426 [00:54<01:58,  2.60it/s]
```

```
353/353 ━━━━━━━━━━━━━━━━━━━━ 0s 508us/step
25/25 ━━━━━━━━━━━━━━━━━━━━ 0s 1ms/step
```

```
ExactExplainer explainer:  28%|███████████████▏                                      | 120/426 [00:55<02:03,  2.48it/s]
```

```
359/359 ━━━━━━━━━━━━━━━━━━━━ 0s 518us/step
25/25 ━━━━━━━━━━━━━━━━━━━━ 0s 1ms/step
```

```
ExactExplainer explainer:  28%|███████████████▎                                      | 121/426 [00:55<02:03,  2.47it/s]
```

```
360/360 ━━━━━━━━━━━━━━━━━━━━ 0s 503us/step
25/25 ━━━━━━━━━━━━━━━━━━━━ 0s 1ms/step
```

```
ExactExplainer explainer:  29%|███████████████▍                                      | 122/426 [00:56<01:59,  2.53it/s]
```

```
365/365 ━━━━━━━━━━━━━━━━━━━━ 0s 474us/step
25/25 ━━━━━━━━━━━━━━━━━━━━ 0s 1ms/step
```

```
ExactExplainer explainer:  29%|███████████████▌                                      | 123/426 [00:56<01:55,  2.62it/s]
```

```
383/383 ━━━━━━━━━━━━━━━━━━━━ 0s 509us/step
25/25 ━━━━━━━━━━━━━━━━━━━━ 0s 2ms/step
```

```
ExactExplainer explainer:  29%|███████████████▋                                      | 124/426 [00:56<02:01,  2.48it/s]
```

```
364/364 ━━━━━━━━━━━━━━━━━━━━ 0s 624us/step
25/25 ━━━━━━━━━━━━━━━━━━━━ 0s 2ms/step
```

```
ExactExplainer explainer:  29%|███████████████▊                                      | 125/426 [00:57<02:03,  2.43it/s]
```

```
384/384 ━━━━━━━━━━━━━━━━━━━━ 0s 500us/step
25/25 ━━━━━━━━━━━━━━━━━━━━ 0s 1ms/step
```

```
ExactExplainer explainer:  30%|███████████████▉                                      | 126/426 [00:57<02:02,  2.44it/s]
```

```
347/347 ━━━━━━━━━━━━━━━━━━━━ 0s 483us/step
25/25 ━━━━━━━━━━━━━━━━━━━━ 0s 1ms/step
```

```
ExactExplainer explainer:  30%|████████████████                                      | 127/426 [00:58<01:59,  2.51it/s]
```

```
351/351 ━━━━━━━━━━━━━━━━━━━━ 0s 517us/step
25/25 ━━━━━━━━━━━━━━━━━━━━ 0s 1ms/step
```

```
ExactExplainer explainer:  30%|████████████████▏                                     | 128/426 [00:58<01:56,  2.57it/s]
```

```
358/358 ━━━━━━━━━━━━━━━━━━━━ 0s 510us/step
25/25 ━━━━━━━━━━━━━━━━━━━━ 0s 1ms/step
```

```
ExactExplainer explainer:  30%|████████████████▎                                     | 129/426 [00:58<02:02,  2.42it/s]
```

```
371/371 ━━━━━━━━━━━━━━━━━━━━ 0s 483us/step
25/25 ━━━━━━━━━━━━━━━━━━━━ 0s 1ms/step
```

```
ExactExplainer explainer:  31%|████████████████▍                                     | 130/426 [00:59<01:58,  2.49it/s]
```

```
375/375 ━━━━━━━━━━━━━━━━━━━━ 0s 452us/step
25/25 ━━━━━━━━━━━━━━━━━━━━ 0s 1ms/step
```

```
ExactExplainer explainer:  31%|████████████████▌                                     | 131/426 [00:59<01:55,  2.55it/s]
```

```
351/351 ━━━━━━━━━━━━━━━━━━━━ 0s 482us/step
25/25 ━━━━━━━━━━━━━━━━━━━━ 0s 1ms/step
```

```
ExactExplainer explainer:  31%|████████████████▋                                     | 132/426 [01:00<01:54,  2.57it/s]
```

```
358/358 ━━━━━━━━━━━━━━━━━━━━ 0s 470us/step
25/25 ━━━━━━━━━━━━━━━━━━━━ 0s 1ms/step
```

```
ExactExplainer explainer:  31%|████████████████▊                                     | 133/426 [01:00<01:48,  2.69it/s]
```

```
374/374 ━━━━━━━━━━━━━━━━━━━━ 0s 455us/step
25/25 ━━━━━━━━━━━━━━━━━━━━ 0s 843us/step
```

```
ExactExplainer explainer:  31%|████████████████▉                                     | 134/426 [01:00<01:45,  2.77it/s]
```

```
354/354 ━━━━━━━━━━━━━━━━━━━━ 0s 443us/step
25/25 ━━━━━━━━━━━━━━━━━━━━ 0s 914us/step
```

```
ExactExplainer explainer:  32%|█████████████████                                     | 135/426 [01:01<01:42,  2.85it/s]
```

```
352/352 ━━━━━━━━━━━━━━━━━━━━ 0s 522us/step
25/25 ━━━━━━━━━━━━━━━━━━━━ 0s 1ms/step
```

```
ExactExplainer explainer:  32%|█████████████████▏                                    | 136/426 [01:01<01:44,  2.78it/s]
```

```
349/349 ━━━━━━━━━━━━━━━━━━━━ 0s 447us/step
25/25 ━━━━━━━━━━━━━━━━━━━━ 0s 2ms/step
```

```
ExactExplainer explainer:  32%|█████████████████▎                                    | 137/426 [01:01<01:46,  2.71it/s]
```

```
339/339 ━━━━━━━━━━━━━━━━━━━━ 0s 483us/step
25/25 ━━━━━━━━━━━━━━━━━━━━ 0s 1ms/step
```

```
ExactExplainer explainer:  32%|█████████████████▍                                    | 138/426 [01:02<01:57,  2.45it/s]
```

```
366/366 ━━━━━━━━━━━━━━━━━━━━ 0s 446us/step
25/25 ━━━━━━━━━━━━━━━━━━━━ 0s 1ms/step
```

```
ExactExplainer explainer:  33%|█████████████████▌                                    | 139/426 [01:02<01:51,  2.58it/s]
```

```
353/353 ━━━━━━━━━━━━━━━━━━━━ 0s 672us/step
25/25 ━━━━━━━━━━━━━━━━━━━━ 0s 1ms/step
```

```
ExactExplainer explainer:  33%|█████████████████▋                                    | 140/426 [01:03<02:00,  2.38it/s]
```

```
356/356 ━━━━━━━━━━━━━━━━━━━━ 0s 384us/step
25/25 ━━━━━━━━━━━━━━━━━━━━ 0s 969us/step
```

```
ExactExplainer explainer:  33%|█████████████████▊                                    | 141/426 [01:03<02:06,  2.26it/s]
```

```
352/352 ━━━━━━━━━━━━━━━━━━━━ 0s 442us/step
25/25 ━━━━━━━━━━━━━━━━━━━━ 0s 1ms/step
```

```
ExactExplainer explainer:  33%|██████████████████                                    | 142/426 [01:03<01:56,  2.43it/s]
```

```
349/349 ━━━━━━━━━━━━━━━━━━━━ 0s 515us/step
25/25 ━━━━━━━━━━━━━━━━━━━━ 0s 1ms/step
```

```
ExactExplainer explainer:  34%|██████████████████▏                                   | 143/426 [01:04<02:03,  2.29it/s]
```

```
380/380 ━━━━━━━━━━━━━━━━━━━━ 0s 505us/step
25/25 ━━━━━━━━━━━━━━━━━━━━ 0s 947us/step
```

```
ExactExplainer explainer:  34%|██████████████████▎                                   | 144/426 [01:04<02:06,  2.23it/s]
```

```
362/362 ━━━━━━━━━━━━━━━━━━━━ 0s 483us/step
25/25 ━━━━━━━━━━━━━━━━━━━━ 0s 924us/step
```

```
ExactExplainer explainer:  34%|██████████████████▍                                   | 145/426 [01:05<01:57,  2.38it/s]
```

```
350/350 ━━━━━━━━━━━━━━━━━━━━ 0s 423us/step
25/25 ━━━━━━━━━━━━━━━━━━━━ 0s 1ms/step
```

```
ExactExplainer explainer:  34%|██████████████████▌                                   | 146/426 [01:05<01:49,  2.57it/s]
```

```
341/341 ━━━━━━━━━━━━━━━━━━━━ 0s 558us/step
25/25 ━━━━━━━━━━━━━━━━━━━━ 0s 1ms/step
```

```
ExactExplainer explainer:  35%|██████████████████▋                                   | 147/426 [01:05<01:46,  2.63it/s]
```

```
335/335 ━━━━━━━━━━━━━━━━━━━━ 0s 515us/step
25/25 ━━━━━━━━━━━━━━━━━━━━ 0s 944us/step
```

```
ExactExplainer explainer:  35%|██████████████████▊                                   | 148/426 [01:06<01:43,  2.68it/s]
```

```
356/356 ━━━━━━━━━━━━━━━━━━━━ 0s 511us/step
25/25 ━━━━━━━━━━━━━━━━━━━━ 0s 996us/step
```

```
ExactExplainer explainer:  35%|██████████████████▉                                   | 149/426 [01:06<01:44,  2.66it/s]
```

```
348/348 ━━━━━━━━━━━━━━━━━━━━ 0s 458us/step
25/25 ━━━━━━━━━━━━━━━━━━━━ 0s 996us/step
```

```
ExactExplainer explainer:  35%|███████████████████                                   | 150/426 [01:07<01:52,  2.46it/s]
```

```
356/356 ━━━━━━━━━━━━━━━━━━━━ 0s 464us/step
25/25 ━━━━━━━━━━━━━━━━━━━━ 0s 968us/step
```

```
ExactExplainer explainer:  35%|███████████████████▏                                  | 151/426 [01:07<01:48,  2.53it/s]
```

```
336/336 ━━━━━━━━━━━━━━━━━━━━ 0s 519us/step
25/25 ━━━━━━━━━━━━━━━━━━━━ 0s 1ms/step
```

```
ExactExplainer explainer:  36%|███████████████████▎                                  | 152/426 [01:07<01:49,  2.50it/s]
```

```
338/338 ━━━━━━━━━━━━━━━━━━━━ 0s 455us/step
25/25 ━━━━━━━━━━━━━━━━━━━━ 0s 909us/step
```

```
ExactExplainer explainer:  36%|███████████████████▍                                  | 153/426 [01:08<01:48,  2.52it/s]
```

```
383/383 ━━━━━━━━━━━━━━━━━━━━ 0s 452us/step
25/25 ━━━━━━━━━━━━━━━━━━━━ 0s 2ms/step
```

```
ExactExplainer explainer:  36%|███████████████████▌                                  | 154/426 [01:08<01:50,  2.47it/s]
```

```
354/354 ━━━━━━━━━━━━━━━━━━━━ 0s 458us/step
25/25 ━━━━━━━━━━━━━━━━━━━━ 0s 1ms/step
```

```
ExactExplainer explainer:  36%|███████████████████▋                                  | 155/426 [01:09<01:47,  2.52it/s]
```

```
357/357 ━━━━━━━━━━━━━━━━━━━━ 0s 525us/step
25/25 ━━━━━━━━━━━━━━━━━━━━ 0s 986us/step
```

```
ExactExplainer explainer:  37%|███████████████████▊                                  | 156/426 [01:09<01:46,  2.54it/s]
```

```
352/352 ━━━━━━━━━━━━━━━━━━━━ 0s 474us/step
25/25 ━━━━━━━━━━━━━━━━━━━━ 0s 956us/step
```

```
ExactExplainer explainer:  37%|███████████████████▉                                  | 157/426 [01:10<01:52,  2.39it/s]
```

```
363/363 ━━━━━━━━━━━━━━━━━━━━ 0s 497us/step
25/25 ━━━━━━━━━━━━━━━━━━━━ 0s 908us/step
```

```
ExactExplainer explainer:  37%|████████████████████                                  | 158/426 [01:10<01:45,  2.54it/s]
```

```
362/362 ━━━━━━━━━━━━━━━━━━━━ 0s 483us/step
25/25 ━━━━━━━━━━━━━━━━━━━━ 0s 1ms/step
```

```
ExactExplainer explainer:  37%|████████████████████▏                                 | 159/426 [01:10<01:44,  2.55it/s]
```

```
336/336 ━━━━━━━━━━━━━━━━━━━━ 0s 495us/step
25/25 ━━━━━━━━━━━━━━━━━━━━ 0s 902us/step
```

```
ExactExplainer explainer:  38%|████████████████████▎                                 | 160/426 [01:11<01:41,  2.63it/s]
```

```
358/358 ━━━━━━━━━━━━━━━━━━━━ 0s 422us/step
25/25 ━━━━━━━━━━━━━━━━━━━━ 0s 971us/step
```

```
ExactExplainer explainer:  38%|████████████████████▍                                 | 161/426 [01:11<01:36,  2.76it/s]
```

```
345/345 ━━━━━━━━━━━━━━━━━━━━ 0s 476us/step
25/25 ━━━━━━━━━━━━━━━━━━━━ 0s 2ms/step
```

```
ExactExplainer explainer:  38%|████████████████████▌                                 | 162/426 [01:11<01:36,  2.73it/s]
```

```
361/361 ━━━━━━━━━━━━━━━━━━━━ 0s 440us/step
25/25 ━━━━━━━━━━━━━━━━━━━━ 0s 1ms/step
```

```
ExactExplainer explainer:  38%|████████████████████▋                                 | 163/426 [01:12<01:44,  2.51it/s]
```

```
337/337 ━━━━━━━━━━━━━━━━━━━━ 0s 461us/step
25/25 ━━━━━━━━━━━━━━━━━━━━ 0s 1ms/step
```

```
ExactExplainer explainer:  38%|████████████████████▊                                 | 164/426 [01:12<01:40,  2.60it/s]
```

```
344/344 ━━━━━━━━━━━━━━━━━━━━ 0s 595us/step
25/25 ━━━━━━━━━━━━━━━━━━━━ 0s 1ms/step
```

```
ExactExplainer explainer:  39%|████████████████████▉                                 | 165/426 [01:13<01:41,  2.57it/s]
```

```
350/350 ━━━━━━━━━━━━━━━━━━━━ 0s 491us/step
25/25 ━━━━━━━━━━━━━━━━━━━━ 0s 1ms/step
```

```
ExactExplainer explainer:  39%|█████████████████████                                 | 166/426 [01:13<01:38,  2.64it/s]
```

```
364/364 ━━━━━━━━━━━━━━━━━━━━ 0s 454us/step
25/25 ━━━━━━━━━━━━━━━━━━━━ 0s 965us/step
```

```
ExactExplainer explainer:  39%|█████████████████████▏                                | 167/426 [01:13<01:44,  2.47it/s]
```

```
355/355 ━━━━━━━━━━━━━━━━━━━━ 0s 439us/step
25/25 ━━━━━━━━━━━━━━━━━━━━ 0s 999us/step
```

```
ExactExplainer explainer:  39%|█████████████████████▎                                | 168/426 [01:14<01:39,  2.59it/s]
```

```
340/340 ━━━━━━━━━━━━━━━━━━━━ 0s 620us/step
25/25 ━━━━━━━━━━━━━━━━━━━━ 0s 928us/step
```

```
ExactExplainer explainer:  40%|█████████████████████▍                                | 169/426 [01:14<01:39,  2.58it/s]
```

```
370/370 ━━━━━━━━━━━━━━━━━━━━ 0s 588us/step
25/25 ━━━━━━━━━━━━━━━━━━━━ 0s 1ms/step
```

```
ExactExplainer explainer:  40%|█████████████████████▌                                | 170/426 [01:14<01:41,  2.53it/s]
```

```
351/351 ━━━━━━━━━━━━━━━━━━━━ 0s 455us/step
25/25 ━━━━━━━━━━━━━━━━━━━━ 0s 2ms/step
```

```
ExactExplainer explainer:  40%|█████████████████████▋                                | 171/426 [01:15<01:48,  2.35it/s]
```

```
361/361 ━━━━━━━━━━━━━━━━━━━━ 0s 527us/step
25/25 ━━━━━━━━━━━━━━━━━━━━ 0s 980us/step
```

```
ExactExplainer explainer:  40%|█████████████████████▊                                | 172/426 [01:15<01:44,  2.42it/s]
```

```
350/350 ━━━━━━━━━━━━━━━━━━━━ 0s 533us/step
25/25 ━━━━━━━━━━━━━━━━━━━━ 0s 1ms/step
```

```
ExactExplainer explainer:  41%|█████████████████████▉                                | 173/426 [01:16<01:41,  2.49it/s]
```

```
360/360 ━━━━━━━━━━━━━━━━━━━━ 0s 466us/step
25/25 ━━━━━━━━━━━━━━━━━━━━ 0s 1ms/step
```

```
ExactExplainer explainer:  41%|██████████████████████                                | 174/426 [01:16<01:36,  2.61it/s]
```

```
358/358 ━━━━━━━━━━━━━━━━━━━━ 0s 472us/step
25/25 ━━━━━━━━━━━━━━━━━━━━ 0s 1ms/step
```

```
ExactExplainer explainer:  41%|██████████████████████▏                               | 175/426 [01:16<01:33,  2.69it/s]
```

```
353/353 ━━━━━━━━━━━━━━━━━━━━ 0s 495us/step
25/25 ━━━━━━━━━━━━━━━━━━━━ 0s 1ms/step
```

```
ExactExplainer explainer:  41%|██████████████████████▎                               | 176/426 [01:17<01:40,  2.48it/s]
```

```
357/357 ━━━━━━━━━━━━━━━━━━━━ 0s 453us/step
25/25 ━━━━━━━━━━━━━━━━━━━━ 0s 1ms/step
```

```
ExactExplainer explainer:  42%|██████████████████████▍                               | 177/426 [01:17<01:35,  2.61it/s]
```

```
345/345 ━━━━━━━━━━━━━━━━━━━━ 0s 680us/step
25/25 ━━━━━━━━━━━━━━━━━━━━ 0s 2ms/step
```

```
ExactExplainer explainer:  42%|██████████████████████▌                               | 178/426 [01:18<01:42,  2.42it/s]
```

```
365/365 ━━━━━━━━━━━━━━━━━━━━ 0s 620us/step
25/25 ━━━━━━━━━━━━━━━━━━━━ 0s 2ms/step
```

```
ExactExplainer explainer:  42%|██████████████████████▋                               | 179/426 [01:18<01:43,  2.39it/s]
```

```
366/366 ━━━━━━━━━━━━━━━━━━━━ 0s 516us/step
25/25 ━━━━━━━━━━━━━━━━━━━━ 0s 2ms/step
```

```
ExactExplainer explainer:  42%|██████████████████████▊                               | 180/426 [01:19<01:46,  2.32it/s]
```

```
360/360 ━━━━━━━━━━━━━━━━━━━━ 0s 908us/step
25/25 ━━━━━━━━━━━━━━━━━━━━ 0s 2ms/step
```

```
ExactExplainer explainer:  42%|██████████████████████▉                               | 181/426 [01:19<01:57,  2.08it/s]
```

```
341/341 ━━━━━━━━━━━━━━━━━━━━ 0s 647us/step
25/25 ━━━━━━━━━━━━━━━━━━━━ 0s 1ms/step
```

```
ExactExplainer explainer:  43%|███████████████████████                               | 182/426 [01:20<02:01,  2.02it/s]
```

```
372/372 ━━━━━━━━━━━━━━━━━━━━ 0s 475us/step
25/25 ━━━━━━━━━━━━━━━━━━━━ 0s 1ms/step
```

```
ExactExplainer explainer:  43%|███████████████████████▏                              | 183/426 [01:20<02:03,  1.97it/s]
```

```
360/360 ━━━━━━━━━━━━━━━━━━━━ 0s 597us/step
25/25 ━━━━━━━━━━━━━━━━━━━━ 0s 998us/step
```

```
ExactExplainer explainer:  43%|███████████████████████▎                              | 184/426 [01:21<01:56,  2.08it/s]
```

```
351/351 ━━━━━━━━━━━━━━━━━━━━ 0s 485us/step
25/25 ━━━━━━━━━━━━━━━━━━━━ 0s 1ms/step
```

```
ExactExplainer explainer:  43%|███████████████████████▍                              | 185/426 [01:21<01:46,  2.25it/s]
```

```
364/364 ━━━━━━━━━━━━━━━━━━━━ 0s 636us/step
25/25 ━━━━━━━━━━━━━━━━━━━━ 0s 1ms/step
```

```
ExactExplainer explainer:  44%|███████████████████████▌                              | 186/426 [01:21<01:47,  2.23it/s]
```

```
366/366 ━━━━━━━━━━━━━━━━━━━━ 0s 551us/step
25/25 ━━━━━━━━━━━━━━━━━━━━ 0s 1ms/step
```

```
ExactExplainer explainer:  44%|███████████████████████▋                              | 187/426 [01:22<01:49,  2.18it/s]
```

```
361/361 ━━━━━━━━━━━━━━━━━━━━ 0s 596us/step
25/25 ━━━━━━━━━━━━━━━━━━━━ 0s 1ms/step
```

```
ExactExplainer explainer:  44%|███████████████████████▊                              | 188/426 [01:22<01:50,  2.15it/s]
```

```
364/364 ━━━━━━━━━━━━━━━━━━━━ 0s 638us/step
25/25 ━━━━━━━━━━━━━━━━━━━━ 0s 1ms/step
```

```
ExactExplainer explainer:  44%|███████████████████████▉                              | 189/426 [01:23<01:54,  2.06it/s]
```

```
373/373 ━━━━━━━━━━━━━━━━━━━━ 0s 663us/step
25/25 ━━━━━━━━━━━━━━━━━━━━ 0s 2ms/step
```

```
ExactExplainer explainer:  45%|████████████████████████                              | 190/426 [01:24<01:56,  2.02it/s]
```

```
367/367 ━━━━━━━━━━━━━━━━━━━━ 0s 1ms/step  
25/25 ━━━━━━━━━━━━━━━━━━━━ 0s 1ms/step
```

```
ExactExplainer explainer:  45%|████████████████████████▏                             | 191/426 [01:24<02:07,  1.84it/s]
```

```
357/357 ━━━━━━━━━━━━━━━━━━━━ 0s 913us/step
25/25 ━━━━━━━━━━━━━━━━━━━━ 0s 1ms/step
```

```
ExactExplainer explainer:  45%|████████████████████████▎                             | 192/426 [01:25<02:12,  1.76it/s]
```

```
363/363 ━━━━━━━━━━━━━━━━━━━━ 0s 776us/step
25/25 ━━━━━━━━━━━━━━━━━━━━ 0s 2ms/step
```

```
ExactExplainer explainer:  45%|████████████████████████▍                             | 193/426 [01:25<02:10,  1.78it/s]
```

```
364/364 ━━━━━━━━━━━━━━━━━━━━ 0s 853us/step
25/25 ━━━━━━━━━━━━━━━━━━━━ 0s 2ms/step
```

```
ExactExplainer explainer:  46%|████████████████████████▌                             | 194/426 [01:26<02:13,  1.74it/s]
```

```
359/359 ━━━━━━━━━━━━━━━━━━━━ 0s 694us/step
25/25 ━━━━━━━━━━━━━━━━━━━━ 0s 1ms/step
```

```
ExactExplainer explainer:  46%|████████████████████████▋                             | 195/426 [01:26<02:06,  1.82it/s]
```

```
352/352 ━━━━━━━━━━━━━━━━━━━━ 0s 590us/step
25/25 ━━━━━━━━━━━━━━━━━━━━ 0s 1ms/step
```

```
ExactExplainer explainer:  46%|████████████████████████▊                             | 196/426 [01:27<01:59,  1.93it/s]
```

```
365/365 ━━━━━━━━━━━━━━━━━━━━ 0s 496us/step
25/25 ━━━━━━━━━━━━━━━━━━━━ 0s 1ms/step
```

```
ExactExplainer explainer:  46%|████████████████████████▉                             | 197/426 [01:27<01:49,  2.10it/s]
```

```
357/357 ━━━━━━━━━━━━━━━━━━━━ 0s 470us/step
25/25 ━━━━━━━━━━━━━━━━━━━━ 0s 1ms/step
```

```
ExactExplainer explainer:  46%|█████████████████████████                             | 198/426 [01:28<01:48,  2.10it/s]
```

```
371/371 ━━━━━━━━━━━━━━━━━━━━ 0s 498us/step
25/25 ━━━━━━━━━━━━━━━━━━━━ 0s 1ms/step
```

```
ExactExplainer explainer:  47%|█████████████████████████▏                            | 199/426 [01:28<01:40,  2.25it/s]
```

```
368/368 ━━━━━━━━━━━━━━━━━━━━ 0s 508us/step
25/25 ━━━━━━━━━━━━━━━━━━━━ 0s 951us/step
```

```
ExactExplainer explainer:  47%|█████████████████████████▎                            | 200/426 [01:28<01:36,  2.34it/s]
```

```
365/365 ━━━━━━━━━━━━━━━━━━━━ 0s 530us/step
25/25 ━━━━━━━━━━━━━━━━━━━━ 0s 1ms/step
```

```
ExactExplainer explainer:  47%|█████████████████████████▍                            | 201/426 [01:29<01:34,  2.38it/s]
```

```
350/350 ━━━━━━━━━━━━━━━━━━━━ 0s 468us/step
25/25 ━━━━━━━━━━━━━━━━━━━━ 0s 1ms/step
```

```
ExactExplainer explainer:  47%|█████████████████████████▌                            | 202/426 [01:29<01:28,  2.52it/s]
```

```
363/363 ━━━━━━━━━━━━━━━━━━━━ 0s 534us/step
25/25 ━━━━━━━━━━━━━━━━━━━━ 0s 1ms/step
```

```
ExactExplainer explainer:  48%|█████████████████████████▋                            | 203/426 [01:30<01:29,  2.48it/s]
```

```
348/348 ━━━━━━━━━━━━━━━━━━━━ 0s 640us/step
25/25 ━━━━━━━━━━━━━━━━━━━━ 0s 1ms/step
```

```
ExactExplainer explainer:  48%|█████████████████████████▊                            | 204/426 [01:30<01:36,  2.30it/s]
```

```
378/378 ━━━━━━━━━━━━━━━━━━━━ 0s 556us/step
25/25 ━━━━━━━━━━━━━━━━━━━━ 0s 2ms/step
```

```
ExactExplainer explainer:  48%|█████████████████████████▉                            | 205/426 [01:31<01:38,  2.24it/s]
```

```
338/338 ━━━━━━━━━━━━━━━━━━━━ 0s 675us/step
25/25 ━━━━━━━━━━━━━━━━━━━━ 0s 2ms/step
```

```
ExactExplainer explainer:  48%|██████████████████████████                            | 206/426 [01:31<01:45,  2.09it/s]
```

```
341/341 ━━━━━━━━━━━━━━━━━━━━ 0s 519us/step
25/25 ━━━━━━━━━━━━━━━━━━━━ 0s 1ms/step
```

```
ExactExplainer explainer:  49%|██████████████████████████▏                           | 207/426 [01:32<01:39,  2.21it/s]
```

```
363/363 ━━━━━━━━━━━━━━━━━━━━ 0s 989us/step
25/25 ━━━━━━━━━━━━━━━━━━━━ 0s 2ms/step
```

```
ExactExplainer explainer:  49%|██████████████████████████▎                           | 208/426 [01:32<01:51,  1.96it/s]
```

```
357/357 ━━━━━━━━━━━━━━━━━━━━ 0s 685us/step
25/25 ━━━━━━━━━━━━━━━━━━━━ 0s 1ms/step
```

```
ExactExplainer explainer:  49%|██████████████████████████▍                           | 209/426 [01:33<01:50,  1.96it/s]
```

```
347/347 ━━━━━━━━━━━━━━━━━━━━ 0s 687us/step
25/25 ━━━━━━━━━━━━━━━━━━━━ 0s 1ms/step
```

```
ExactExplainer explainer:  49%|██████████████████████████▌                           | 210/426 [01:33<01:46,  2.02it/s]
```

```
365/365 ━━━━━━━━━━━━━━━━━━━━ 0s 725us/step
25/25 ━━━━━━━━━━━━━━━━━━━━ 0s 2ms/step
```

```
ExactExplainer explainer:  50%|██████████████████████████▋                           | 211/426 [01:34<01:50,  1.95it/s]
```

```
357/357 ━━━━━━━━━━━━━━━━━━━━ 0s 697us/step
25/25 ━━━━━━━━━━━━━━━━━━━━ 0s 1ms/step
```

```
ExactExplainer explainer:  50%|██████████████████████████▊                           | 212/426 [01:34<01:53,  1.89it/s]
```

```
359/359 ━━━━━━━━━━━━━━━━━━━━ 0s 645us/step
25/25 ━━━━━━━━━━━━━━━━━━━━ 0s 1ms/step
```

```
ExactExplainer explainer:  50%|███████████████████████████                           | 213/426 [01:35<01:51,  1.91it/s]
```

```
373/373 ━━━━━━━━━━━━━━━━━━━━ 0s 835us/step
25/25 ━━━━━━━━━━━━━━━━━━━━ 0s 1ms/step
```

```
ExactExplainer explainer:  50%|███████████████████████████▏                          | 214/426 [01:35<01:53,  1.87it/s]
```

```
355/355 ━━━━━━━━━━━━━━━━━━━━ 0s 713us/step
25/25 ━━━━━━━━━━━━━━━━━━━━ 0s 2ms/step
```

```
ExactExplainer explainer:  50%|███████████████████████████▎                          | 215/426 [01:36<01:55,  1.83it/s]
```

```
363/363 ━━━━━━━━━━━━━━━━━━━━ 0s 636us/step
25/25 ━━━━━━━━━━━━━━━━━━━━ 0s 1ms/step
```

```
ExactExplainer explainer:  51%|███████████████████████████▍                          | 216/426 [01:36<01:51,  1.89it/s]
```

```
373/373 ━━━━━━━━━━━━━━━━━━━━ 0s 695us/step
25/25 ━━━━━━━━━━━━━━━━━━━━ 0s 1ms/step
```

```
ExactExplainer explainer:  51%|███████████████████████████▌                          | 217/426 [01:37<01:51,  1.88it/s]
```

```
336/336 ━━━━━━━━━━━━━━━━━━━━ 0s 705us/step
25/25 ━━━━━━━━━━━━━━━━━━━━ 0s 1ms/step
```

```
ExactExplainer explainer:  51%|███████████████████████████▋                          | 218/426 [01:38<01:49,  1.90it/s]
```

```
347/347 ━━━━━━━━━━━━━━━━━━━━ 0s 576us/step
25/25 ━━━━━━━━━━━━━━━━━━━━ 0s 2ms/step
```

```
ExactExplainer explainer:  51%|███████████████████████████▊                          | 219/426 [01:38<01:42,  2.03it/s]
```

```
360/360 ━━━━━━━━━━━━━━━━━━━━ 0s 722us/step
25/25 ━━━━━━━━━━━━━━━━━━━━ 0s 2ms/step
```

```
ExactExplainer explainer:  52%|███████████████████████████▉                          | 220/426 [01:38<01:44,  1.97it/s]
```

```
353/353 ━━━━━━━━━━━━━━━━━━━━ 0s 876us/step
25/25 ━━━━━━━━━━━━━━━━━━━━ 0s 2ms/step
```

```
ExactExplainer explainer:  52%|████████████████████████████                          | 221/426 [01:39<01:47,  1.90it/s]
```

```
350/350 ━━━━━━━━━━━━━━━━━━━━ 0s 406us/step
25/25 ━━━━━━━━━━━━━━━━━━━━ 0s 933us/step
```

```
ExactExplainer explainer:  52%|████████████████████████████▏                         | 222/426 [01:40<01:45,  1.93it/s]
```

```
335/335 ━━━━━━━━━━━━━━━━━━━━ 0s 486us/step
25/25 ━━━━━━━━━━━━━━━━━━━━ 0s 1ms/step
```

```
ExactExplainer explainer:  52%|████████████████████████████▎                         | 223/426 [01:40<01:34,  2.15it/s]
```

```
342/342 ━━━━━━━━━━━━━━━━━━━━ 0s 462us/step
25/25 ━━━━━━━━━━━━━━━━━━━━ 0s 1ms/step
```

```
ExactExplainer explainer:  53%|████████████████████████████▍                         | 224/426 [01:40<01:27,  2.30it/s]
```

```
359/359 ━━━━━━━━━━━━━━━━━━━━ 0s 598us/step
25/25 ━━━━━━━━━━━━━━━━━━━━ 0s 929us/step
```

```
ExactExplainer explainer:  53%|████████████████████████████▌                         | 225/426 [01:41<01:25,  2.36it/s]
```

```
359/359 ━━━━━━━━━━━━━━━━━━━━ 0s 737us/step
25/25 ━━━━━━━━━━━━━━━━━━━━ 0s 1ms/step
```

```
ExactExplainer explainer:  53%|████████████████████████████▋                         | 226/426 [01:41<01:30,  2.21it/s]
```

```
377/377 ━━━━━━━━━━━━━━━━━━━━ 0s 752us/step
25/25 ━━━━━━━━━━━━━━━━━━━━ 0s 2ms/step
```

```
ExactExplainer explainer:  53%|████████████████████████████▊                         | 227/426 [01:42<01:36,  2.07it/s]
```

```
341/341 ━━━━━━━━━━━━━━━━━━━━ 0s 914us/step
25/25 ━━━━━━━━━━━━━━━━━━━━ 0s 2ms/step
```

```
ExactExplainer explainer:  54%|████████████████████████████▉                         | 228/426 [01:42<01:42,  1.94it/s]
```

```
357/357 ━━━━━━━━━━━━━━━━━━━━ 0s 626us/step
25/25 ━━━━━━━━━━━━━━━━━━━━ 0s 1ms/step
```

```
ExactExplainer explainer:  54%|█████████████████████████████                         | 229/426 [01:43<01:39,  1.99it/s]
```

```
376/376 ━━━━━━━━━━━━━━━━━━━━ 0s 752us/step
25/25 ━━━━━━━━━━━━━━━━━━━━ 0s 2ms/step
```

```
ExactExplainer explainer:  54%|█████████████████████████████▏                        | 230/426 [01:43<01:42,  1.90it/s]
```

```
367/367 ━━━━━━━━━━━━━━━━━━━━ 0s 528us/step
25/25 ━━━━━━━━━━━━━━━━━━━━ 0s 1ms/step
```

```
ExactExplainer explainer:  54%|█████████████████████████████▎                        | 231/426 [01:44<01:35,  2.04it/s]
```

```
362/362 ━━━━━━━━━━━━━━━━━━━━ 0s 669us/step
25/25 ━━━━━━━━━━━━━━━━━━━━ 0s 1ms/step
```

```
ExactExplainer explainer:  54%|█████████████████████████████▍                        | 232/426 [01:44<01:31,  2.12it/s]
```

```
351/351 ━━━━━━━━━━━━━━━━━━━━ 0s 481us/step
25/25 ━━━━━━━━━━━━━━━━━━━━ 0s 907us/step
```

```
ExactExplainer explainer:  55%|█████████████████████████████▌                        | 233/426 [01:45<01:25,  2.25it/s]
```

```
357/357 ━━━━━━━━━━━━━━━━━━━━ 0s 547us/step
25/25 ━━━━━━━━━━━━━━━━━━━━ 0s 986us/step
```

```
ExactExplainer explainer:  55%|█████████████████████████████▋                        | 234/426 [01:45<01:21,  2.37it/s]
```

```
362/362 ━━━━━━━━━━━━━━━━━━━━ 0s 455us/step
25/25 ━━━━━━━━━━━━━━━━━━━━ 0s 1ms/step
```

```
ExactExplainer explainer:  55%|█████████████████████████████▊                        | 235/426 [01:45<01:18,  2.45it/s]
```

```
347/347 ━━━━━━━━━━━━━━━━━━━━ 0s 636us/step
25/25 ━━━━━━━━━━━━━━━━━━━━ 0s 996us/step
```

```
ExactExplainer explainer:  55%|█████████████████████████████▉                        | 236/426 [01:46<01:21,  2.32it/s]
```

```
371/371 ━━━━━━━━━━━━━━━━━━━━ 0s 504us/step
25/25 ━━━━━━━━━━━━━━━━━━━━ 0s 873us/step
```

```
ExactExplainer explainer:  56%|██████████████████████████████                        | 237/426 [01:46<01:18,  2.39it/s]
```

```
359/359 ━━━━━━━━━━━━━━━━━━━━ 0s 454us/step
25/25 ━━━━━━━━━━━━━━━━━━━━ 0s 957us/step
```

```
ExactExplainer explainer:  56%|██████████████████████████████▏                       | 238/426 [01:47<01:14,  2.54it/s]
```

```
344/344 ━━━━━━━━━━━━━━━━━━━━ 0s 462us/step
25/25 ━━━━━━━━━━━━━━━━━━━━ 0s 1ms/step
```

```
ExactExplainer explainer:  56%|██████████████████████████████▎                       | 239/426 [01:47<01:17,  2.40it/s]
```

```
362/362 ━━━━━━━━━━━━━━━━━━━━ 0s 470us/step
25/25 ━━━━━━━━━━━━━━━━━━━━ 0s 1ms/step
```

```
ExactExplainer explainer:  56%|██████████████████████████████▍                       | 240/426 [01:47<01:21,  2.29it/s]
```

```
354/354 ━━━━━━━━━━━━━━━━━━━━ 0s 474us/step
25/25 ━━━━━━━━━━━━━━━━━━━━ 0s 959us/step
```

```
ExactExplainer explainer:  57%|██████████████████████████████▌                       | 241/426 [01:48<01:15,  2.44it/s]
```

```
375/375 ━━━━━━━━━━━━━━━━━━━━ 0s 428us/step
25/25 ━━━━━━━━━━━━━━━━━━━━ 0s 876us/step
```

```
ExactExplainer explainer:  57%|██████████████████████████████▋                       | 242/426 [01:48<01:10,  2.60it/s]
```

```
355/355 ━━━━━━━━━━━━━━━━━━━━ 0s 494us/step
25/25 ━━━━━━━━━━━━━━━━━━━━ 0s 1ms/step
```

```
ExactExplainer explainer:  57%|██████████████████████████████▊                       | 243/426 [01:48<01:08,  2.68it/s]
```

```
359/359 ━━━━━━━━━━━━━━━━━━━━ 0s 469us/step
25/25 ━━━━━━━━━━━━━━━━━━━━ 0s 853us/step
```

```
ExactExplainer explainer:  57%|██████████████████████████████▉                       | 244/426 [01:49<01:05,  2.78it/s]
```

```
379/379 ━━━━━━━━━━━━━━━━━━━━ 0s 472us/step
25/25 ━━━━━━━━━━━━━━━━━━━━ 0s 1ms/step
```

```
ExactExplainer explainer:  58%|███████████████████████████████                       | 245/426 [01:49<01:05,  2.75it/s]
```

```
359/359 ━━━━━━━━━━━━━━━━━━━━ 0s 482us/step
25/25 ━━━━━━━━━━━━━━━━━━━━ 0s 966us/step
```

```
ExactExplainer explainer:  58%|███████████████████████████████▏                      | 246/426 [01:50<01:05,  2.75it/s]
```

```
368/368 ━━━━━━━━━━━━━━━━━━━━ 0s 435us/step
25/25 ━━━━━━━━━━━━━━━━━━━━ 0s 914us/step
```

```
ExactExplainer explainer:  58%|███████████████████████████████▎                      | 247/426 [01:50<01:09,  2.57it/s]
```

```
364/364 ━━━━━━━━━━━━━━━━━━━━ 0s 493us/step
25/25 ━━━━━━━━━━━━━━━━━━━━ 0s 995us/step
```

```
ExactExplainer explainer:  58%|███████████████████████████████▍                      | 248/426 [01:50<01:10,  2.51it/s]
```

```
339/339 ━━━━━━━━━━━━━━━━━━━━ 0s 574us/step
25/25 ━━━━━━━━━━━━━━━━━━━━ 0s 1ms/step
```

```
ExactExplainer explainer:  58%|███████████████████████████████▌                      | 249/426 [01:51<01:17,  2.29it/s]
```

```
342/342 ━━━━━━━━━━━━━━━━━━━━ 0s 513us/step
25/25 ━━━━━━━━━━━━━━━━━━━━ 0s 1ms/step
```

```
ExactExplainer explainer:  59%|███████████████████████████████▋                      | 250/426 [01:51<01:14,  2.37it/s]
```

```
373/373 ━━━━━━━━━━━━━━━━━━━━ 0s 458us/step
25/25 ━━━━━━━━━━━━━━━━━━━━ 0s 943us/step
```

```
ExactExplainer explainer:  59%|███████████████████████████████▊                      | 251/426 [01:52<01:10,  2.48it/s]
```

```
389/389 ━━━━━━━━━━━━━━━━━━━━ 0s 475us/step
25/25 ━━━━━━━━━━━━━━━━━━━━ 0s 843us/step
```

```
ExactExplainer explainer:  59%|███████████████████████████████▉                      | 252/426 [01:52<01:12,  2.40it/s]
```

```
350/350 ━━━━━━━━━━━━━━━━━━━━ 0s 673us/step
25/25 ━━━━━━━━━━━━━━━━━━━━ 0s 1ms/step
```

```
ExactExplainer explainer:  59%|████████████████████████████████                      | 253/426 [01:53<01:13,  2.37it/s]
```

```
352/352 ━━━━━━━━━━━━━━━━━━━━ 0s 465us/step
25/25 ━━━━━━━━━━━━━━━━━━━━ 0s 959us/step
```

```
ExactExplainer explainer:  60%|████████████████████████████████▏                     | 254/426 [01:53<01:07,  2.55it/s]
```

```
392/392 ━━━━━━━━━━━━━━━━━━━━ 0s 500us/step
25/25 ━━━━━━━━━━━━━━━━━━━━ 0s 1ms/step
```

```
ExactExplainer explainer:  60%|████████████████████████████████▎                     | 255/426 [01:53<01:10,  2.41it/s]
```

```
343/343 ━━━━━━━━━━━━━━━━━━━━ 0s 518us/step
25/25 ━━━━━━━━━━━━━━━━━━━━ 0s 1ms/step
```

```
ExactExplainer explainer:  60%|████████████████████████████████▍                     | 256/426 [01:54<01:07,  2.51it/s]
```

```
356/356 ━━━━━━━━━━━━━━━━━━━━ 0s 456us/step
25/25 ━━━━━━━━━━━━━━━━━━━━ 0s 2ms/step
```

```
ExactExplainer explainer:  60%|████████████████████████████████▌                     | 257/426 [01:54<01:04,  2.61it/s]
```

```
362/362 ━━━━━━━━━━━━━━━━━━━━ 0s 517us/step
25/25 ━━━━━━━━━━━━━━━━━━━━ 0s 1ms/step
```

```
ExactExplainer explainer:  61%|████████████████████████████████▋                     | 258/426 [01:54<01:03,  2.66it/s]
```

```
363/363 ━━━━━━━━━━━━━━━━━━━━ 0s 581us/step
25/25 ━━━━━━━━━━━━━━━━━━━━ 0s 949us/step
```

```
ExactExplainer explainer:  61%|████████████████████████████████▊                     | 259/426 [01:55<01:06,  2.50it/s]
```

```
365/365 ━━━━━━━━━━━━━━━━━━━━ 0s 483us/step
25/25 ━━━━━━━━━━━━━━━━━━━━ 0s 961us/step
```

```
ExactExplainer explainer:  61%|████████████████████████████████▉                     | 260/426 [01:55<01:04,  2.59it/s]
```

```
378/378 ━━━━━━━━━━━━━━━━━━━━ 0s 459us/step
25/25 ━━━━━━━━━━━━━━━━━━━━ 0s 1ms/step
```

```
ExactExplainer explainer:  61%|█████████████████████████████████                     | 261/426 [01:56<01:03,  2.62it/s]
```

```
356/356 ━━━━━━━━━━━━━━━━━━━━ 0s 420us/step
25/25 ━━━━━━━━━━━━━━━━━━━━ 0s 1ms/step
```

```
ExactExplainer explainer:  62%|█████████████████████████████████▏                    | 262/426 [01:56<00:59,  2.75it/s]
```

```
368/368 ━━━━━━━━━━━━━━━━━━━━ 0s 526us/step
25/25 ━━━━━━━━━━━━━━━━━━━━ 0s 989us/step
```

```
ExactExplainer explainer:  62%|█████████████████████████████████▎                    | 263/426 [01:56<00:59,  2.73it/s]
```

```
342/342 ━━━━━━━━━━━━━━━━━━━━ 0s 679us/step
25/25 ━━━━━━━━━━━━━━━━━━━━ 0s 1ms/step
```

```
ExactExplainer explainer:  62%|█████████████████████████████████▍                    | 264/426 [01:57<01:03,  2.56it/s]
```

```
354/354 ━━━━━━━━━━━━━━━━━━━━ 0s 555us/step
25/25 ━━━━━━━━━━━━━━━━━━━━ 0s 1ms/step
```

```
ExactExplainer explainer:  62%|█████████████████████████████████▌                    | 265/426 [01:57<01:02,  2.58it/s]
```

```
357/357 ━━━━━━━━━━━━━━━━━━━━ 0s 431us/step
25/25 ━━━━━━━━━━━━━━━━━━━━ 0s 1ms/step
```

```
ExactExplainer explainer:  62%|█████████████████████████████████▋                    | 266/426 [01:57<00:59,  2.70it/s]
```

```
342/342 ━━━━━━━━━━━━━━━━━━━━ 0s 564us/step
25/25 ━━━━━━━━━━━━━━━━━━━━ 0s 1ms/step
```

```
ExactExplainer explainer:  63%|█████████████████████████████████▊                    | 267/426 [01:58<00:58,  2.73it/s]
```

```
366/366 ━━━━━━━━━━━━━━━━━━━━ 0s 834us/step
25/25 ━━━━━━━━━━━━━━━━━━━━ 0s 1ms/step
```

```
ExactExplainer explainer:  63%|█████████████████████████████████▉                    | 268/426 [01:58<01:04,  2.44it/s]
```

```
354/354 ━━━━━━━━━━━━━━━━━━━━ 0s 474us/step
25/25 ━━━━━━━━━━━━━━━━━━━━ 0s 1ms/step
```

```
ExactExplainer explainer:  63%|██████████████████████████████████                    | 269/426 [01:59<01:03,  2.45it/s]
```

```
366/366 ━━━━━━━━━━━━━━━━━━━━ 0s 472us/step
25/25 ━━━━━━━━━━━━━━━━━━━━ 0s 2ms/step
```

```
ExactExplainer explainer:  63%|██████████████████████████████████▏                   | 270/426 [01:59<01:02,  2.49it/s]
```

```
373/373 ━━━━━━━━━━━━━━━━━━━━ 0s 454us/step
25/25 ━━━━━━━━━━━━━━━━━━━━ 0s 991us/step
```

```
ExactExplainer explainer:  64%|██████████████████████████████████▎                   | 271/426 [02:00<01:00,  2.56it/s]
```

```
354/354 ━━━━━━━━━━━━━━━━━━━━ 0s 565us/step
25/25 ━━━━━━━━━━━━━━━━━━━━ 0s 1ms/step
```

```
ExactExplainer explainer:  64%|██████████████████████████████████▍                   | 272/426 [02:00<01:00,  2.53it/s]
```

```
347/347 ━━━━━━━━━━━━━━━━━━━━ 0s 538us/step
25/25 ━━━━━━━━━━━━━━━━━━━━ 0s 1ms/step
```

```
ExactExplainer explainer:  64%|██████████████████████████████████▌                   | 273/426 [02:00<01:00,  2.55it/s]
```

```
345/345 ━━━━━━━━━━━━━━━━━━━━ 0s 448us/step
25/25 ━━━━━━━━━━━━━━━━━━━━ 0s 2ms/step
```

```
ExactExplainer explainer:  64%|██████████████████████████████████▋                   | 274/426 [02:01<00:58,  2.61it/s]
```

```
355/355 ━━━━━━━━━━━━━━━━━━━━ 0s 549us/step
25/25 ━━━━━━━━━━━━━━━━━━━━ 0s 1ms/step
```

```
ExactExplainer explainer:  65%|██████████████████████████████████▊                   | 275/426 [02:01<00:57,  2.63it/s]
```

```
358/358 ━━━━━━━━━━━━━━━━━━━━ 0s 480us/step
25/25 ━━━━━━━━━━━━━━━━━━━━ 0s 1ms/step
```

```
ExactExplainer explainer:  65%|██████████████████████████████████▉                   | 276/426 [02:01<00:57,  2.60it/s]
```

```
358/358 ━━━━━━━━━━━━━━━━━━━━ 0s 570us/step
25/25 ━━━━━━━━━━━━━━━━━━━━ 0s 1ms/step
```

```
ExactExplainer explainer:  65%|███████████████████████████████████                   | 277/426 [02:02<01:00,  2.46it/s]
```

```
343/343 ━━━━━━━━━━━━━━━━━━━━ 0s 819us/step
25/25 ━━━━━━━━━━━━━━━━━━━━ 0s 1ms/step
```

```
ExactExplainer explainer:  65%|███████████████████████████████████▏                  | 278/426 [02:02<01:02,  2.37it/s]
```

```
355/355 ━━━━━━━━━━━━━━━━━━━━ 0s 739us/step
25/25 ━━━━━━━━━━━━━━━━━━━━ 0s 1ms/step
```

```
ExactExplainer explainer:  65%|███████████████████████████████████▎                  | 279/426 [02:03<01:04,  2.29it/s]
```

```
360/360 ━━━━━━━━━━━━━━━━━━━━ 0s 545us/step
25/25 ━━━━━━━━━━━━━━━━━━━━ 0s 2ms/step
```

```
ExactExplainer explainer:  66%|███████████████████████████████████▍                  | 280/426 [02:03<01:03,  2.31it/s]
```

```
358/358 ━━━━━━━━━━━━━━━━━━━━ 0s 529us/step
25/25 ━━━━━━━━━━━━━━━━━━━━ 0s 1ms/step
```

```
ExactExplainer explainer:  66%|███████████████████████████████████▌                  | 281/426 [02:04<01:00,  2.40it/s]
```

```
354/354 ━━━━━━━━━━━━━━━━━━━━ 0s 755us/step
25/25 ━━━━━━━━━━━━━━━━━━━━ 0s 1ms/step
```

```
ExactExplainer explainer:  66%|███████████████████████████████████▋                  | 282/426 [02:04<01:01,  2.33it/s]
```

```
351/351 ━━━━━━━━━━━━━━━━━━━━ 0s 462us/step
25/25 ━━━━━━━━━━━━━━━━━━━━ 0s 1ms/step
```

```
ExactExplainer explainer:  66%|███████████████████████████████████▊                  | 283/426 [02:04<00:58,  2.45it/s]
```

```
369/369 ━━━━━━━━━━━━━━━━━━━━ 0s 547us/step
25/25 ━━━━━━━━━━━━━━━━━━━━ 0s 2ms/step
```

```
ExactExplainer explainer:  67%|████████████████████████████████████                  | 284/426 [02:05<01:00,  2.34it/s]
```

```
368/368 ━━━━━━━━━━━━━━━━━━━━ 0s 549us/step
25/25 ━━━━━━━━━━━━━━━━━━━━ 0s 1ms/step
```

```
ExactExplainer explainer:  67%|████████████████████████████████████▏                 | 285/426 [02:05<01:00,  2.35it/s]
```

```
353/353 ━━━━━━━━━━━━━━━━━━━━ 0s 567us/step
25/25 ━━━━━━━━━━━━━━━━━━━━ 0s 1ms/step
```

```
ExactExplainer explainer:  67%|████████████████████████████████████▎                 | 286/426 [02:06<01:00,  2.30it/s]
```

```
337/337 ━━━━━━━━━━━━━━━━━━━━ 0s 829us/step
25/25 ━━━━━━━━━━━━━━━━━━━━ 0s 2ms/step
```

```
ExactExplainer explainer:  67%|████████████████████████████████████▍                 | 287/426 [02:06<01:06,  2.11it/s]
```

```
365/365 ━━━━━━━━━━━━━━━━━━━━ 0s 603us/step
25/25 ━━━━━━━━━━━━━━━━━━━━ 0s 1ms/step
```

```
ExactExplainer explainer:  68%|████████████████████████████████████▌                 | 288/426 [02:07<01:02,  2.21it/s]
```

```
348/348 ━━━━━━━━━━━━━━━━━━━━ 0s 551us/step
25/25 ━━━━━━━━━━━━━━━━━━━━ 0s 1ms/step
```

```
ExactExplainer explainer:  68%|████████████████████████████████████▋                 | 289/426 [02:07<01:00,  2.26it/s]
```

```
361/361 ━━━━━━━━━━━━━━━━━━━━ 0s 540us/step
25/25 ━━━━━━━━━━━━━━━━━━━━ 0s 1ms/step
```

```
ExactExplainer explainer:  68%|████████████████████████████████████▊                 | 290/426 [02:08<00:59,  2.29it/s]
```

```
355/355 ━━━━━━━━━━━━━━━━━━━━ 0s 531us/step
25/25 ━━━━━━━━━━━━━━━━━━━━ 0s 2ms/step
```

```
ExactExplainer explainer:  68%|████████████████████████████████████▉                 | 291/426 [02:08<00:58,  2.32it/s]
```

```
349/349 ━━━━━━━━━━━━━━━━━━━━ 0s 552us/step
25/25 ━━━━━━━━━━━━━━━━━━━━ 0s 1ms/step
```

```
ExactExplainer explainer:  69%|█████████████████████████████████████                 | 292/426 [02:08<00:57,  2.33it/s]
```

```
358/358 ━━━━━━━━━━━━━━━━━━━━ 0s 644us/step
25/25 ━━━━━━━━━━━━━━━━━━━━ 0s 1ms/step
```

```
ExactExplainer explainer:  69%|█████████████████████████████████████▏                | 293/426 [02:09<00:57,  2.31it/s]
```

```
368/368 ━━━━━━━━━━━━━━━━━━━━ 0s 480us/step
25/25 ━━━━━━━━━━━━━━━━━━━━ 0s 1ms/step
```

```
ExactExplainer explainer:  69%|█████████████████████████████████████▎                | 294/426 [02:09<00:55,  2.39it/s]
```

```
368/368 ━━━━━━━━━━━━━━━━━━━━ 0s 612us/step
25/25 ━━━━━━━━━━━━━━━━━━━━ 0s 2ms/step
```

```
ExactExplainer explainer:  69%|█████████████████████████████████████▍                | 295/426 [02:10<00:59,  2.21it/s]
```

```
368/368 ━━━━━━━━━━━━━━━━━━━━ 0s 602us/step
25/25 ━━━━━━━━━━━━━━━━━━━━ 0s 1ms/step
```

```
ExactExplainer explainer:  69%|█████████████████████████████████████▌                | 296/426 [02:10<01:00,  2.16it/s]
```

```
382/382 ━━━━━━━━━━━━━━━━━━━━ 0s 505us/step
25/25 ━━━━━━━━━━━━━━━━━━━━ 0s 1ms/step
```

```
ExactExplainer explainer:  70%|█████████████████████████████████████▋                | 297/426 [02:11<00:57,  2.26it/s]
```

```
353/353 ━━━━━━━━━━━━━━━━━━━━ 0s 493us/step
25/25 ━━━━━━━━━━━━━━━━━━━━ 0s 1ms/step
```

```
ExactExplainer explainer:  70%|█████████████████████████████████████▊                | 298/426 [02:11<00:53,  2.39it/s]
```

```
378/378 ━━━━━━━━━━━━━━━━━━━━ 0s 531us/step
25/25 ━━━━━━━━━━━━━━━━━━━━ 0s 1ms/step
```

```
ExactExplainer explainer:  70%|█████████████████████████████████████▉                | 299/426 [02:11<00:53,  2.38it/s]
```

```
372/372 ━━━━━━━━━━━━━━━━━━━━ 0s 600us/step
25/25 ━━━━━━━━━━━━━━━━━━━━ 0s 1ms/step
```

```
ExactExplainer explainer:  70%|██████████████████████████████████████                | 300/426 [02:12<00:54,  2.31it/s]
```

```
356/356 ━━━━━━━━━━━━━━━━━━━━ 0s 582us/step
25/25 ━━━━━━━━━━━━━━━━━━━━ 0s 1ms/step
```

```
ExactExplainer explainer:  71%|██████████████████████████████████████▏               | 301/426 [02:12<00:55,  2.25it/s]
```

```
367/367 ━━━━━━━━━━━━━━━━━━━━ 0s 571us/step
25/25 ━━━━━━━━━━━━━━━━━━━━ 0s 1ms/step
```

```
ExactExplainer explainer:  71%|██████████████████████████████████████▎               | 302/426 [02:13<00:56,  2.21it/s]
```

```
373/373 ━━━━━━━━━━━━━━━━━━━━ 0s 755us/step
25/25 ━━━━━━━━━━━━━━━━━━━━ 0s 1ms/step
```

```
ExactExplainer explainer:  71%|██████████████████████████████████████▍               | 303/426 [02:13<00:57,  2.15it/s]
```

```
377/377 ━━━━━━━━━━━━━━━━━━━━ 0s 524us/step
25/25 ━━━━━━━━━━━━━━━━━━━━ 0s 1ms/step
```

```
ExactExplainer explainer:  71%|██████████████████████████████████████▌               | 304/426 [02:14<00:53,  2.27it/s]
```

```
367/367 ━━━━━━━━━━━━━━━━━━━━ 0s 606us/step
25/25 ━━━━━━━━━━━━━━━━━━━━ 0s 2ms/step
```

```
ExactExplainer explainer:  72%|██████████████████████████████████████▋               | 305/426 [02:14<00:52,  2.31it/s]
```

```
361/361 ━━━━━━━━━━━━━━━━━━━━ 0s 805us/step
25/25 ━━━━━━━━━━━━━━━━━━━━ 0s 1ms/step
```

```
ExactExplainer explainer:  72%|██████████████████████████████████████▊               | 306/426 [02:15<00:55,  2.17it/s]
```

```
357/357 ━━━━━━━━━━━━━━━━━━━━ 0s 655us/step
25/25 ━━━━━━━━━━━━━━━━━━━━ 0s 2ms/step
```

```
ExactExplainer explainer:  72%|██████████████████████████████████████▉               | 307/426 [02:15<00:53,  2.21it/s]
```

```
355/355 ━━━━━━━━━━━━━━━━━━━━ 0s 659us/step
25/25 ━━━━━━━━━━━━━━━━━━━━ 0s 1ms/step
```

```
ExactExplainer explainer:  72%|███████████████████████████████████████               | 308/426 [02:16<00:54,  2.18it/s]
```

```
359/359 ━━━━━━━━━━━━━━━━━━━━ 0s 825us/step
25/25 ━━━━━━━━━━━━━━━━━━━━ 0s 1ms/step
```

```
ExactExplainer explainer:  73%|███████████████████████████████████████▏              | 309/426 [02:16<00:57,  2.03it/s]
```

```
353/353 ━━━━━━━━━━━━━━━━━━━━ 0s 410us/step
25/25 ━━━━━━━━━━━━━━━━━━━━ 0s 2ms/step
```

```
ExactExplainer explainer:  73%|███████████████████████████████████████▎              | 310/426 [02:17<00:53,  2.17it/s]
```

```
342/342 ━━━━━━━━━━━━━━━━━━━━ 0s 679us/step
25/25 ━━━━━━━━━━━━━━━━━━━━ 0s 1ms/step
```

```
ExactExplainer explainer:  73%|███████████████████████████████████████▍              | 311/426 [02:17<00:53,  2.17it/s]
```

```
350/350 ━━━━━━━━━━━━━━━━━━━━ 0s 552us/step
25/25 ━━━━━━━━━━━━━━━━━━━━ 0s 1ms/step
```

```
ExactExplainer explainer:  73%|███████████████████████████████████████▌              | 312/426 [02:17<00:50,  2.25it/s]
```

```
346/346 ━━━━━━━━━━━━━━━━━━━━ 0s 500us/step
25/25 ━━━━━━━━━━━━━━━━━━━━ 0s 1ms/step
```

```
ExactExplainer explainer:  73%|███████████████████████████████████████▋              | 313/426 [02:18<00:49,  2.30it/s]
```

```
362/362 ━━━━━━━━━━━━━━━━━━━━ 0s 491us/step
25/25 ━━━━━━━━━━━━━━━━━━━━ 0s 983us/step
```

```
ExactExplainer explainer:  74%|███████████████████████████████████████▊              | 314/426 [02:18<00:46,  2.39it/s]
```

```
347/347 ━━━━━━━━━━━━━━━━━━━━ 0s 520us/step
25/25 ━━━━━━━━━━━━━━━━━━━━ 0s 1ms/step
```

```
ExactExplainer explainer:  74%|███████████████████████████████████████▉              | 315/426 [02:19<00:44,  2.52it/s]
```

```
350/350 ━━━━━━━━━━━━━━━━━━━━ 0s 451us/step
25/25 ━━━━━━━━━━━━━━━━━━━━ 0s 1ms/step
```

```
ExactExplainer explainer:  74%|████████████████████████████████████████              | 316/426 [02:19<00:41,  2.63it/s]
```

```
358/358 ━━━━━━━━━━━━━━━━━━━━ 0s 596us/step
25/25 ━━━━━━━━━━━━━━━━━━━━ 0s 1ms/step
```

```
ExactExplainer explainer:  74%|████████████████████████████████████████▏             | 317/426 [02:19<00:42,  2.57it/s]
```

```
362/362 ━━━━━━━━━━━━━━━━━━━━ 0s 604us/step
25/25 ━━━━━━━━━━━━━━━━━━━━ 0s 1ms/step
```

```
ExactExplainer explainer:  75%|████████████████████████████████████████▎             | 318/426 [02:20<00:43,  2.50it/s]
```

```
367/367 ━━━━━━━━━━━━━━━━━━━━ 0s 476us/step
25/25 ━━━━━━━━━━━━━━━━━━━━ 0s 1ms/step
```

```
ExactExplainer explainer:  75%|████████████████████████████████████████▍             | 319/426 [02:20<00:43,  2.45it/s]
```

```
352/352 ━━━━━━━━━━━━━━━━━━━━ 0s 514us/step
25/25 ━━━━━━━━━━━━━━━━━━━━ 0s 1ms/step
```

```
ExactExplainer explainer:  75%|████████████████████████████████████████▌             | 320/426 [02:21<00:42,  2.48it/s]
```

```
355/355 ━━━━━━━━━━━━━━━━━━━━ 0s 604us/step
25/25 ━━━━━━━━━━━━━━━━━━━━ 0s 1ms/step
```

```
ExactExplainer explainer:  75%|████████████████████████████████████████▋             | 321/426 [02:21<00:43,  2.43it/s]
```

```
363/363 ━━━━━━━━━━━━━━━━━━━━ 0s 623us/step
25/25 ━━━━━━━━━━━━━━━━━━━━ 0s 1ms/step
```

```
ExactExplainer explainer:  76%|████████████████████████████████████████▊             | 322/426 [02:21<00:43,  2.40it/s]
```

```
360/360 ━━━━━━━━━━━━━━━━━━━━ 0s 837us/step
25/25 ━━━━━━━━━━━━━━━━━━━━ 0s 1ms/step
```

```
ExactExplainer explainer:  76%|████████████████████████████████████████▉             | 323/426 [02:22<00:46,  2.22it/s]
```

```
345/345 ━━━━━━━━━━━━━━━━━━━━ 0s 790us/step
25/25 ━━━━━━━━━━━━━━━━━━━━ 0s 1ms/step
```

```
ExactExplainer explainer:  76%|█████████████████████████████████████████             | 324/426 [02:22<00:46,  2.21it/s]
```

```
355/355 ━━━━━━━━━━━━━━━━━━━━ 0s 551us/step
25/25 ━━━━━━━━━━━━━━━━━━━━ 0s 2ms/step
```

```
ExactExplainer explainer:  76%|█████████████████████████████████████████▏            | 325/426 [02:23<00:45,  2.24it/s]
```

```
350/350 ━━━━━━━━━━━━━━━━━━━━ 0s 450us/step
25/25 ━━━━━━━━━━━━━━━━━━━━ 0s 2ms/step
```

```
ExactExplainer explainer:  77%|█████████████████████████████████████████▎            | 326/426 [02:23<00:43,  2.28it/s]
```

```
373/373 ━━━━━━━━━━━━━━━━━━━━ 0s 543us/step
25/25 ━━━━━━━━━━━━━━━━━━━━ 0s 2ms/step
```

```
ExactExplainer explainer:  77%|█████████████████████████████████████████▍            | 327/426 [02:24<00:44,  2.23it/s]
```

```
373/373 ━━━━━━━━━━━━━━━━━━━━ 0s 798us/step
25/25 ━━━━━━━━━━━━━━━━━━━━ 0s 1ms/step
```

```
ExactExplainer explainer:  77%|█████████████████████████████████████████▌            | 328/426 [02:25<00:55,  1.78it/s]
```

```
349/349 ━━━━━━━━━━━━━━━━━━━━ 0s 473us/step
25/25 ━━━━━━━━━━━━━━━━━━━━ 0s 1ms/step
```

```
ExactExplainer explainer:  77%|█████████████████████████████████████████▋            | 329/426 [02:25<00:49,  1.95it/s]
```

```
347/347 ━━━━━━━━━━━━━━━━━━━━ 0s 456us/step
25/25 ━━━━━━━━━━━━━━━━━━━━ 0s 1ms/step
```

```
ExactExplainer explainer:  77%|█████████████████████████████████████████▊            | 330/426 [02:25<00:44,  2.17it/s]
```

```
382/382 ━━━━━━━━━━━━━━━━━━━━ 0s 544us/step
25/25 ━━━━━━━━━━━━━━━━━━━━ 0s 1ms/step
```

```
ExactExplainer explainer:  78%|█████████████████████████████████████████▉            | 331/426 [02:26<00:42,  2.22it/s]
```

```
355/355 ━━━━━━━━━━━━━━━━━━━━ 0s 470us/step
25/25 ━━━━━━━━━━━━━━━━━━━━ 0s 885us/step
```

```
ExactExplainer explainer:  78%|██████████████████████████████████████████            | 332/426 [02:26<00:39,  2.36it/s]
```

```
350/350 ━━━━━━━━━━━━━━━━━━━━ 0s 641us/step
25/25 ━━━━━━━━━━━━━━━━━━━━ 0s 1ms/step
```

```
ExactExplainer explainer:  78%|██████████████████████████████████████████▏           | 333/426 [02:27<00:41,  2.24it/s]
```

```
378/378 ━━━━━━━━━━━━━━━━━━━━ 0s 681us/step
25/25 ━━━━━━━━━━━━━━━━━━━━ 0s 1ms/step
```

```
ExactExplainer explainer:  78%|██████████████████████████████████████████▎           | 334/426 [02:27<00:43,  2.13it/s]
```

```
364/364 ━━━━━━━━━━━━━━━━━━━━ 0s 537us/step
25/25 ━━━━━━━━━━━━━━━━━━━━ 0s 2ms/step
```

```
ExactExplainer explainer:  79%|██████████████████████████████████████████▍           | 335/426 [02:28<00:41,  2.21it/s]
```

```
350/350 ━━━━━━━━━━━━━━━━━━━━ 0s 473us/step
25/25 ━━━━━━━━━━━━━━━━━━━━ 0s 2ms/step
```

```
ExactExplainer explainer:  79%|██████████████████████████████████████████▌           | 336/426 [02:28<00:39,  2.27it/s]
```

```
358/358 ━━━━━━━━━━━━━━━━━━━━ 0s 407us/step
25/25 ━━━━━━━━━━━━━━━━━━━━ 0s 1ms/step
```

```
ExactExplainer explainer:  79%|██████████████████████████████████████████▋           | 337/426 [02:28<00:37,  2.37it/s]
```

```
360/360 ━━━━━━━━━━━━━━━━━━━━ 0s 508us/step
25/25 ━━━━━━━━━━━━━━━━━━━━ 0s 1ms/step
```

```
ExactExplainer explainer:  79%|██████████████████████████████████████████▊           | 338/426 [02:29<00:35,  2.47it/s]
```

```
375/375 ━━━━━━━━━━━━━━━━━━━━ 0s 491us/step
25/25 ━━━━━━━━━━━━━━━━━━━━ 0s 913us/step
```

```
ExactExplainer explainer:  80%|██████████████████████████████████████████▉           | 339/426 [02:29<00:37,  2.30it/s]
```

```
373/373 ━━━━━━━━━━━━━━━━━━━━ 0s 523us/step
25/25 ━━━━━━━━━━━━━━━━━━━━ 0s 949us/step
```

```
ExactExplainer explainer:  80%|███████████████████████████████████████████           | 340/426 [02:30<00:36,  2.37it/s]
```

```
356/356 ━━━━━━━━━━━━━━━━━━━━ 0s 618us/step
25/25 ━━━━━━━━━━━━━━━━━━━━ 0s 1ms/step
```

```
ExactExplainer explainer:  80%|███████████████████████████████████████████▏          | 341/426 [02:30<00:34,  2.44it/s]
```

```
354/354 ━━━━━━━━━━━━━━━━━━━━ 0s 453us/step
25/25 ━━━━━━━━━━━━━━━━━━━━ 0s 1ms/step
```

```
ExactExplainer explainer:  80%|███████████████████████████████████████████▎          | 342/426 [02:30<00:32,  2.58it/s]
```

```
353/353 ━━━━━━━━━━━━━━━━━━━━ 0s 476us/step
25/25 ━━━━━━━━━━━━━━━━━━━━ 0s 1ms/step
```

```
ExactExplainer explainer:  81%|███████████████████████████████████████████▍          | 343/426 [02:31<00:32,  2.59it/s]
```

```
350/350 ━━━━━━━━━━━━━━━━━━━━ 0s 460us/step
25/25 ━━━━━━━━━━━━━━━━━━━━ 0s 942us/step
```

```
ExactExplainer explainer:  81%|███████████████████████████████████████████▌          | 344/426 [02:31<00:30,  2.69it/s]
```

```
351/351 ━━━━━━━━━━━━━━━━━━━━ 0s 511us/step
25/25 ━━━━━━━━━━━━━━━━━━━━ 0s 1ms/step
```

```
ExactExplainer explainer:  81%|███████████████████████████████████████████▋          | 345/426 [02:31<00:30,  2.67it/s]
```

```
369/369 ━━━━━━━━━━━━━━━━━━━━ 0s 493us/step
25/25 ━━━━━━━━━━━━━━━━━━━━ 0s 2ms/step
```

```
ExactExplainer explainer:  81%|███████████████████████████████████████████▊          | 346/426 [02:32<00:31,  2.53it/s]
```

```
343/343 ━━━━━━━━━━━━━━━━━━━━ 0s 540us/step
25/25 ━━━━━━━━━━━━━━━━━━━━ 0s 1ms/step
```

```
ExactExplainer explainer:  81%|███████████████████████████████████████████▉          | 347/426 [02:32<00:31,  2.54it/s]
```

```
358/358 ━━━━━━━━━━━━━━━━━━━━ 0s 548us/step
25/25 ━━━━━━━━━━━━━━━━━━━━ 0s 1ms/step
```

```
ExactExplainer explainer:  82%|████████████████████████████████████████████          | 348/426 [02:33<00:32,  2.42it/s]
```

```
367/367 ━━━━━━━━━━━━━━━━━━━━ 0s 637us/step
25/25 ━━━━━━━━━━━━━━━━━━━━ 0s 1ms/step
```

```
ExactExplainer explainer:  82%|████████████████████████████████████████████▏         | 349/426 [02:33<00:33,  2.27it/s]
```

```
362/362 ━━━━━━━━━━━━━━━━━━━━ 0s 451us/step
25/25 ━━━━━━━━━━━━━━━━━━━━ 0s 1ms/step
```

```
ExactExplainer explainer:  82%|████████████████████████████████████████████▎         | 350/426 [02:34<00:31,  2.38it/s]
```

```
348/348 ━━━━━━━━━━━━━━━━━━━━ 0s 459us/step
25/25 ━━━━━━━━━━━━━━━━━━━━ 0s 1ms/step
```

```
ExactExplainer explainer:  82%|████████████████████████████████████████████▍         | 351/426 [02:34<00:30,  2.45it/s]
```

```
349/349 ━━━━━━━━━━━━━━━━━━━━ 0s 512us/step
25/25 ━━━━━━━━━━━━━━━━━━━━ 0s 1ms/step
```

```
ExactExplainer explainer:  83%|████████████████████████████████████████████▌         | 352/426 [02:34<00:29,  2.48it/s]
```

```
363/363 ━━━━━━━━━━━━━━━━━━━━ 0s 379us/step
25/25 ━━━━━━━━━━━━━━━━━━━━ 0s 971us/step
```

```
ExactExplainer explainer:  83%|████████████████████████████████████████████▋         | 353/426 [02:35<00:31,  2.35it/s]
```

```
353/353 ━━━━━━━━━━━━━━━━━━━━ 0s 460us/step
25/25 ━━━━━━━━━━━━━━━━━━━━ 0s 921us/step
```

```
ExactExplainer explainer:  83%|████████████████████████████████████████████▊         | 354/426 [02:35<00:28,  2.54it/s]
```

```
341/341 ━━━━━━━━━━━━━━━━━━━━ 0s 473us/step
25/25 ━━━━━━━━━━━━━━━━━━━━ 0s 1ms/step
```

```
ExactExplainer explainer:  83%|█████████████████████████████████████████████         | 355/426 [02:35<00:26,  2.66it/s]
```

```
348/348 ━━━━━━━━━━━━━━━━━━━━ 0s 442us/step
25/25 ━━━━━━━━━━━━━━━━━━━━ 0s 2ms/step
```

```
ExactExplainer explainer:  84%|█████████████████████████████████████████████▏        | 356/426 [02:36<00:26,  2.68it/s]
```

```
369/369 ━━━━━━━━━━━━━━━━━━━━ 0s 488us/step
25/25 ━━━━━━━━━━━━━━━━━━━━ 0s 1ms/step
```

```
ExactExplainer explainer:  84%|█████████████████████████████████████████████▎        | 357/426 [02:36<00:25,  2.68it/s]
```

```
359/359 ━━━━━━━━━━━━━━━━━━━━ 0s 560us/step
25/25 ━━━━━━━━━━━━━━━━━━━━ 0s 1ms/step
```

```
ExactExplainer explainer:  84%|█████████████████████████████████████████████▍        | 358/426 [02:37<00:25,  2.63it/s]
```

```
365/365 ━━━━━━━━━━━━━━━━━━━━ 0s 447us/step
25/25 ━━━━━━━━━━━━━━━━━━━━ 0s 1ms/step
```

```
ExactExplainer explainer:  84%|█████████████████████████████████████████████▌        | 359/426 [02:37<00:26,  2.57it/s]
```

```
358/358 ━━━━━━━━━━━━━━━━━━━━ 0s 446us/step
25/25 ━━━━━━━━━━━━━━━━━━━━ 0s 2ms/step
```

```
ExactExplainer explainer:  85%|█████████████████████████████████████████████▋        | 360/426 [02:37<00:25,  2.54it/s]
```

```
357/357 ━━━━━━━━━━━━━━━━━━━━ 0s 447us/step
25/25 ━━━━━━━━━━━━━━━━━━━━ 0s 1ms/step
```

```
ExactExplainer explainer:  85%|█████████████████████████████████████████████▊        | 361/426 [02:38<00:25,  2.60it/s]
```

```
371/371 ━━━━━━━━━━━━━━━━━━━━ 0s 482us/step
25/25 ━━━━━━━━━━━━━━━━━━━━ 0s 1ms/step
```

```
ExactExplainer explainer:  85%|█████████████████████████████████████████████▉        | 362/426 [02:38<00:24,  2.61it/s]
```

```
369/369 ━━━━━━━━━━━━━━━━━━━━ 0s 443us/step
25/25 ━━━━━━━━━━━━━━━━━━━━ 0s 1ms/step
```

```
ExactExplainer explainer:  85%|██████████████████████████████████████████████        | 363/426 [02:39<00:23,  2.67it/s]
```

```
378/378 ━━━━━━━━━━━━━━━━━━━━ 0s 480us/step
25/25 ━━━━━━━━━━━━━━━━━━━━ 0s 1ms/step
```

```
ExactExplainer explainer:  85%|██████████████████████████████████████████████▏       | 364/426 [02:39<00:23,  2.61it/s]
```

```
350/350 ━━━━━━━━━━━━━━━━━━━━ 0s 562us/step
25/25 ━━━━━━━━━━━━━━━━━━━━ 0s 1ms/step
```

```
ExactExplainer explainer:  86%|██████████████████████████████████████████████▎       | 365/426 [02:39<00:23,  2.59it/s]
```

```
384/384 ━━━━━━━━━━━━━━━━━━━━ 0s 460us/step
25/25 ━━━━━━━━━━━━━━━━━━━━ 0s 1ms/step
```

```
ExactExplainer explainer:  86%|██████████████████████████████████████████████▍       | 366/426 [02:40<00:25,  2.38it/s]
```

```
351/351 ━━━━━━━━━━━━━━━━━━━━ 0s 507us/step
25/25 ━━━━━━━━━━━━━━━━━━━━ 0s 908us/step
```

```
ExactExplainer explainer:  86%|██████████████████████████████████████████████▌       | 367/426 [02:40<00:24,  2.46it/s]
```

```
357/357 ━━━━━━━━━━━━━━━━━━━━ 0s 626us/step
25/25 ━━━━━━━━━━━━━━━━━━━━ 0s 1ms/step
```

```
ExactExplainer explainer:  86%|██████████████████████████████████████████████▋       | 368/426 [02:41<00:23,  2.45it/s]
```

```
362/362 ━━━━━━━━━━━━━━━━━━━━ 0s 507us/step
25/25 ━━━━━━━━━━━━━━━━━━━━ 0s 905us/step
```

```
ExactExplainer explainer:  87%|██████████████████████████████████████████████▊       | 369/426 [02:41<00:24,  2.33it/s]
```

```
352/352 ━━━━━━━━━━━━━━━━━━━━ 0s 519us/step
25/25 ━━━━━━━━━━━━━━━━━━━━ 0s 1ms/step
```

```
ExactExplainer explainer:  87%|██████████████████████████████████████████████▉       | 370/426 [02:42<00:24,  2.28it/s]
```

```
359/359 ━━━━━━━━━━━━━━━━━━━━ 0s 638us/step
25/25 ━━━━━━━━━━━━━━━━━━━━ 0s 886us/step
```

```
ExactExplainer explainer:  87%|███████████████████████████████████████████████       | 371/426 [02:42<00:24,  2.29it/s]
```

```
357/357 ━━━━━━━━━━━━━━━━━━━━ 0s 572us/step
25/25 ━━━━━━━━━━━━━━━━━━━━ 0s 1ms/step
```

```
ExactExplainer explainer:  87%|███████████████████████████████████████████████▏      | 372/426 [02:42<00:24,  2.19it/s]
```

```
350/350 ━━━━━━━━━━━━━━━━━━━━ 0s 513us/step
25/25 ━━━━━━━━━━━━━━━━━━━━ 0s 1ms/step
```

```
ExactExplainer explainer:  88%|███████████████████████████████████████████████▎      | 373/426 [02:43<00:23,  2.28it/s]
```

```
349/349 ━━━━━━━━━━━━━━━━━━━━ 0s 550us/step
25/25 ━━━━━━━━━━━━━━━━━━━━ 0s 2ms/step
```

```
ExactExplainer explainer:  88%|███████████████████████████████████████████████▍      | 374/426 [02:43<00:24,  2.14it/s]
```

```
355/355 ━━━━━━━━━━━━━━━━━━━━ 0s 571us/step
25/25 ━━━━━━━━━━━━━━━━━━━━ 0s 1ms/step
```

```
ExactExplainer explainer:  88%|███████████████████████████████████████████████▌      | 375/426 [02:44<00:22,  2.24it/s]
```

```
349/349 ━━━━━━━━━━━━━━━━━━━━ 0s 500us/step
25/25 ━━━━━━━━━━━━━━━━━━━━ 0s 2ms/step
```

```
ExactExplainer explainer:  88%|███████████████████████████████████████████████▋      | 376/426 [02:44<00:23,  2.14it/s]
```

```
378/378 ━━━━━━━━━━━━━━━━━━━━ 0s 540us/step
25/25 ━━━━━━━━━━━━━━━━━━━━ 0s 924us/step
```

```
ExactExplainer explainer:  88%|███████████████████████████████████████████████▊      | 377/426 [02:45<00:22,  2.23it/s]
```

```
347/347 ━━━━━━━━━━━━━━━━━━━━ 0s 552us/step
25/25 ━━━━━━━━━━━━━━━━━━━━ 0s 1ms/step
```

```
ExactExplainer explainer:  89%|███████████████████████████████████████████████▉      | 378/426 [02:45<00:20,  2.32it/s]
```

```
349/349 ━━━━━━━━━━━━━━━━━━━━ 0s 448us/step
25/25 ━━━━━━━━━━━━━━━━━━━━ 0s 964us/step
```

```
ExactExplainer explainer:  89%|████████████████████████████████████████████████      | 379/426 [02:45<00:19,  2.45it/s]
```

```
347/347 ━━━━━━━━━━━━━━━━━━━━ 0s 763us/step
25/25 ━━━━━━━━━━━━━━━━━━━━ 0s 1ms/step
```

```
ExactExplainer explainer:  89%|████████████████████████████████████████████████▏     | 380/426 [02:46<00:19,  2.38it/s]
```

```
373/373 ━━━━━━━━━━━━━━━━━━━━ 0s 695us/step
25/25 ━━━━━━━━━━━━━━━━━━━━ 0s 952us/step
```

```
ExactExplainer explainer:  89%|████████████████████████████████████████████████▎     | 381/426 [02:46<00:20,  2.24it/s]
```

```
367/367 ━━━━━━━━━━━━━━━━━━━━ 0s 552us/step
25/25 ━━━━━━━━━━━━━━━━━━━━ 0s 2ms/step
```

```
ExactExplainer explainer:  90%|████████████████████████████████████████████████▍     | 382/426 [02:47<00:19,  2.23it/s]
```

```
340/340 ━━━━━━━━━━━━━━━━━━━━ 0s 503us/step
25/25 ━━━━━━━━━━━━━━━━━━━━ 0s 1ms/step
```

```
ExactExplainer explainer:  90%|████████████████████████████████████████████████▌     | 383/426 [02:47<00:17,  2.39it/s]
```

```
352/352 ━━━━━━━━━━━━━━━━━━━━ 0s 434us/step
25/25 ━━━━━━━━━━━━━━━━━━━━ 0s 1ms/step
```

```
ExactExplainer explainer:  90%|████████████████████████████████████████████████▋     | 384/426 [02:48<00:16,  2.54it/s]
```

```
374/374 ━━━━━━━━━━━━━━━━━━━━ 0s 767us/step
25/25 ━━━━━━━━━━━━━━━━━━━━ 0s 1ms/step
```

```
ExactExplainer explainer:  90%|████████████████████████████████████████████████▊     | 385/426 [02:48<00:21,  1.93it/s]
```

```
349/349 ━━━━━━━━━━━━━━━━━━━━ 0s 447us/step
25/25 ━━━━━━━━━━━━━━━━━━━━ 0s 2ms/step
```

```
ExactExplainer explainer:  91%|████████████████████████████████████████████████▉     | 386/426 [02:49<00:18,  2.11it/s]
```

```
352/352 ━━━━━━━━━━━━━━━━━━━━ 0s 561us/step
25/25 ━━━━━━━━━━━━━━━━━━━━ 0s 908us/step
```

```
ExactExplainer explainer:  91%|█████████████████████████████████████████████████     | 387/426 [02:49<00:18,  2.08it/s]
```

```
358/358 ━━━━━━━━━━━━━━━━━━━━ 0s 491us/step
25/25 ━━━━━━━━━━━━━━━━━━━━ 0s 928us/step
```

```
ExactExplainer explainer:  91%|█████████████████████████████████████████████████▏    | 388/426 [02:50<00:17,  2.11it/s]
```

```
381/381 ━━━━━━━━━━━━━━━━━━━━ 0s 509us/step
25/25 ━━━━━━━━━━━━━━━━━━━━ 0s 1ms/step
```

```
ExactExplainer explainer:  91%|█████████████████████████████████████████████████▎    | 389/426 [02:50<00:16,  2.26it/s]
```

```
356/356 ━━━━━━━━━━━━━━━━━━━━ 0s 476us/step
25/25 ━━━━━━━━━━━━━━━━━━━━ 0s 1ms/step
```

```
ExactExplainer explainer:  92%|█████████████████████████████████████████████████▍    | 390/426 [02:51<00:16,  2.22it/s]
```

```
357/357 ━━━━━━━━━━━━━━━━━━━━ 0s 450us/step
25/25 ━━━━━━━━━━━━━━━━━━━━ 0s 2ms/step
```

```
ExactExplainer explainer:  92%|█████████████████████████████████████████████████▌    | 391/426 [02:51<00:15,  2.30it/s]
```

```
369/369 ━━━━━━━━━━━━━━━━━━━━ 0s 528us/step
25/25 ━━━━━━━━━━━━━━━━━━━━ 0s 1ms/step
```

```
ExactExplainer explainer:  92%|█████████████████████████████████████████████████▋    | 392/426 [02:51<00:14,  2.40it/s]
```

```
349/349 ━━━━━━━━━━━━━━━━━━━━ 0s 511us/step
25/25 ━━━━━━━━━━━━━━━━━━━━ 0s 1000us/step
```

```
ExactExplainer explainer:  92%|█████████████████████████████████████████████████▊    | 393/426 [02:52<00:13,  2.48it/s]
```

```
355/355 ━━━━━━━━━━━━━━━━━━━━ 0s 461us/step
25/25 ━━━━━━━━━━━━━━━━━━━━ 0s 1ms/step
```

```
ExactExplainer explainer:  92%|█████████████████████████████████████████████████▉    | 394/426 [02:52<00:12,  2.60it/s]
```

```
336/336 ━━━━━━━━━━━━━━━━━━━━ 0s 478us/step
25/25 ━━━━━━━━━━━━━━━━━━━━ 0s 1ms/step
```

```
ExactExplainer explainer:  93%|██████████████████████████████████████████████████    | 395/426 [02:52<00:11,  2.70it/s]
```

```
375/375 ━━━━━━━━━━━━━━━━━━━━ 0s 646us/step
25/25 ━━━━━━━━━━━━━━━━━━━━ 0s 1ms/step
```

```
ExactExplainer explainer:  93%|██████████████████████████████████████████████████▏   | 396/426 [02:53<00:11,  2.50it/s]
```

```
340/340 ━━━━━━━━━━━━━━━━━━━━ 0s 484us/step
25/25 ━━━━━━━━━━━━━━━━━━━━ 0s 1ms/step
```

```
ExactExplainer explainer:  93%|██████████████████████████████████████████████████▎   | 397/426 [02:53<00:11,  2.59it/s]
```

```
356/356 ━━━━━━━━━━━━━━━━━━━━ 0s 481us/step
25/25 ━━━━━━━━━━━━━━━━━━━━ 0s 1ms/step
```

```
ExactExplainer explainer:  93%|██████████████████████████████████████████████████▍   | 398/426 [02:54<00:10,  2.59it/s]
```

```
348/348 ━━━━━━━━━━━━━━━━━━━━ 0s 448us/step
25/25 ━━━━━━━━━━━━━━━━━━━━ 0s 2ms/step
```

```
ExactExplainer explainer:  94%|██████████████████████████████████████████████████▌   | 399/426 [02:54<00:10,  2.60it/s]
```

```
366/366 ━━━━━━━━━━━━━━━━━━━━ 0s 564us/step
25/25 ━━━━━━━━━━━━━━━━━━━━ 0s 1ms/step
```

```
ExactExplainer explainer:  94%|██████████████████████████████████████████████████▋   | 400/426 [02:54<00:10,  2.52it/s]
```

```
363/363 ━━━━━━━━━━━━━━━━━━━━ 0s 491us/step
25/25 ━━━━━━━━━━━━━━━━━━━━ 0s 1ms/step
```

```
ExactExplainer explainer:  94%|██████████████████████████████████████████████████▊   | 401/426 [02:55<00:10,  2.34it/s]
```

```
367/367 ━━━━━━━━━━━━━━━━━━━━ 0s 397us/step
25/25 ━━━━━━━━━━━━━━━━━━━━ 0s 1ms/step
```

```
ExactExplainer explainer:  94%|██████████████████████████████████████████████████▉   | 402/426 [02:55<00:10,  2.24it/s]
```

```
375/375 ━━━━━━━━━━━━━━━━━━━━ 0s 481us/step
25/25 ━━━━━━━━━━━━━━━━━━━━ 0s 1ms/step
```

```
ExactExplainer explainer:  95%|███████████████████████████████████████████████████   | 403/426 [02:56<00:09,  2.37it/s]
```

```
357/357 ━━━━━━━━━━━━━━━━━━━━ 0s 495us/step
25/25 ━━━━━━━━━━━━━━━━━━━━ 0s 1ms/step
```

```
ExactExplainer explainer:  95%|███████████████████████████████████████████████████▏  | 404/426 [02:56<00:09,  2.27it/s]
```

```
356/356 ━━━━━━━━━━━━━━━━━━━━ 0s 543us/step
25/25 ━━━━━━━━━━━━━━━━━━━━ 0s 923us/step
```

```
ExactExplainer explainer:  95%|███████████████████████████████████████████████████▎  | 405/426 [02:57<00:08,  2.37it/s]
```

```
361/361 ━━━━━━━━━━━━━━━━━━━━ 0s 441us/step
25/25 ━━━━━━━━━━━━━━━━━━━━ 0s 2ms/step
```

```
ExactExplainer explainer:  95%|███████████████████████████████████████████████████▍  | 406/426 [02:57<00:08,  2.48it/s]
```

```
363/363 ━━━━━━━━━━━━━━━━━━━━ 0s 511us/step
25/25 ━━━━━━━━━━━━━━━━━━━━ 0s 1ms/step
```

```
ExactExplainer explainer:  96%|███████████████████████████████████████████████████▌  | 407/426 [02:57<00:07,  2.50it/s]
```

```
350/350 ━━━━━━━━━━━━━━━━━━━━ 0s 514us/step
25/25 ━━━━━━━━━━━━━━━━━━━━ 0s 1ms/step
```

```
ExactExplainer explainer:  96%|███████████████████████████████████████████████████▋  | 408/426 [02:58<00:07,  2.52it/s]
```

```
360/360 ━━━━━━━━━━━━━━━━━━━━ 0s 717us/step
25/25 ━━━━━━━━━━━━━━━━━━━━ 0s 1ms/step
```

```
ExactExplainer explainer:  96%|███████████████████████████████████████████████████▊  | 409/426 [02:58<00:07,  2.32it/s]
```

```
354/354 ━━━━━━━━━━━━━━━━━━━━ 0s 444us/step
25/25 ━━━━━━━━━━━━━━━━━━━━ 0s 932us/step
```

```
ExactExplainer explainer:  96%|███████████████████████████████████████████████████▉  | 410/426 [02:59<00:07,  2.25it/s]
```

```
372/372 ━━━━━━━━━━━━━━━━━━━━ 0s 481us/step
25/25 ━━━━━━━━━━━━━━━━━━━━ 0s 922us/step
```

```
ExactExplainer explainer:  96%|████████████████████████████████████████████████████  | 411/426 [02:59<00:06,  2.41it/s]
```

```
334/334 ━━━━━━━━━━━━━━━━━━━━ 0s 443us/step
25/25 ━━━━━━━━━━━━━━━━━━━━ 0s 975us/step
```

```
ExactExplainer explainer:  97%|████████████████████████████████████████████████████▏ | 412/426 [03:00<00:06,  2.29it/s]
```

```
372/372 ━━━━━━━━━━━━━━━━━━━━ 0s 504us/step
25/25 ━━━━━━━━━━━━━━━━━━━━ 0s 2ms/step
```

```
ExactExplainer explainer:  97%|████████████████████████████████████████████████████▎ | 413/426 [03:00<00:05,  2.33it/s]
```

```
371/371 ━━━━━━━━━━━━━━━━━━━━ 0s 581us/step
25/25 ━━━━━━━━━━━━━━━━━━━━ 0s 910us/step
```

```
ExactExplainer explainer:  97%|████████████████████████████████████████████████████▍ | 414/426 [03:00<00:05,  2.38it/s]
```

```
351/351 ━━━━━━━━━━━━━━━━━━━━ 0s 623us/step
25/25 ━━━━━━━━━━━━━━━━━━━━ 0s 947us/step
```

```
ExactExplainer explainer:  97%|████████████████████████████████████████████████████▌ | 415/426 [03:01<00:04,  2.39it/s]
```

```
353/353 ━━━━━━━━━━━━━━━━━━━━ 0s 478us/step
25/25 ━━━━━━━━━━━━━━━━━━━━ 0s 953us/step
```

```
ExactExplainer explainer:  98%|████████████████████████████████████████████████████▋ | 416/426 [03:01<00:04,  2.47it/s]
```

```
349/349 ━━━━━━━━━━━━━━━━━━━━ 0s 476us/step
25/25 ━━━━━━━━━━━━━━━━━━━━ 0s 1ms/step
```

```
ExactExplainer explainer:  98%|████████████████████████████████████████████████████▊ | 417/426 [03:02<00:03,  2.34it/s]
```

```
378/378 ━━━━━━━━━━━━━━━━━━━━ 0s 470us/step
25/25 ━━━━━━━━━━━━━━━━━━━━ 0s 1ms/step
```

```
ExactExplainer explainer:  98%|████████████████████████████████████████████████████▉ | 418/426 [03:02<00:03,  2.43it/s]
```

```
348/348 ━━━━━━━━━━━━━━━━━━━━ 0s 622us/step
25/25 ━━━━━━━━━━━━━━━━━━━━ 0s 1ms/step
```

```
ExactExplainer explainer:  98%|█████████████████████████████████████████████████████ | 419/426 [03:02<00:02,  2.43it/s]
```

```
388/388 ━━━━━━━━━━━━━━━━━━━━ 0s 666us/step
25/25 ━━━━━━━━━━━━━━━━━━━━ 0s 1ms/step
```

```
ExactExplainer explainer:  99%|█████████████████████████████████████████████████████▏| 420/426 [03:03<00:02,  2.29it/s]
```

```
392/392 ━━━━━━━━━━━━━━━━━━━━ 0s 493us/step
25/25 ━━━━━━━━━━━━━━━━━━━━ 0s 1ms/step
```

```
ExactExplainer explainer:  99%|█████████████████████████████████████████████████████▎| 421/426 [03:03<00:02,  2.21it/s]
```

```
362/362 ━━━━━━━━━━━━━━━━━━━━ 0s 624us/step
25/25 ━━━━━━━━━━━━━━━━━━━━ 0s 957us/step
```

```
ExactExplainer explainer:  99%|█████████████████████████████████████████████████████▍| 422/426 [03:04<00:01,  2.16it/s]
```

```
361/361 ━━━━━━━━━━━━━━━━━━━━ 0s 536us/step
25/25 ━━━━━━━━━━━━━━━━━━━━ 0s 1ms/step
```

```
ExactExplainer explainer:  99%|█████████████████████████████████████████████████████▌| 423/426 [03:04<00:01,  2.26it/s]
```

```
368/368 ━━━━━━━━━━━━━━━━━━━━ 0s 504us/step
25/25 ━━━━━━━━━━━━━━━━━━━━ 0s 949us/step
```

```
ExactExplainer explainer: 100%|█████████████████████████████████████████████████████▋| 424/426 [03:05<00:00,  2.31it/s]
```

```
357/357 ━━━━━━━━━━━━━━━━━━━━ 0s 426us/step
25/25 ━━━━━━━━━━━━━━━━━━━━ 0s 971us/step
```

```
ExactExplainer explainer: 100%|█████████████████████████████████████████████████████▊| 425/426 [03:05<00:00,  2.50it/s]
```

```
368/368 ━━━━━━━━━━━━━━━━━━━━ 0s 474us/step
25/25 ━━━━━━━━━━━━━━━━━━━━ 0s 1ms/step
```

```
ExactExplainer explainer: 100%|██████████████████████████████████████████████████████| 426/426 [03:05<00:00,  2.38it/s]
```

```
381/381 ━━━━━━━━━━━━━━━━━━━━ 0s 644us/step
25/25 ━━━━━━━━━━━━━━━━━━━━ 0s 1ms/step
```

```
ExactExplainer explainer: 427it [03:06,  2.24it/s]
```

In [15]:

```
#ploting SHAP values

vals = np.abs(shap_values.values).mean(0)
feature_names = shap_values.feature_names

# rikiuojam pagal svarbą
sorted_idx = np.argsort(vals)

fig, ax = plt.subplots()

bars = ax.barh(
    np.array(feature_names)[sorted_idx],
    vals[sorted_idx],
    color=plt.get_cmap("viridis")(0.8)  # viena spalva visiems barams
)

ax.set_xlabel("Mean absolute SHAP value")

# užrašom reikšmes ant barų
for bar in bars:
    width = bar.get_width()
    ax.text(
        width/2,                       # x koordinatė – stulpelio ilgis
        bar.get_y() + bar.get_height()/2, # y koordinatė – stulpelio vidurys
        f"{width:.3f}",                  # užrašoma reikšmė (pvz. 3 skaičiai po kablelio)
        va="center", ha="left",          # vertikalus ir horizontalus lygiavimas
        fontsize=9, color="black"
    )

plt.show()
```

In [16]:

```
#SHAP summary plot

shap.initjs()

plt.title("SHAP summary plot (dot)")
shap.summary_plot(shap_values.values, test_features, feature_names=test_features.columns, cmap="viridis")
```

```
C:\Users\azied\AppData\Local\Temp\ipykernel_81144\2590342463.py:4: FutureWarning: The NumPy global RNG was seeded by calling `np.random.seed`. In a future version this function will no longer use the global RNG. Pass `rng` explicitly to opt-in to the new behaviour and silence this warning.
  shap.summary_plot(shap_values.values, test_features, feature_names=test_features.columns, cmap="viridis")
```

In [17]:

```
#SHAP dependence plots

for i in range(len(IndVars)-1):
    feature_name = test_features.columns[i]
    shap.dependence_plot(feature_name,
                    shap_values.values,
                    test_features,
                    feature_names=test_features.columns,
                    cmap=plt.get_cmap("viridis"),
                    show=False
                    # , interaction_index=None                   #nerodyti interakcijų su labiausiai tikėtinu moderatoriumi
                    )
    plt.axhline(y=0, color="black", linestyle="dashed", linewidth=0.5)
    plt.show()
```

In [18]:

```
#Permutation feature importance score
from sklearn.metrics import r2_score
import numpy as np
import matplotlib.pyplot as plt

# === 1️⃣ Funkcija svarbos skaičiavimui ===
def permutation_importance_tf(model, X, y, n_repeats=10, random_state=42):
    rng = np.random.default_rng(random_state)
    X_values = X.values if hasattr(X, "values") else np.array(X)
    y_values = y.values if hasattr(y, "values") else np.array(y)
    
    # bazinis R² su originaliais duomenimis
    base_pred = model.predict(X_values).ravel()
    base_r2 = r2_score(y_values, base_pred)
    
    importances = np.zeros(X_values.shape[1])

    for i in range(X_values.shape[1]):
        scores = []
        for _ in range(n_repeats):
            X_perm = X_values.copy()
            X_perm[:, i] = rng.permutation(X_perm[:, i])  # sumaišo vieną stulpelį
            perm_pred = model.predict(X_perm).ravel()
            r2_perm = r2_score(y_values, perm_pred)
            scores.append(base_r2 - r2_perm)  # kiek pablogėjo R²
        importances[i] = np.mean(scores)
    
    return importances

# === 2️⃣ Skaičiuojame svarbą ===
importances = permutation_importance_tf(model, test_features, test_labels, n_repeats=10)
indices = np.argsort(importances)[::-1]

# === 3️⃣ Išvedame TOP-10 prediktorių ===
print("\nTOP-20 svarbiausių prediktorių:")
for i in indices:
    print(f"{test_features.columns[i]} importance = {importances[i]:.4f}")

# === 4️⃣ (Nebūtina, bet naudinga) — grafikas ===
top_n = min(20, len(importances))  # jei kintamųjų mažiau nei 10

plt.figure(figsize=(8, 5))
plt.barh(range(top_n), importances[indices[:top_n]][::-1])
plt.yticks(range(top_n), [test_features.columns[i] for i in indices[:top_n]][::-1])
plt.xlabel("Permutation Importance (ΔR²)")
plt.title("Svarbiausi prediktoriai pagal ANN modelį")
plt.tight_layout()
plt.show()
```

```
14/14 ━━━━━━━━━━━━━━━━━━━━ 0s 2ms/step 
14/14 ━━━━━━━━━━━━━━━━━━━━ 0s 2ms/step 
14/14 ━━━━━━━━━━━━━━━━━━━━ 0s 2ms/step 
14/14 ━━━━━━━━━━━━━━━━━━━━ 0s 3ms/step 
14/14 ━━━━━━━━━━━━━━━━━━━━ 0s 2ms/step 
14/14 ━━━━━━━━━━━━━━━━━━━━ 0s 2ms/step 
14/14 ━━━━━━━━━━━━━━━━━━━━ 0s 2ms/step 
14/14 ━━━━━━━━━━━━━━━━━━━━ 0s 2ms/step 
14/14 ━━━━━━━━━━━━━━━━━━━━ 0s 2ms/step 
14/14 ━━━━━━━━━━━━━━━━━━━━ 0s 2ms/step 
14/14 ━━━━━━━━━━━━━━━━━━━━ 0s 2ms/step 
14/14 ━━━━━━━━━━━━━━━━━━━━ 0s 2ms/step 
14/14 ━━━━━━━━━━━━━━━━━━━━ 0s 2ms/step 
14/14 ━━━━━━━━━━━━━━━━━━━━ 0s 3ms/step 
14/14 ━━━━━━━━━━━━━━━━━━━━ 0s 2ms/step 
14/14 ━━━━━━━━━━━━━━━━━━━━ 0s 2ms/step 
14/14 ━━━━━━━━━━━━━━━━━━━━ 0s 2ms/step 
14/14 ━━━━━━━━━━━━━━━━━━━━ 0s 1ms/step 
14/14 ━━━━━━━━━━━━━━━━━━━━ 0s 2ms/step 
14/14 ━━━━━━━━━━━━━━━━━━━━ 0s 2ms/step 
14/14 ━━━━━━━━━━━━━━━━━━━━ 0s 2ms/step 
14/14 ━━━━━━━━━━━━━━━━━━━━ 0s 3ms/step 
14/14 ━━━━━━━━━━━━━━━━━━━━ 0s 1ms/step 
14/14 ━━━━━━━━━━━━━━━━━━━━ 0s 2ms/step 
14/14 ━━━━━━━━━━━━━━━━━━━━ 0s 2ms/step 
14/14 ━━━━━━━━━━━━━━━━━━━━ 0s 2ms/step 
14/14 ━━━━━━━━━━━━━━━━━━━━ 0s 2ms/step 
14/14 ━━━━━━━━━━━━━━━━━━━━ 0s 2ms/step 
14/14 ━━━━━━━━━━━━━━━━━━━━ 0s 2ms/step 
14/14 ━━━━━━━━━━━━━━━━━━━━ 0s 2ms/step 
14/14 ━━━━━━━━━━━━━━━━━━━━ 0s 1ms/step 
14/14 ━━━━━━━━━━━━━━━━━━━━ 0s 2ms/step 
14/14 ━━━━━━━━━━━━━━━━━━━━ 0s 2ms/step 
14/14 ━━━━━━━━━━━━━━━━━━━━ 0s 2ms/step 
14/14 ━━━━━━━━━━━━━━━━━━━━ 0s 2ms/step 
14/14 ━━━━━━━━━━━━━━━━━━━━ 0s 2ms/step 
14/14 ━━━━━━━━━━━━━━━━━━━━ 0s 1ms/step 
14/14 ━━━━━━━━━━━━━━━━━━━━ 0s 2ms/step 
14/14 ━━━━━━━━━━━━━━━━━━━━ 0s 2ms/step 
14/14 ━━━━━━━━━━━━━━━━━━━━ 0s 1ms/step 
14/14 ━━━━━━━━━━━━━━━━━━━━ 0s 1ms/step 
14/14 ━━━━━━━━━━━━━━━━━━━━ 0s 2ms/step 
14/14 ━━━━━━━━━━━━━━━━━━━━ 0s 1ms/step 
14/14 ━━━━━━━━━━━━━━━━━━━━ 0s 1ms/step 
14/14 ━━━━━━━━━━━━━━━━━━━━ 0s 2ms/step 
14/14 ━━━━━━━━━━━━━━━━━━━━ 0s 2ms/step 
14/14 ━━━━━━━━━━━━━━━━━━━━ 0s 2ms/step 
14/14 ━━━━━━━━━━━━━━━━━━━━ 0s 2ms/step 
14/14 ━━━━━━━━━━━━━━━━━━━━ 0s 3ms/step 
14/14 ━━━━━━━━━━━━━━━━━━━━ 0s 3ms/step 
14/14 ━━━━━━━━━━━━━━━━━━━━ 0s 2ms/step 
14/14 ━━━━━━━━━━━━━━━━━━━━ 0s 2ms/step 
14/14 ━━━━━━━━━━━━━━━━━━━━ 0s 1ms/step 
14/14 ━━━━━━━━━━━━━━━━━━━━ 0s 2ms/step 
14/14 ━━━━━━━━━━━━━━━━━━━━ 0s 2ms/step 
14/14 ━━━━━━━━━━━━━━━━━━━━ 0s 1ms/step 
14/14 ━━━━━━━━━━━━━━━━━━━━ 0s 1ms/step 
14/14 ━━━━━━━━━━━━━━━━━━━━ 0s 2ms/step 
14/14 ━━━━━━━━━━━━━━━━━━━━ 0s 2ms/step 
14/14 ━━━━━━━━━━━━━━━━━━━━ 0s 2ms/step 
14/14 ━━━━━━━━━━━━━━━━━━━━ 0s 2ms/step 
14/14 ━━━━━━━━━━━━━━━━━━━━ 0s 2ms/step 
14/14 ━━━━━━━━━━━━━━━━━━━━ 0s 2ms/step 
14/14 ━━━━━━━━━━━━━━━━━━━━ 0s 2ms/step 
14/14 ━━━━━━━━━━━━━━━━━━━━ 0s 2ms/step 
14/14 ━━━━━━━━━━━━━━━━━━━━ 0s 2ms/step 
14/14 ━━━━━━━━━━━━━━━━━━━━ 0s 2ms/step 
14/14 ━━━━━━━━━━━━━━━━━━━━ 0s 1ms/step 
14/14 ━━━━━━━━━━━━━━━━━━━━ 0s 2ms/step 
14/14 ━━━━━━━━━━━━━━━━━━━━ 0s 3ms/step 
14/14 ━━━━━━━━━━━━━━━━━━━━ 0s 2ms/step 

TOP-20 svarbiausių prediktorių:
amzius importance = 0.1587
Pdarbu importance = 0.1493
Burnout importance = 0.0591
Vparama importance = 0.0233
Konfliktai importance = 0.0191
Vkonflikt importance = 0.0180
Tobul importance = 0.0156
```

In [19]:

```
# === 3️⃣ Išvedame TOP-10 prediktorių ===
print("\nPrediktoriai pagal svarbą:")
for i in indices:
    print(f"{test_features.columns[i]:<20} importance = {importances[i]:.4f}")

# === 4️⃣ (Nebūtina, bet naudinga) — grafikas ===
top_n = len(importances)

plt.figure(figsize=(8, 5))
plt.barh(range(top_n), importances[indices[:top_n]][::-1])
plt.yticks(range(top_n), [test_features.columns[i] for i in indices[:top_n]][::-1])
plt.xlabel("Permutation Importance (ΔR²)")
plt.title("Svarbiausi prediktoriai pagal ANN modelį")
plt.tight_layout()
plt.show()
```

```
Prediktoriai pagal svarbą:
amzius               importance = 0.1587
Pdarbu               importance = 0.1493
Burnout              importance = 0.0591
Vparama              importance = 0.0233
Konfliktai           importance = 0.0191
Vkonflikt            importance = 0.0180
Tobul                importance = 0.0156
```

In [21]:

```
#SHAP summary plot in English

feature_names_en = ["Age", "Job_satisfaction", "Burnout", "Role_conflict", "Int_conflicts", "Opp_for_development", "Manager_support"]
shap.initjs()

plt.title("SHAP summary plot (dot)")
shap.summary_plot(shap_values.values, test_features, feature_names=feature_names_en, cmap="viridis")
```

```
C:\Users\azied\AppData\Local\Temp\ipykernel_81144\4003191406.py:5: FutureWarning: The NumPy global RNG was seeded by calling `np.random.seed`. In a future version this function will no longer use the global RNG. Pass `rng` explicitly to opt-in to the new behaviour and silence this warning.
  shap.summary_plot(shap_values.values, test_features, feature_names=feature_names_en, cmap="viridis")
```

In [25]:

```
#SHAP dependence plots in English

for i in range(len(IndVars)-1):
    feature_name = feature_names_en[i]
    shap.dependence_plot(feature_name,
                    shap_values.values,
                    test_features,
                    feature_names=feature_names_en,
                    cmap=plt.get_cmap("viridis"),
                    show=False
                    # , interaction_index=None                   #nerodyti interakcijų su labiausiai tikėtinu moderatoriumi
                    )
    plt.axhline(y=0, color="black", linestyle="dashed", linewidth=0.5)
    plt.show()
```
