# Supplementary material for: Exploring the Predictors of Nurses’ Turnover Intentions Through Neural Network Modeling: A National Cross-Sectional Study in Lithuania
Source: Healthcare (Basel). 2026 Mar 24;14(7):831. doi: 10.3390/healthcare14070831 (PMC13073792; doi:10.3390/healthcare14070831)
Supplement: Supplementary file 1 [file healthcare-14-00831-s001.zip › Code S2.html]

Turnover\_linear\_reg


**Code S2.**

*Linear regression with sklearn for nurses' turnover intentions*

In [1]:

```
import random
import matplotlib.pyplot as plt
import numpy as np
import pandas as pd
import seaborn as sns
from sklearn.metrics import r2_score, mean_absolute_error, mean_squared_error
from sklearn.preprocessing import StandardScaler

seed = 1
random.seed(seed)
np.random.seed(seed)
```

In [2]:

```
#datafile and variables

DataFile = "C:\\Users\\azied\\Desktop\\Slaugytojų_2025_duomenys\\Nurse_ML(recode).csv"
IndVars = ["amzius",
           "Pdarbu", "Burnout",
           "Vkonflikt", "Konfliktai",
           "Tobul","Vparama"]
DepVar = "Turnover"

TotVars = IndVars
TotVars.append(DepVar)

raw_dataset = pd.read_csv(DataFile)
for col in raw_dataset.columns:
    raw_dataset[col] = pd.to_numeric(raw_dataset[col], errors='coerce')  #konvertuoja į skaičius (kitkas - NaN)
dataset = raw_dataset[TotVars].copy()
dataset = dataset.sample(frac=1).reset_index(drop=True)
dataset.tail()
```

Out[2]:

|  | amzius | Pdarbu | Burnout | Vkonflikt | Konfliktai | Tobul | Vparama | Turnover |
| --- | --- | --- | --- | --- | --- | --- | --- | --- |
| 3773 | 28.0 | 4.0 | 2.916667 | 3.000000 | 2.5 | 3.75 | NaN | 2.666667 |
| 3774 | 60.0 | 3.0 | 2.666667 | 3.333333 | 2.0 | 3.50 | 2.0 | 2.666667 |
| 3775 | NaN | NaN | NaN | 2.666667 | NaN | 3.50 | NaN | NaN |
| 3776 | NaN | NaN | NaN | NaN | NaN | NaN | NaN | NaN |
| 3777 | NaN | NaN | NaN | NaN | NaN | NaN | NaN | NaN |

In [3]:

```
#droping missing values
dataset = dataset.dropna()
print("Pašalintos praleistos reikšmės")
print("Likusi bendra imtis:", len(dataset))
```

```
Pašalintos praleistos reikšmės
Likusi bendra imtis: 2129
```

In [4]:

```
#separating training and test datasets

train_dataset = dataset.sample(frac=0.8, random_state=seed)
test_dataset = dataset.drop(train_dataset.index)

train_features = train_dataset.copy()
test_features = test_dataset.copy()

train_labels = train_features.pop(DepVar)
test_labels = test_features.pop(DepVar)
```

In [5]:

```
#standarizing
scaler = StandardScaler()
train_features_scaled = scaler.fit_transform(train_features)
test_features_scaled = scaler.transform(test_features)
```

In [6]:

```
from sklearn.linear_model import LinearRegression
```

In [7]:

```
reg = LinearRegression().fit(train_features_scaled, train_labels)
```

In [8]:

```
pred = reg.predict(test_features_scaled)
```

In [9]:

```
r_sqr = r2_score(test_labels, pred)
mae = mean_absolute_error(test_labels, pred)
rmse = np.sqrt(mean_squared_error(test_labels, pred))
```

In [10]:

```
print("R^2:", r_sqr)
print("MAE:", mae)
print("RMSE:", rmse)
```

```
R^2: 0.4109399072875979
MAE: 0.6240631664602496
RMSE: 0.7703123777603567
```

In [11]:

```
print("Intercept:", reg.intercept_)


coeff_df = pd.DataFrame({"Feature": test_features.columns, "Coefficient": reg.coef_})
print("\nFeature Coefficients:\n", coeff_df)
```

```
Intercept: 2.3814836562928163

Feature Coefficients:
       Feature  Coefficient
0      amzius    -0.237911
1      Pdarbu    -0.205079
2     Burnout     0.186588
3   Vkonflikt     0.073638
4  Konfliktai     0.096614
5       Tobul    -0.077900
6     Vparama    -0.141111
```

In [12]:

```
# Sort dataframe by coefficients.
coeff_df["abs_coeff"] = abs(coeff_df["Coefficient"])
coef_df_sorted = coeff_df.sort_values(by="abs_coeff", ascending=True)


# Create plot.
plt.figure(figsize=(8,6))
plt.barh(coef_df_sorted["Feature"], coef_df_sorted["Coefficient"], color="blue")
plt.xlabel("Coefficient Value")
plt.ylabel("Feature")
plt.title("Feature Importance (Linear Regression Coefficients)")
plt.show()
```
