# Supplementary material for: Exploring the Predictors of Nurses’ Turnover Intentions Through Neural Network Modeling: A National Cross-Sectional Study in Lithuania
Source: Healthcare (Basel). 2026 Mar 24;14(7):831. doi: 10.3390/healthcare14070831 (PMC13073792; doi:10.3390/healthcare14070831)
Supplement: Supplementary file 1 [file healthcare-14-00831-s001.zip › Table S1.pdf]

Table S1. Study measures and sample items

Construct	No. of items	Sample item	Instrument
Turnover intentions	3	"I intend to stay in this job for the foreseeable future"	Irving et al., 1997
Subjective health	1	"How would you rate your overall physical health?"	Standard question, widely used in international surveys (e.g., Eurofound, 2017; ESS ERIC, 2024)
Workability	1	"If you were to rate your work capacity on a 10-point scale, taking into account your physical and mental health, where 10 means that you are fully able to work, 0 - completely unable to work, how would you rate your work capacity at the moment?"	Job Demands-Resources (JD-R) questionnaire (Schaufeli, 2015)
Job satisfaction	1	"How satisfied are you overall with your job?"	Standard question, widely used in international surveys (e.g., Eurofound, 2017; ESS ERIC, 2024)
Burnout	12	“At work, I feel mentally exhausted”	Burnout Assessment Tool (BAT; Schaufeli et al., 2019)
Workload	4	"Do you have too much work?"	Job Demands-Resources (JD-R) questionnaire (Schaufeli, 2015)
Emotional demands	1	"Is your job emotionally demanding?"	
Physical demands	1	"Is your job physically demanding?"	
Cognitive demands	1	"Does your job require a lot of focus and concentration?"	
Red tape bureaucracy	3	"Do you have to follow excessive rules, procedures, or regulations at work?"	
Role conflict	3	"Do you have to do things at work that you would really like to do differently?"	
Interpersonal conflicts	2	"Do you conflict with anyone at work?"	
Bullying	1	„In the last 12 months, have you experienced any inappropriate treatment while performing your work, such as harassment, insults, threats, psychological or physical violence, etc.?“	The item was generated specifically for this study, based on focus group interviews.
Skill use	1	"Do you have enough opportunities to use your skills and abilities at work?"	Job Demands-Resources (JD-R) questionnaire (Schaufeli, 2015)
Task variety	2	"Is your job varied enough?"	
Participation	1	"Can you participate in making decisions about your job?"	
Opportunities for development	4	"I always learn new things in my work"	
Managerial support	3	"Can you count on help and support from your supervisor when needed?"	
Support from colleagues	3	"Can you count on help and support from colleagues when needed?"	
Recognition	1	"Do you feel valued by the people you work for (society,	

		patients, patients' relatives)?"	
Work control	7	"Can you decide how to do your job?"	
Tool availability	1	"I have all the tools I need to do my job"	
Reciprocity	1	"I feel that I am adequately compensated for my efforts"	
Pay	1	"I am paid enough for the work I do"	
Staffing and Resource Adequacy	4	"There are enough nursing staff to perform all tasks"	The Practice Environment Scale of the Nursing Work Index (Lake, 2002)
Nurse Manager Ability	5	"The nursing administrator is a good manager and leader"	
Collegial Nurse–Physician Relations	3	"Nurses and physicians work as a team"	
Satisfaction with physical environment	1	„I am satisfied with my physical work environment (furniture, lighting, equipment, etc.)“	The items were generated specifically for this study, based on focus group interviews
Extra benefits	1	„Does your institution provide you with additional social benefits (for example, catering services, parking, sports club membership, discounts on goods and services, health insurance, childcare, etc.)?“	
