# Supplementary material for: Exploring the Predictors of Nurses’ Turnover Intentions Through Neural Network Modeling: A National Cross-Sectional Study in Lithuania
Source: Healthcare (Basel). 2026 Mar 24;14(7):831. doi: 10.3390/healthcare14070831 (PMC13073792; doi:10.3390/healthcare14070831)
Supplement: Supplementary file 1 [file healthcare-14-00831-s001.zip › Table S2.pdf]

Table S2. Descriptive statistics and correlations between study variables

	M	SD	1	2	3	4	5	6	7	8	9	10	11	12	13	14
**1. Level**	2,0	0,8	—													
**2. Sector**	0,1	0,2	-.235***	—												
**3. Age**	48,4	12,2	-.037	-.163***	—											
**4. Education**	2,1	1,0	.137***	.049*	-.416***	—										
**5. Experience**	24,6	14,0	-.003	-.162***	.878***	-.364***	—									
**6. Factual workload**	1,1	0,2	.188***	-.022	-.012	.135***	.003	—								
**7. Physical health**	3,6	0,8	-.009	-.006	.01	.061**	-.019	.048*	—							
**8. Workability**	8,2	1,7	.007	-.042*	.074***	-.021	.048*	.051*	.545***	—						
**9. Job satisfaction**	3,8	0,8	-.050*	.005	.143***	-.050*	.099***	.003	.346***	.382***	—					
**10. Burnout**	2,2	0,6	.031	.013	-.225***	.106***	-.180***	-.002	-.395***	-.433***	-.504***	(.894)				
**11. Emotional demands**	3,9	0,9	.100***	-.103***	-.008	.019	-.009	.071***	-.181***	-.160***	-.273***	.398***	—			
**12. Physical demands**	3,2	1,2	.302***	-.100***	-.100***	.004	-.112***	.095***	-.154***	-.110***	-.199***	.284***	.443***	—		
**13. Cognitive demands**	4,6	0,6	.057**	-.026	.050*	-.01	.057**	.065**	-.005	.029	-.01	.054**	.342***	.189***	—	
**14. IWB**	0,5	0,5	.169***	-.023	-.184***	.089***	-.173***	.064**	-.183***	-.175***	-.264***	.352***	.327***	.261***	.105***	—
**15. Workload**	3,6	0,7	.053**	-.003	-.103***	.135***	-.103***	.052*	-.130***	-.134***	-.248***	.381***	.514***	.373***	.329***	.277***
**16. Red tape**	3,7	0,7	.085***	-.064**	-.052*	.095***	-.04	.060**	-.107***	-.098***	-.203***	.306***	.447***	.324***	.353***	.226***
**17. Role conflict**	2,7	0,9	.109***	-.043*	-.157***	.142***	-.141***	.064**	-.217***	-.233***	-.359***	.518***	.482***	.386***	.186***	.375***
**18. Interpersonal conflicts**	2,0	0,7	.137***	-.02	-.104***	.108***	-.080***	.111***	-.115***	-.135***	-.189***	.384***	.212***	.204***	.015	.232***
**19. Skill use**	4,0	0,9	-.044*	.03	.132***	-.094***	.101***	.024	.154***	.195***	.271***	-.257***	-.100***	-.047*	.146***	-.164***
**20. Participation**	3,4	1,0	-.091***	.070***	-.038	.053**	-.033	.077***	.113***	.114***	.225***	-.164***	-.087***	-.060**	.035	-.168***
**21. Tool availability**	3,8	1,0	-.124***	.068***	.080***	-.03	.080***	-.01	.191***	.213***	.329***	-.333***	-.250***	-.240***	-.025	-.277***
**22. SPE**	3,3	1,2	-.157***	.071***	.131***	-.038	.118***	-.049*	.227***	.224***	.346***	-.361***	-.288***	-.264***	-.067***	-.327***
**23. Reciprocity**	2,6	1,1	-.174***	.058**	.081***	-.041*	.063**	-.051*	.181***	.161***	.326***	-.260***	-.315***	-.289***	-.145***	-.265***
**24. Pay**	2,5	1,1	-.174***	.063**	.064**	-.023	.051*	-.053**	.172***	.170***	.335***	-.248***	-.309***	-.291***	-.147***	-.247***
**25. Extra benefits**	0,3	0,5	.123***	.052**	-.079***	.135***	-.074***	.051*	.077***	.080***	.135***	-.114***	-.089***	-.014	-.009	-.016

M - mean, SD - standard deviation, IWB – inappropriate workplace behavior, SPE - Satisfaction with physical environment. Where relevant, internal consistency indices (Cronbach alphas) are presented in parentheses on the diagonal of the table. *p < .05, **p < .01, ***p < .001.

Table S2. Cont.

	M	SD	1	2	3	4	5	6	7	8	9	10	11	12	13	14
**26. Recognition**	3,4	1,0	-.120***	.050*	.195***	-.082***	.141***	-.029	.181***	.225***	.356***	-.345***	-.226***	-.176***	-.028	-.222***
**27. Task variety**	3,9	0,8	.068***	.027	-.001	.063**	.018	.084***	.038	.077***	.085***	-.028	.220***	.238***	.283***	.056**
**28. Development opportunities**	3,5	0,9	-.047*	.005	.118***	-.047*	.094***	.044*	.184***	.237***	.393***	-.282***	-.106***	-.070***	.093***	-.229***
**29. Manager support**	3,3	1,1	-.136***	.063**	.045*	-.016	.039	.026	.242***	.246***	.384***	-.313***	-.264***	-.249***	-.061**	-.324***
**30. Colleague support**	3,8	0,9	-.044*	.041*	.088***	-.077***	.079***	-.001	.184***	.224***	.335***	-.314***	-.145***	-.086***	.031	-.217***
**31. Job control**	3,1	0,9	-.104***	.031	.125***	-.035	.099***	.052*	.141***	.173***	.291***	-.249***	-.199***	-.186***	-.055**	-.220***
**32. Staff adequacy**	2,4	0,7	-.151***	.076***	.119***	-.080***	.097***	-.041*	.220***	.228***	.358***	-.377***	-.375***	-.360***	-.151***	-.335***
**33. Nursing manager ability**	2,8	0,7	-.092***	.024	.088***	-.031	.073***	.012	.226***	.268***	.434***	-.356***	-.280***	-.234***	-.067***	-.347***
**34. NPR**	2,8	0,6	-.107***	.097***	.047*	-.067***	.038	0	.219***	.251***	.393***	-.363***	-.265***	-.196***	-.04	-.312***
**35. Turnover**	2,4	1,0	.103***	.064**	-.345***	.214***	-.301***	.037	-.223***	-.262***	-.470***	.488***	.258***	.216***	.024	.323***

M - mean, SD - standard deviation, NPR - nurse-physician relationships. *p < .05, **p < .01, ***p < .001.

Table S2. Cont.

	15	16	17	18	19	20	21	22	23	24	25	26	27	28	29
**15. Workload**	(.687)														
**16. Red tape**	.472***	(.656)													
**17. Role conflict**	.515***	.553***	(.811)												
**18. Interpersonal conflicts**	.176***	.195***	.335***	(.652)											
**19. Skill use**	-.073***	-.058**	-.255***	-.092***	—										
**20. Participation**	-.050*	-.035	-.145***	-.029	.335***	—									
**21. Tool availability**	-.232***	-.204***	-.501***	-.183***	.297***	.217***	—								
**22. SPE**	-.259***	-.237***	-.433***	-.211***	.239***	.229***	.576***	—							
**23. Reciprocity**	-.290***	-.261***	-.368***	-.129***	.150***	.189***	.367***	.443***	—						
**24. Pay**	-.287***	-.264***	-.368***	-.122***	.150***	.185***	.345***	.412***	.909***	—					
**25. Extra benefits**	-.047*	-.046*	-.126***	-.036	.056**	.087***	.114***	.121***	.081***	.094***	—				
**26. Recognition**	-.178***	-.160***	-.290***	-.117***	.262***	.229***	.273***	.317***	.274***	.268***	.088***	—			
**27. Task variety**	.280***	.257***	.156***	.053**	.306***	.262***	.068***	.036	-.022	-.013	.065**	.101***	(.673)		
**28. Development opportunities**	-.117***	-.078***	-.272***	-.080***	.419***	.397***	.347***	.351***	.277***	.269***	.125***	.361***	.355***	(.901)	
**29. Manager support**	-.234***	-.239***	-.409***	-.142***	.277***	.361***	.375***	.403***	.369***	.364***	.181***	.395***	.094***	.478***	(.877)
**30. Colleague support**	-.131***	-.140***	-.292***	-.194***	.294***	.225***	.276***	.279***	.194***	.195***	.119***	.437***	.164***	.381***	.440***
**31. Job control**	-.198***	-.190***	-.276***	-.032	.264***	.404***	.273***	.307***	.278***	.281***	.106***	.379***	.119***	.422***	.410***
**32. Staff adequacy**	-.414***	-.331***	-.486***	-.150***	.218***	.227***	.415***	.445***	.470***	.474***	.091***	.318***	-.068***	.286***	.401***
**33. Nursing manager ability**	-.251***	-.244***	-.432***	-.195***	.262***	.338***	.442***	.453***	.396***	.394***	.187***	.375***	.084***	.474***	.697***
**34. NPR**	-.244***	-.215***	-.378***	-.221***	.231***	.250***	.342***	.390***	.289***	.282***	.147***	.335***	.064**	.371***	.454***
**35. Turnover**	.275***	.223***	.395***	.276***	-.239***	-.166***	-.283***	-.315***	-.306***	-.299***	-.057**	-.278***	-.040*	-.338***	-.367***

SPE - Satisfaction with physical environment, NPR - nurse-physician relationships. Where relevant, internal consistency indices (Cronbach alphas) are presented in parentheses on the diagonal of the table. *$p < .05$, **$p < .01$, ***$p < .001$.

Table S2. Cont.

	30	31	32	33	34	35
**30. Colleague support**	(.845)					
**31. Job control**	.332***	(.888)				
**32. Staff adequacy**	.268***	.374***	(.877)			
**33. Nursing manager ability**	.398***	.433***	.513***	(.882)		
**34. NPR**	.419***	.338***	.436***	.563***	(.925)	
**35. Turnover**	-.275***	-.239***	-.319***	-.400***	-.304***	(.865)

NPR - nurse-physician relationships. Where relevant, internal consistency indices (Cronbach alphas) are presented in parentheses on the diagonal of the table. *p < .05, **p < .01, ***p < .001.
